# Structural Aspects of MoS_*x*_ Prepared by Atomic Layer Deposition for Hydrogen Evolution Reaction

**DOI:** 10.1021/acscatal.4c01445

**Published:** 2024-06-20

**Authors:** Miika Mattinen, Wei Chen, Rebecca A. Dawley, Marcel A. Verheijen, Emiel J. M. Hensen, W. M. M. Kessels, Ageeth A. Bol

**Affiliations:** † Department of Applied Physics and Science Education, Eindhoven University of Technology, P.O. Box 513, 5600 MB Eindhoven, The Netherlands; ‡ Department of Chemical Engineering and Chemistry, Eindhoven University of Technology, P.O. Box 513, 5600 MB Eindhoven, The Netherlands; § Department of Chemistry, University of Michigan, 930 N. University Avenue, Ann Arbor, Michigan 48109-1055, United States; ∥ Eurofins Materials Science Netherlands, High Tech Campus 11, 5656 AE Eindhoven, The Netherlands

**Keywords:** hydrogen evolution reaction, electrocatalyst, water splitting, molybdenum sulfide, atomic layer deposition, electrochemical activation

## Abstract

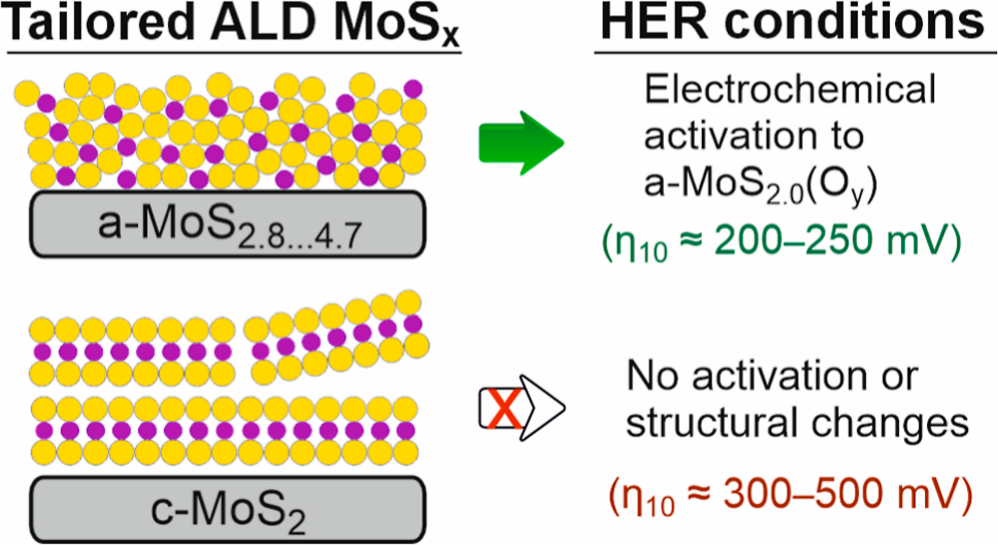

Molybdenum sulfides (MoS_*x*_) in both crystalline and amorphous forms are promising earth-abundant electrocatalysts for hydrogen evolution reaction (HER) in acid. Plasma-enhanced atomic layer deposition was used to prepare thin films of both amorphous MoS_*x*_ with adjustable S/Mo ratio (2.8–4.7) and crystalline MoS_2_ with tailored crystallinity, morphology, and electrical properties. All the amorphous MoS_*x*_ films transform into highly HER-active amorphous MoS_2_ (overpotential 210–250 mV at 10 mA/cm^2^ in 0.5 M H_2_SO_4_) after electrochemical activation at approximately −0.3 V vs reversible hydrogen electrode. However, the initial film stoichiometry affects the structure and consequently the HER activity and stability. The material changes occurring during activation are studied using ex situ and quasi in situ X-ray photoelectron spectroscopy. Possible structures of as-deposited and activated catalysts are proposed. In contrast to amorphous MoS_*x*_, no changes in the structure of crystalline MoS_2_ catalysts are observed. The overpotentials of the crystalline films range from 300 to 520 mV at 10 mA/cm^2^, being the lowest for the most defective catalysts. This work provides a practical method for deposition of tailored MoS_*x*_ HER electrocatalysts as well as new insights into their activity and structure.

## 1 Introduction

The climate crisis requires us to rapidly decrease our dependence on fossil fuels. Hydrogen (H_2_) can be used as a clean fuel, precursor in diverse industries including chemical and steel, as well as for stabilizing intermittent renewable energy production. However, currently, the vast majority of H_2_ is produced from fossil fuels.^1^ Cleaner routes to H_2_ are intensively pursued, of which electrochemical water splitting using renewable electricity is one of the top contenders. Besides affordable, clean electricity, electrochemical water splitting requires active, stable, and affordable electrocatalysts. Under acidic conditions encountered in high-performance proton-exchange membrane electrolyzers, expensive and scarce platinum group metal catalysts are currently used.^2^ Molybdenum sulfide (MoS_*x*_) is a promising, earth-abundant catalyst for hydrogen evolution reaction (HER) in acid, one of the two-half reactions of water splitting.

MoS_*x*_ exists in different forms. In layered crystalline form (c-MoS_2_), molybdenum disulfide is one of the best-known two-dimensional (2D) materials. Its stable form is a semiconducting, hexagonal (2H) phase, while a semiconducting rhombohedral (3R) phase and a metallic trigonal (1T) phase are also known.^3,4^ Amorphous MoS_*x*_ (a-MoS_*x*_) is best known at MoS_3_ stoichiometry,^5^ although it has been synthesized in stoichiometries ranging from MoS_2_ to MoS_6_.^6−8^ This flexibility in stoichiometry is enabled by the ability of sulfur to form (S–S)^2–^ pairs and polysulfide S_*n*_^2–^ chains, thus accommodating oxidation states −2, −1, and 0, while molybdenum is usually present as +4 in MoS_*x*_.

c-MoS_2_ has been intensively studied as an HER catalyst. While 2H-MoS_2_ in the bulk form has poor HER activity,^9,10^ polycrystalline, flat MoS_2_ films exhibit improved, yet comparably modest performance.^10,11^ As shown in 2007,^12^ only the edges of pristine MoS_2_ crystallites are active, while the basal planes of MoS_2_ are rather inactive toward HER. Thus, highly active 2H-MoS_2_ catalysts maximize the edge-to-basal plane ratio and/or activate the basal planes for HER via doping, defects, strain, or phase engineering.^13−17^ Many methods have been used to prepare c-MoS_2_ HER catalysts, including exfoliation of bulk crystals^18^ and gas-phase deposition methods such as chemical vapor deposition.^16^ Regardless, most of the available methods share at least one of the following limitations: poor thickness control, limited scalability, and harsh deposition conditions (e.g., high temperatures). The metallic 1T phase is more active for HER^19^ than 2H-MoS_2_, but it is difficult to prepare and unstable.^20^ The 3R phase has only been investigated in bulk form showing modest HER performance that is, however, improved over bulk 2H MoS_2_.^21,22^

Compared to 2H-MoS_2_, a-MoS_*x*_ inherently contains a higher density of active sites. The reported excellent activity and availability of low-cost, low-temperature preparation methods make a-MoS_*x*_ a highly promising HER catalyst.^23−25^ Yet, what constitutes an ideal a-MoS_*x*_ HER catalyst is still an open question. The structure of a-MoS_*x*_ is complex and depends on its stoichiometry and preparation method, and there is no consensus on its HER active sites. Studies on a-MoS_*x*_ are further complicated by changes in its structure and stoichiometry under HER conditions. While several studies have reported that a-MoS_*x*_ transforms into a more active form with a S/Mo ratio of ∼2 or less,^26−31^ other studies have claimed little to no S loss during HER.^7,8,32^ Furthermore, there are conflicting reports on whether the starting stoichiometry affects the activity of the activated a-MoS_*x*_ catalyst^7,8^ or not.^24,33^

The available deposition methods offer limited control over stoichiometry and other properties of a-MoS_*x*_. Typical methods include electrodeposition, which allows for a few different stoichiometries depending on the precursor^8^ and the potential range^8,26,27,34^ (*x* ≈ 2, 3, 4, and 6), wet chemical synthesis being largely limited by available precursors (*x* ≈ 3, 4, and 6),^7,35^ and solvothermal synthesis that may result in different stoichiometries and even mixtures of a-MoS_*x*_ and c-MoS_2_ within a single batch.^36^ Other reported methods include sputtering,^29^ pulsed laser deposition,^37^ and thermolysis of Mo salts.^38^ Many studies have utilized high catalyst loadings in ill-defined morphologies due to the limitations of the deposition methods used, which makes it challenging to identify and separate the catalytically active surface species from the inactive “bulk” material. Furthermore, the same methods typically cannot be used to deposit both a-MoS_*x*_ and c-MoS_2_.

Atomic layer deposition (ALD) is an advanced, surface-controlled gas phase thin-film deposition method. Based on self-limiting surface reactions, ALD boasts excellent large-area uniformity, reproducibility, scalability, and Å-level thickness control.^39−41^ ALD has been used to deposit both amorphous^42−44^ and crystalline^45,46^ MoS_2_ films for HER. Regardless, most of the available ALD processes offer limited control over stoichiometry and crystallinity of MoS_*x*_.^47^ Plasma-enhanced ALD (PEALD) offers more freedom in tailoring film properties,^48−51^ which also enables control of HER performance.^46,50,51^ Recently, we have shown that controlling the plasma chemistry via feed gas composition enables tailoring stoichiometry, crystallinity, morphology, and electronic properties of MoS_*x*_ within a broad range.^49^

In this work, we use PEALD to prepare both amorphous MoS_*x*_ thin films with a controlled S/Mo ratio (seven stoichiometries in the range of 2.8–4.7) as well as crystalline films with tailored properties including crystallinity, defectivity (e.g., S vacancies), and morphology. We discuss the effects of plasma chemistry and deposition temperature on film properties and show that the achieved wide range of properties enables identifying the most favorable types of MoS_*x*_ for HER. To understand what makes MoS_*x*_ active for HER, we have performed both electrochemical measurements and structural and compositional characterization of 17 different MoS_*x*_ thin films (∼7 nm thickness). Changes in the structure of amorphous MoS_*x*_ under HER conditions are further studied using X-ray photoelectron spectroscopy in both ex situ and quasi in situ manner. These insights are linked to catalyst stability studies and the literature to provide new insights into highly active a-MoS_*x*_ electrocatalysts.

## 2 Results and Discussion

### 2.1 Tailored Deposition of MoS_*x*_ Using PEALD

**Figure 1:**
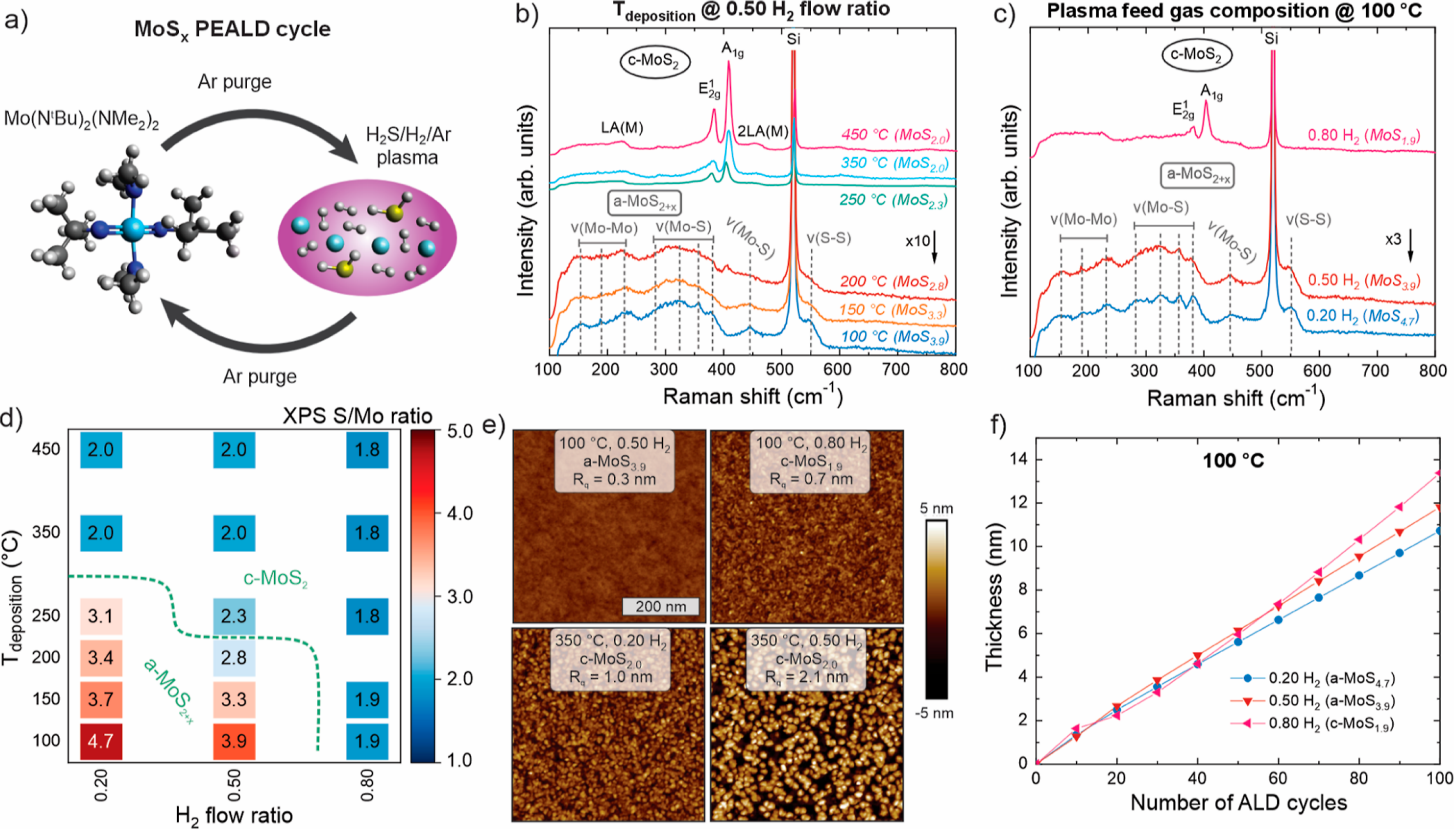
(a) Schematic of the PEALD cycle. Raman spectra and XPS stoichiometries of MoS_*x*_ films deposited (b) at different temperatures with a H_2_ flow ratio of 0.50 and (c) with different H_2_ flow ratios (plasma feed gas compositions) at 100 °C. Some of the spectra have been multiplied by the indicated factors. (d) Heatmap of S/Mo ratio (analyzed by XPS) as a function of deposition temperature and H_2_ flow ratio. The dashed green line indicates the border between amorphous and crystalline films according to Raman spectroscopy. (e) Atomic force microscopy (AFM) images and root-mean-square roughnesses (*R*_q_) of films deposited in selected conditions. (f) Thickness evolution determined by in situ spectroscopic ellipsometry (SE) for selected conditions. Film thicknesses were approximately 11–12 nm (16 nm for 100 °C) in (b), 15–18 nm in (c), and 7 nm in (d,e). The films were deposited on SiO_2_/Si to facilitate characterization. XPS measurements were also performed on films on GC, resulting in compositions identical to those on SiO_2_/Si.

We prepared thin-film MoS_*x*_ electrocatalysts with controlled stoichiometry, crystallinity, morphology, and thickness using PEALD. PEALD is a cyclical deposition process in which one cycle consists of alternately exposing the substrate to two precursors, here a metalorganic molybdenum precursor Mo(N^t^Bu)_2_(NMe_2_)_2_ and mixed H_2_S/H_2_/Ar plasma (Figure 1a).^46,49^ These precursors react on the substrate surface in a self-limiting manner. The precursor pulses are separated by Ar purge steps to eliminate gas-phase reactions. The surface-controlled nature of PEALD ensures excellent uniformity over large areas and complex features. Accurate thickness control is achieved by repeating the ALD cycle multiple times (Figure 1f).

We have identified two deposition parameters that can be used to tailor MoS_*x*_ film properties within a wide range: deposition temperature and plasma feed gas composition. To describe the effect of deposition temperature, we first consider a H_2_S/H_2_/Ar plasma feed gas with an intermediate amount of H_2_ (vide infra). At low deposition temperatures of 100–200 °C, amorphous, sulfur-rich films with S/Mo ratios of 2.8–3.9 are obtained (denoted a-MoS_2+*x*_). Their Raman spectra exhibit broad peaks that can be attributed to Mo–Mo, Mo–S, and S–S vibrations (Figure 1b).^5,27,31^ These modes are characteristic of a-MoS_*x*_ (2 < *x* < 6) that consists of a network of Mo_*x*_S_*y*_ clusters and/or chains containing Mo^4+^ together with S^2–^ and S_2_^2–^ species (refs (5, 7, 8, 31, 32, 50, and 53) and Section 2.4). Increasing the deposition temperature to 250 °C (with a fixed plasma feed gas composition) decreases the S/Mo ratio of the deposited films to approximately 2 (Figure 1b). This film exhibits the characteristic *E*^1^_2g_ and *A*_1g_ and defect-induced LA(M) Raman modes of crystalline, layered MoS_2_.^54−56^ A further increase in temperature up to 450 °C improves crystallinity as shown by the increased intensity of the MoS_2_ Raman modes. Besides the deposition temperature, the H_2_ flow ratio in the plasma feed gas (eq 1) can be used to control film stoichiometry. Increasing the H_2_ flow ratio decreases the S/Mo ratio of the deposited films. Using a sufficiently high H_2_ flow ratio (0.80), crystalline MoS_2_ films can be deposited at temperatures as low as 100 °C (Figure 1c).
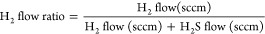
1

By systematically controlling both the deposition temperature and H_2_ flow ratio, we can tailor the stoichiometry, structure, crystallinity, and morphology of MoS_*x*_ films within a broad range.^49^Figure 1d illustrates the stoichiometry control and its correlation to crystallinity. The S/Mo ratio of amorphous films varied in the range of 2.8–4.7. For crystalline films, the S/Mo stoichiometry decreased slightly with increasing H_2_ flow ratio in the range of 2.3–1.8. By controlling the deposition temperature and H_2_ flow ratio, the morphology of the c-MoS_2_ films can also be varied. Most of the crystalline films were rather rough due to the formation of out-of-plane oriented crystallites (“fins”), a common occurrence for (PE)ALD TMDCs.^46,47,49,57,58^ The fin height and consequently the film roughness generally increase with increasing deposition temperature and H_2_ flow ratio (Figure 1e). All the a-MoS_2+*x*_ films, in contrast, were smooth with roughness values close to that of the substrate (SiO_2_/Si or glassy carbon, GC). Finally, Figure 1f illustrates how the film thickness can be accurately controlled for each deposition condition.

### 2.2 Systematic Evaluation of HER Activity of MoS_*x*_

For evaluation as HER catalysts, we prepared a series of MoS_*x*_ films at different deposition temperatures (100–450 °C) and H_2_ flow ratios (0.20, 0.50, and 0.80). The thickness of the films deposited on GC substrates was adjusted to ∼7 nm based on preliminary experiments, which corresponds to a catalyst loading of 2–3 μg/cm^2^ (Table S1 and Section S3 in the Supporting Information).

**Figure 2:**
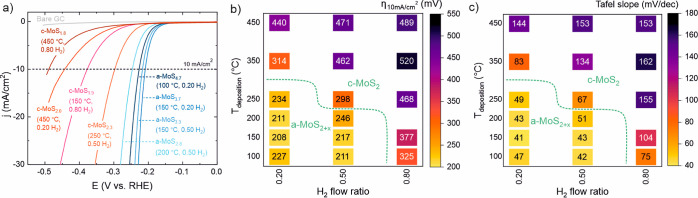
HER activity of MoS_*x*_ films as shown by (a) cyclic voltammetry (CV) scans of selected films [only forward (cathodic) trace of fifth CV is shown for clarity] and heatmaps of (b) overpotential required to reach a current density of 10 mA/cm^2^ and (c) Tafel slope. The dashed green line indicates the border between amorphous and crystalline films. Films deposited to a thickness of 7 nm on GC substrates were used, and the values were determined from the forward trace of the fifth CV scan after 100% *iR* drop compensation.

**Figure 3:**
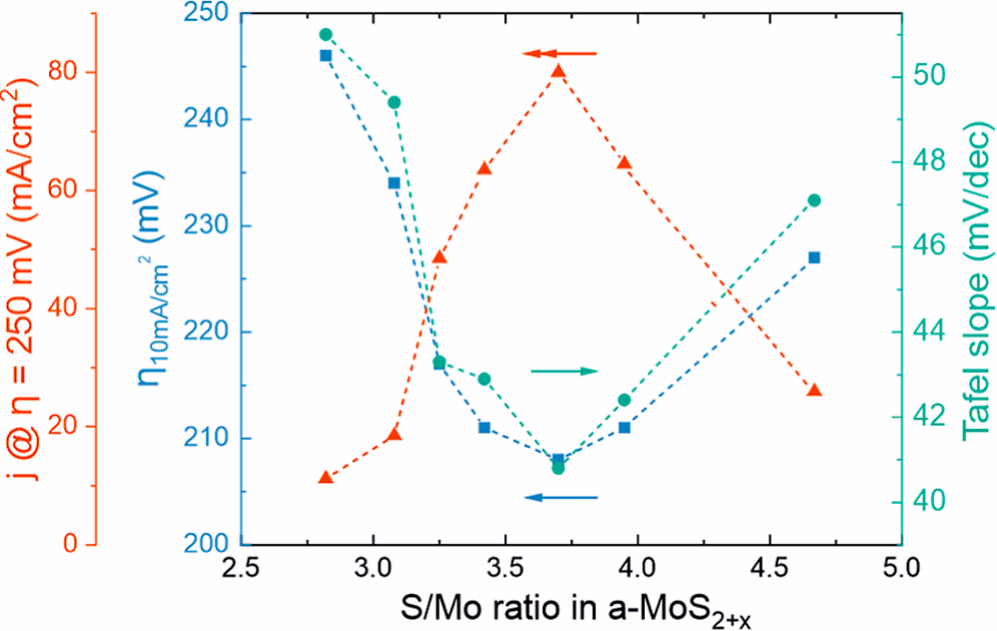
Effect of stoichiometry on HER activity of 7 nm a-MoS_*x*_ films as shown by overpotential required to reach a current density of 10 mA/cm^2^ (left), current density achieved at an overpotential of 250 mV (far left), and Tafel slope (right). The values were determined from the forward trace of the 5th CV scan after 100% *iR* drop compensation.

Seven of the 17 deposition conditions produced amorphous films with S/Mo ratios varying from 2.8 to 4.7. All the a-MoS_2+*x*_ samples proved to be highly active HER electrocatalysts in 0.5 M H_2_SO_4_ as shown in Figure 2a (see also Figures S5 and S6 in the Supporting Information). The HER activity was quantified by determining the overpotential required to reach a current density of 10 mA/cm^2^ (normalized to the geometric area), denoted as η_10mA/cm2_. As the thermodynamic potential for HER is 0 V versus reversible hydrogen electrode (RHE), η_10mA/cm2_ is the absolute value of the (negative) potential on the RHE scale where 10 mA/cm^2^ is reached. For a-MoS_2+*x*_, η_10mA/cm2_ ranged from 208 to 246 mV (Figure 2b). The Tafel slopes were low and rather similar to each other, 41–51 mV/dec (Figure 2c; Tafel plots in Figure S7 in the Supporting Information). These Tafel slopes suggest the Volmer–Heyrovsky mechanism with the Heyrovsky step as the rate-determining step for a-MoS_2+*x*_.^15^ The highest HER activity was observed at S/Mo ratios of 3.3–3.9, whereas the activity decreased at both higher and lower S/Mo ratios (Figure 3). The difference in overpotential between the most (a-MoS_3.7_) and the least (a-MoS_2.8_) active films was ∼40 mV, corresponding to an ∼8-fold difference in current density at η = 250 mV. As all the films had a similar catalyst loading, these activity differences are attributed to the effects of the S/Mo ratio on the film structure (Section 2.4).

The ten (at least partially) crystalline MoS_2_ films exhibited lower HER activity than a-MoS_2+*x*_, as shown by the higher η_10mA/cm2_ (298–520 mV) and Tafel slope (67–162 mV/dec) values (Figure 2). The most active c-MoS_2_ film produced an approximately five times lower current density (at η = 250 mV) compared to the least active a-MoS_2+*x*_ film, and the least active c-MoS_2_ films were ∼100 times less active than typical a-MoS_2+*x*_ films at this overpotential (Figures S6 and S7 in the Supporting Information). At higher overpotentials, the difference was even larger owing to the higher Tafel slopes of the c-MoS_2_ catalysts compared to those of a-MoS_2+*x*_. The most active c-MoS_2_ catalysts were found near the “border” of the deposition conditions producing amorphous films. Thus, these most active crystalline films are likely to be the most disordered/defective, as confirmed by Raman spectroscopy (Figure S8 in the Supporting Information). XPS showed that the c-MoS_2_ films deposited at 250 °C and below contained some S_2_^2–^ species found in a-MoS_2+*x*_ as well as a higher degree of disorder (Figures S12 and S13 in the Supporting Information). The films deposited at the highest temperatures were the most crystalline and roughest according to Raman spectroscopy and AFM (Figures S8 and S11 in the Supporting Information). Various defects^21,59−61^ including crystallite edges,^12^ S vacancies,^62−64^ and undercoordinated Mo atoms^63^ have been shown to increase the HER activity of c-MoS_2_. As increasing the deposition temperature decreased the HER activity, we believe that increased crystallinity had a large negative effect on HER activity that was not overcome by the simultaneously increased roughness and surface area (see Table S5 and Figure S15 for overpotentials calculated using specific surface area). Increasing the H_2_ flow ratio decreased the XPS S/Mo ratio, which has been linked to the formation of S vacancies in the literature.^62,63^ Increasing the H_2_ flow ratio also increased the electrical conductivity of the films due to hydrogen doping (Table S5 in the Supporting Information and ref (49)). Both S vacancies and electrical conductivity have been reported to be beneficial for HER.^62−64^ Because increasing the H_2_ flow ratio decreased the HER activity despite increasing conductivity and S vacancies, we hypothesize that hydrogen doping has a negative effect on the HER activity of c-MoS_2_. Density functional theory (DFT) studies suggest that H may bond in different ways in MoS_2_ with varying effects on the electrical properties (see the discussion in ref (49)). Understanding the bonding of H and its effects on HER activity requires additional studies. In addition, the PEALD process can be modified to prevent incorporation of detrimental H impurities.^51^

The activities of our a-MoS_2+*x*_ catalysts (η_10mA/cm2_ = 208–246 mV) are comparable to those reported for a-MoS_2+*x*_ electrocatalysts on flat substrates, despite our catalyst loadings being significantly lower compared to the majority of the literature (Table S8 in the Supporting Information).^26,27,33−36,42,43,65−70^ Although a few reports of a-MoS_2+*x*_ catalysts with η_10mA/cm2_ below 200 mV exist, these values have been achieved using loadings at least 50 times higher than those in the present study^7,8,29,30^ or high surface area substrates.^35,38,68^ As the MoS_*x*_ catalysts can be or become porous,^30,71^ high loadings can increase the activity per geometric area. The comparable or even superior inherent (per atom) activity of our a-MoS_2+*x*_ combined with the capability of PEALD to deposit conformal films on nanostructured substrates enables significant improvements in activity per geometric area toward practical electrolyzers.^44,47^ Compared to the thick film or particle form catalysts, our PEALD thin film catalysts lend themselves well to obtaining insights into catalytic activity and stability as described below. The activity of our c-MoS_2_ films (η_10mA/cm2_ = 298–520 mV) is in the range reported for nanocrystalline MoS_2_ thin films on flat substrates.^10,11,47^ Highly active c-MoS_2_ catalysts with η_10mA/cm2_ of 200–300 mV and lower have been obtained using, e.g., vacancy or strain engineering and strain.^13,14,17^ Compared to a-MoS_2+*x*_, a much wider variation in the HER activity of c-MoS_2_ is apparent both in our work and in the literature. Tuning of plasma chemistry, addition of plasma modification steps, and introduction of dopants can be used to further increase the HER activity of PEALD c-MoS_2_.

### 2.3 Changes in the Structure and Composition of MoS_*x*_ during HER

**Figure 4:**
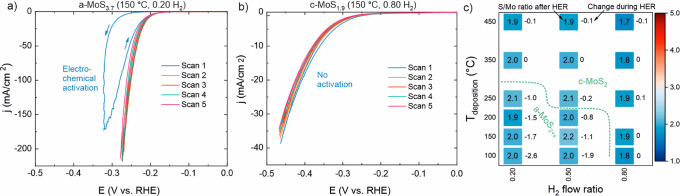
CV scans of (a) a-MoS_3.7_ (150 °C, 0.20 H_2_) and (b) c-MoS_1.9_ (150 °C, 0.80 H_2_) samples (7 nm, on GC, 100% *iR* drop compensation). (c) S/Mo stoichiometries of samples after HER and changes during HER as determined by XPS. The dashed green line indicates the border between amorphous and crystalline films.

For the a-MoS_2+*x*_ films, the first CV scan was clearly different from the following CVs, showing lower currents and strong hysteresis (Figure 4a—other samples are shown in Figure S6 in the Supporting Information). During the forward trace of the first scan, the current remained low until approximately −0.3 V vs RHE, after which an abrupt increase in current was observed. During the reverse trace, much higher currents were recorded compared to the forward trace. During the subsequent CVs, little to no further change occurred, suggesting that an electrochemical activation process took place during the first CV scan. A correlation between the catalyst S/Mo ratio and the characteristics of the activation process was observed; higher S/Mo ratios led to stronger hysteresis in the first CV, suggesting that the catalysts with higher S/Mo ratios had lower activity before activation. However, the activation potential (approximately −0.3 V vs RHE) and the duration of the process (∼1 CV) were independent of the initial stoichiometry. In contrast, for the c-MoS_2_ samples, the first five CVs practically overlapped as shown in Figure 4b, indicating that no activation occurred. The small decrease in the current during subsequent scans was attributed to accumulation of H_2_ bubbles that blocked a portion of the catalyst surface.

XPS measurements comparing the samples as-deposited and after five CVs revealed that the a-MoS_2+*x*_ samples lost a large portion of sulfur during the CVs (Figure 4c—the spectra will be discussed below). Strikingly, the amorphous samples with initial S/Mo ratios of 2.8–4.7 all had an identical MoS_2.0±0.1_ stoichiometry after the HER experiments. The decrease in the S/Mo ratio is linked to the electrochemical activation process. For clarity, we identify the samples throughout this study using their initial stoichiometry. In contrast, the crystalline samples showed practically no change in stoichiometry during HER. Furthermore, no changes in crystallinity, morphology, or chemical bonding (XPS) during HER were observed for the c-MoS_2_ catalysts, suggesting them to be stable under HER conditions (Figures S8, S9, and S12–14 in the Supporting Information).

The a-MoS_2+*x*_ catalysts remained amorphous despite the final S/Mo ratio of 2 (Figures S8 and S10). Raman spectroscopy showed a drastic decrease in the intensity of the Mo–Mo, Mo–S, and S–S modes during the electrochemical activation. Attempted operando Raman measurements were unsuccessful because of severe laser damage to the catalyst in the electrolyte regardless of the laser wavelength used (532, 633, and 785 nm). AFM and SEM revealed no changes in the smooth morphology of the a-MoS_2+*x*_ films upon electrochemical activation (Figure S9 in the Supporting Information). Transmission electron microscopy (TEM) of the a-MoS_4.5_ catalyst confirmed that a smooth morphology was retained after activation and that the activation proceeded throughout the film (Figure S10 in the Supporting Information). TEM combined with ellipsometry suggested that the film shrank from ∼7 to ∼4–5 nm during activation. Although no clear porosity was observed by TEM or AFM, we cannot rule out the possible formation of nanometer-scale pores during the activation of a-MoS_2+*x*_ where ∼20–50% of the constituent atoms are lost. The rather large amount of residual electrolyte remaining on or in the catalysts after HER may indicate porosity (vide infra).

**Figure 5:**
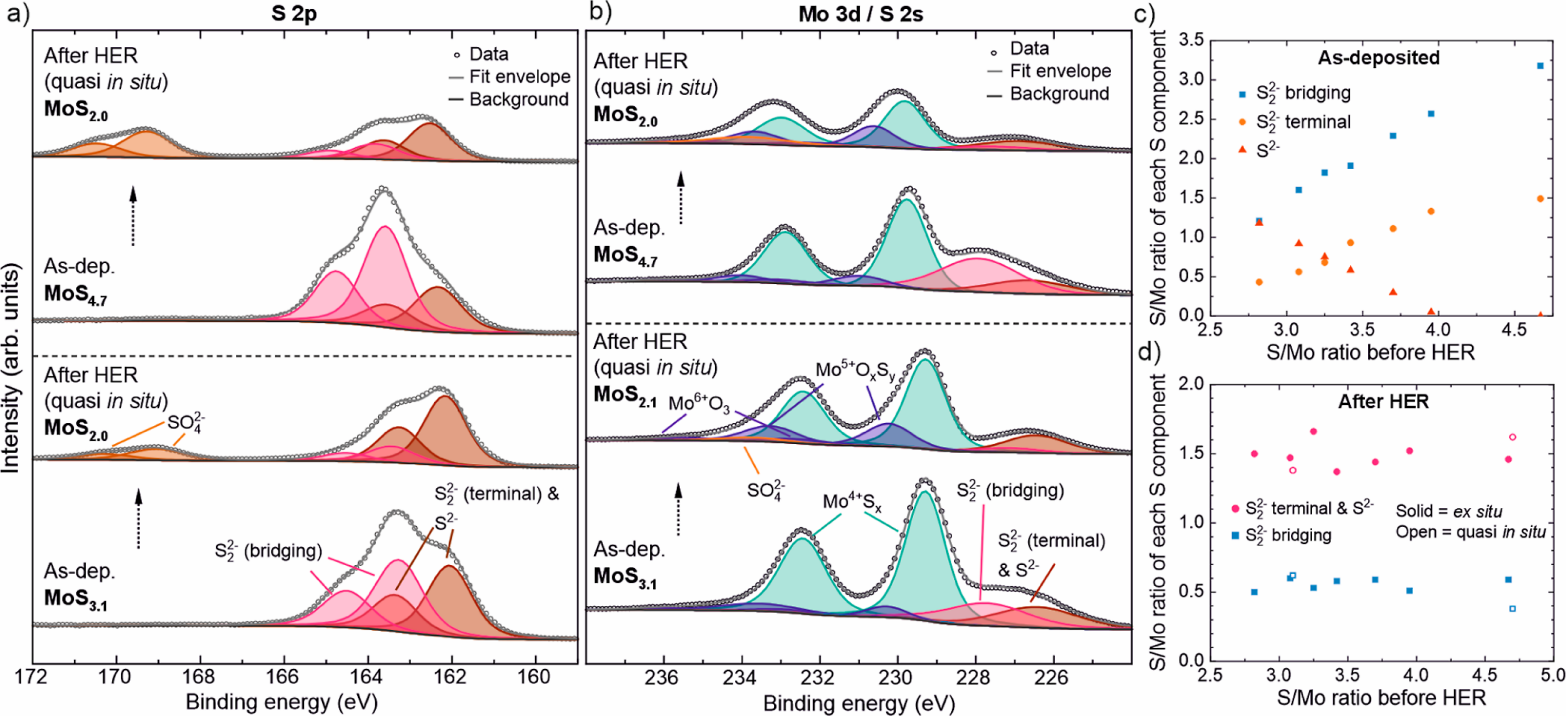
X-ray photoelectron spectra of (a) S 2p and (b) Mo 3d/S 2s regions of two a-MoS_2+*x*_ catalysts of different initial stoichiometries as-deposited and after HER (the latter was measured with quasi in situ XPS). (c) Concentration of three S components in as-deposited samples measured by ex situ XPS (plotted as S/Mo ratio). The lower BE component is attributed to terminal S_2_^2–^ and S^2–^ based on the oxidation states and charge neutrality assuming all S-bound Mo to be +4. (d) Concentration of S components after HER measured by both ex situ and quasi in situ XPS. The lower BE component could not be split into individual S species because of the significant amount of O bound to Mo.

XPS also provides insight into the structure of the catalysts beyond their stoichiometry. a-MoS_2+*x*_ contains S in different chemical environments with similar or even overlapping binding energies (BEs). Following the literature, we fit the broad S 2p peak using two doublets.^5,52^ The S 2p spectra are shown for a-MoS_4.7_ and a-MoS_3.1_ films in Figure 5a, while the fitted data for other films are presented in Table S4 and Figures S12–14 in the Supporting Information. The lower BE doublet (S 2p_3/2_ BE = 162.1 – 162.3 eV) is attributed to S^2–^ and terminal S_2_^2–^ species. The second doublet at 1.3 ± 0.1 eV higher BE is attributed to bridging S_2_^2–^ species. Other S species may also be present, of which apical S^2–^ would overlap with the bridging S_2_^2–^, and polysulfides would be observed at ∼1 eV higher BEs than our higher BE doublet. However, these are likely to be minor components as discussed in Section S8 in the Supporting Information. The relatively large full width at half-maximum (fwhm) of each S component (1.2–1.4 eV) indicates the heterogeneity of S coordination in a-MoS_2+*x*_ due to the presence of multiple S species and an amorphous structure. Our attempts to fit the S 2p peak using a larger number of S components were unsuccessful. Regardless, using knowledge of the oxidation state of Mo, which was found to be mostly 4+ when bound to sulfur (vide infra), the contributions of S^2–^ and terminal S_2_^2–^ species to the lower BE sulfur component can be estimated. As shown in Figure 5c, the amount of S^2–^ relative to Mo and other S species decreased with increasing S/Mo ratio from a major component (over 40% of S in MoS_2.8_) to negligible at S/Mo ratios above 4. Simultaneously, the fraction of both bridging and terminal S_2_^2–^ species increased, while the bridging S_2_^2–^ species always outnumbered the terminal species by a factor of 2–3. For comparison, most of the c-MoS_2_ films only showed the lower BE component attributed to S^2–^ with a smaller fwhm of 0.8–1.0 eV (Table S4 in the Supporting Information—a low-intensity higher BE S doublet was observed for defective c-MoS_2_ films).^5,30^

Conventional ex situ XPS measurements involve exposure of the samples to ambient air between the HER and XPS measurements, which can modify the species formed during the electrochemical activation process and under HER conditions. Therefore, we also performed quasi in situ XPS measurements of two a-MoS_2+*x*_ catalysts (a-MoS_4.7_ and a-MoS_3.1_) using a gastight electrochemical cell connected to an X-ray photoelectron spectrometer via vacuum transfer lines. These samples were chosen to cover a wide range of S/Mo ratios and thus to probe the effect of S/Mo ratio on catalyst structure. These measurements confirmed that both a-MoS_3.1_ and a-MoS_4.7_ samples lost S and ended up with a similar S/Mo ratio of 2 after HER. These results agree with the ex situ XPS measurements of all a-MoS_2+*x*_ samples shown in Figure 5d (SO_4_^2–^ contribution at S 2p_3/2_ BE ≈ 169 eV resulting from the residual electrolyte was excluded). The majority of the S loss occurred in the higher BE component attributed to bridging S_2_^2–^. Regardless, approximately 20–30% of the S remaining after HER (corresponding to S/Mo ≈ 0.4–0.6) was attributed to bridging S_2_^2–^. The absolute amount of S contributing to the lower BE doublet (terminal S_2_^2–^ and S^2–^) was practically unchanged during HER (corresponding to S/Mo ≈ 1.5). However, changes in the relative amounts of terminal S_2_^2–^ and S^2–^, namely, a shift toward S^2–^, may occur during HER. Such a (terminal or bridging) S_2_^2–^ to S^2–^ transformation has been reported under reducing HER conditions.^32^ The appearance of Mo^5+^O_*x*_S_*y*_ species of unknown stoichiometry (see below) prevents quantitative assessment of the terminal S_2_^2–^ and S^2–^ species after HER.

The Mo 3d/S 2s region was fit using three Mo 3d doublets (Mo^4+^S_*x*_, Mo^5+^O_*x*_S_*y*_, and Mo^6+^O_3_) and three S 2s singlets (BEs from the literature^72,73^ and intensities from the S 2p spectra). Mo^4+^–S was expectedly the main component (90 ± 5%) in all the as-deposited a-MoS_2+*x*_ samples (Figures 5b and S17 in the Supporting Information).^5,74^ The Mo 3d_5/2_ BEs of the Mo^4+^–S component increased slightly with increasing S/Mo ratio from 229.2 to 229.8 eV (Figure S18a in the Supporting Information). A small amount (3–6% of Mo) of Mo^6+^ species (Mo 3d_5/2_ BE = 232.5–233.0 eV)^72^ is attributed to air exposure between sample preparation and XPS measurements (a few hours). A third doublet with a Mo 3d_5/2_ BE of 1.0 ± 0.1 eV above the Mo^4+^ component is attributed to Mo^5+^ (oxy)sulfide, an oxidation intermediate, although some authors have suggested the presence of Mo^5+^ in a-MoS_2+*x*_ (Section S8 in the Supporting Information). The BE difference between the Mo^4+^ and Mo^5+^ species is in agreement with the studies on MoO_*x*_,^75^ although other studies have suggested a larger difference in BEs between Mo^4+^S_*x*_ and Mo^5+^O_*x*_S_*y*_ of ∼2.0 eV.^76^ Recently, Kendall et al.^77^ identified a component ∼0.8 eV above Mo^4+^–S_*x*_ yet ∼0.8 eV below a feature they assigned to Mo^5+^–O_*x*_S_*y*_. Although the precise identity of this feature we denote Mo^5+^–O_*x*_S_*y*_ is unknown, it is suggested to be related to HER activity at least indirectly (vide infra) as Kendall et al.^77^ also suggested. Our efforts to fit an additional Mo component similar to Kendall et al. were unsuccessful. Integration of the O 1s spectrum followed by deduction of the electrolyte (sulfate) contribution confirmed that O was incorporated into the catalyst during activation. Quantification of incorporated O was considered unreliable and was not pursued because of the rather large amount of sulfate present.

After HER, no major changes in the Mo^6+^ species were observed in either of the samples measured by quasi in situ XPS (Figure 5b). This shows that the Mo^6+^ species were not reduced under HER conditions and that the quasi in situ measurement successfully prevented oxidation of the film between the HER and XPS measurements. In ex situ measurements, an increase in Mo^6+^ species after HER was observed for most of the a-MoS_2+*x*_ samples (Figure S17 in the Supporting Information). Thus, we used the quasi in situ Mo 3d spectra to decipher the structure of the activated catalyst. The intensity of the Mo^5+^O_*x*_S_*y*_ doublet increased during HER according to quasi in situ XPS, reaching ∼20–30% of the total Mo content. For the most S-rich samples, this change in the Mo 3d spectrum could also have been fit as a broadening of Mo^4+^–S, whereas for the less S-rich a-MoS_2+*x*_ films, a clear shoulder was formed. This effect of the initial S/Mo ratio may be attributed to the lower Mo^4+^ BE of the samples with lower initial S/Mo ratios, while the BE of the Mo^5+^ component (230.5 ± 0.1 eV) after HER was independent of the starting stoichiometry. These differences in the Mo 3d spectra after HER suggest that the starting stoichiometry affects the structure of the electrochemically activated catalyst as discussed in the following section.

At first, the oxidation of Mo under reducing HER conditions and exclusion of air seems puzzling, yet there are several plausible explanations. The loss of S, primarily from S_2_^2–^ which needs to be reduced to be released as H_2_S as observed by Xi et al.,^29^ itself requires a balancing oxidation reaction, potentially involving the oxidation of Mo^4+^. In addition, the S loss process generates unsaturated Mo sites, where H^+^ may adsorb reductively and cause Mo to oxidize as suggested by Tran et al.^31^ The oxidation of Mo and the potential role of oxygen are further discussed below.

### 2.4 What Makes a-MoS_*x*_ Active for HER?

A large variety of species have been suggested to be responsible for the high HER activity of a-MoS_*x*_, including bridging S_2_^2–^ (refs (27, 30, 34, and 35)), terminal S_2_^2–^ (refs (78 and 79)), S^2–^ (refs (27, 32, and 66)), Mo^5+^ (refs (30, 32, and 77)), Mo^3+^ (ref (78)), and unsaturated Mo sites.^29,36^ To understand these suggestions, we first need to consider the possible structures of the a-MoS_*x*_ catalysts in the as-deposited state and after electrochemical activation (see Section S8 of the Supporting Information). Our XPS assignments are based on the cluster model introduced by Weber et al.^5^ with modifications proposed by others.^29−31,77^ Alternative chain models have also been proposed;^80,81^ however, we are not aware of a chain model including S^2–^ and both terminal and bridging S_2_^2–^ species. The stoichiometry,^29,31^ and likely the deposition method and conditions, affect the structure of a-MoS_2+*x*_. For example, solution methods utilize precursors such as (NH_4_)_2_[MoS_4_] which already contain Mo–S complexes that form the basis for the resulting a-MoS_*x*_ structure,^5,8,66^ whereas methods such as PEALD and sputtering^29^ supply Mo and S from separate sources.

During HER, electrochemical activation of a-MoS_2+*x*_ HER catalysts together with partial loss of sulfur has been observed in several studies with typical final S/Mo ratios ranging from 1.6 to 2.0.^29,33,37,69^ Electrodeposited^27,33^ and ALD^42^ a-MoS_1.7–2.0_ catalysts were reported not to undergo compositional changes, which can be taken as support for a-MoS_∼2.0_(O_*x*_) as an active HER catalyst or at least a composition stable under HER. Our observation of electrochemical activation resulting in a-MoS_2.0_ films irrespective of the starting stoichiometry (MoS_2.8–4.7_) supports this view. The structural changes that occur during electrochemical activation and the structure of the activated HER catalyst should be resolved to understand the HER activity of a-MoS_2+*x*_. Using quasi in situ and ex situ XPS, we attributed the sulfur loss occurring during activation mostly to bridging S_2_^2–^, especially for high initial S/Mo ratios, while terminal S_2_^2–^ species are also likely to partially disappear. The lost S can be released as H_2_S as observed by Xi et al.^29^ Some of the lost S_2_^2–^ species may also be reduced to S^2–^.^82^ Literature suggests that the starting stoichiometry and deposition method may also affect the activated structure. For example, operando X-ray absorption studies by Lassalle-Kaiser et al.^78^ observed Mo–Mo bonding to disappear during HER in their electrodeposited a-MoS_2.9_ catalysts, while Wu et al.^69^ observed short Mo–Mo bonds, a characteristic of both cluster and chain models of a-MoS_*x*_, to remain after the electrochemical activation of PEALD a-MoS_2.3_. Xi et al.^29^ observed sputtered a-MoS_3.8_ to form c-MoS_2_, whereas we did not observe crystallization.

The formed catalyst structure is often used to propose the potential catalytic sites. Considering the agreement between the stoichiometry of activated a-MoS_*x*_ (*x* ≈ 2) and the often similar activity (Table S8), the proposed variety of active sites listed above is striking. In addition to the discussed effects of the deposition method and stoichiometry, this reflects the difficulty in directly observing catalytic sites, even when using operando spectroscopic methods. Likely, the most direct evidence comes from operando Raman studies of electrodeposited a-MoS_3.1_ and a-MoS_1.9_ by Deng et al.,^27^ who observed the formation of S–H species that were assigned as catalytic intermediates. This interpretation is consistent with Tafel analysis suggesting the Heyrovsky step as the rate-determining step. Based on the DFT calculations of Deng et al.,^27^ the S^2–^ species formed from bridging S_2_^2–^ were proposed as the HER active site. While the direct detection of S–H and literature on c-MoS_2_ suggest that S sites are the likely active sites in a-MoS_*x*_, Mo will also play a role by affecting the electronic structure of the material. Our quasi in situ XPS measurements did not find evidence for the proposed Mo^3+^ (ref (78)) sites, while we observed oxidation of a fraction of the initial Mo^4+^ species in line with the suggestion that Mo^5+^–O (refs (37 and 77)) species are important for HER. In the previous studies speculating on Mo^5+^ active sites, a significant Mo^5+^ and O content already resulted from the material synthesis. In contrast, our quasi in situ XPS results show that Mo^5+^ formation occurs during electrochemical activation. Considering S^2–^ species as the active site^27^ (compatible with our XPS results and c-MoS_2_ active sites^12^), the different Mo sites, including Mo^4+^–S and Mo^5+^–O as well as differences in Mo^4+^–S species observed by quasi in situ XPS, are proposed to modulate the activity of the S^2–^ sites. We hypothesize that such differences in activity-modulating Mo sites may at least partially explain the effect of the initial stoichiometry on the HER activity.

**Figure 6:**
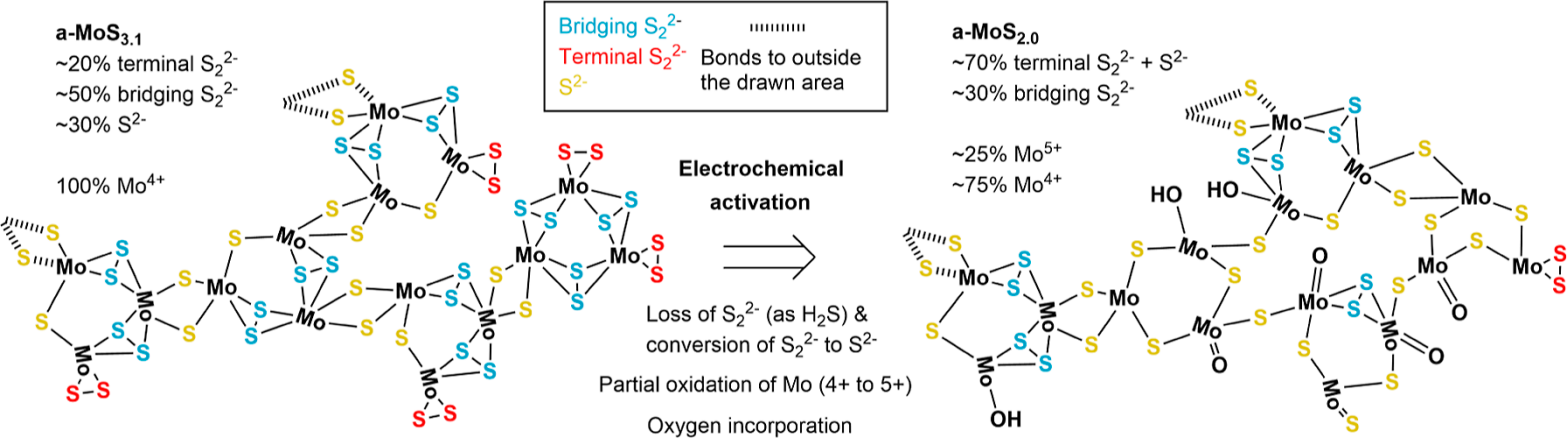
Illustration of the a-MoS_3.1_ catalyst as-deposited and after electrochemical activation and a summary of changes occurring during activation based on the cluster model, our XPS data, and the literature discussed in Section 2.4.

Figure 6 illustrates the species that may be present in our a-MoS_3.1_ catalyst based on our XPS measurements and the literature discussed above and in Section S8 of the Supporting Information. In as-deposited a-MoS_3.1_, approximately half of the sulfur is present as bridging S_2_^2–^ species within Mo_3_S_*x*_ clusters, while the other half is divided into terminal S_2_^2–^ and S^2–^ species. Varying the amount of different S species can accommodate a wide range of S/Mo ratios as illustrated in Figure S20 in the Supporting Information. Mo appears to be present solely as Mo^4+^ in the as-deposited catalysts. During electrochemical activation, S is lost until MoS_2_ stoichiometry is reached, and a change in the relative abundance of the S species also occurs. The loss of S and the accompanying breaking of bonds have a major effect on the catalyst structure and likely lead to the incorporation of oxygen together with the oxidation of ∼25% of Mo^4+^ to Mo^5+^. The activated catalyst is also unstable in air and can oxidize further. For more S-rich catalysts, a larger fraction of the initial S is lost, which leads to larger changes from the as-deposited to the activated structure. In addition to the type of Mo species discussed above, the degree of S loss can also affect, among others, the electrical conductivity, surface area, and structural stability of the catalyst. These factors also influence the HER performance.

### 2.5 Stability of a-MoS_2+*x*_ Catalysts

**Figure 7:**
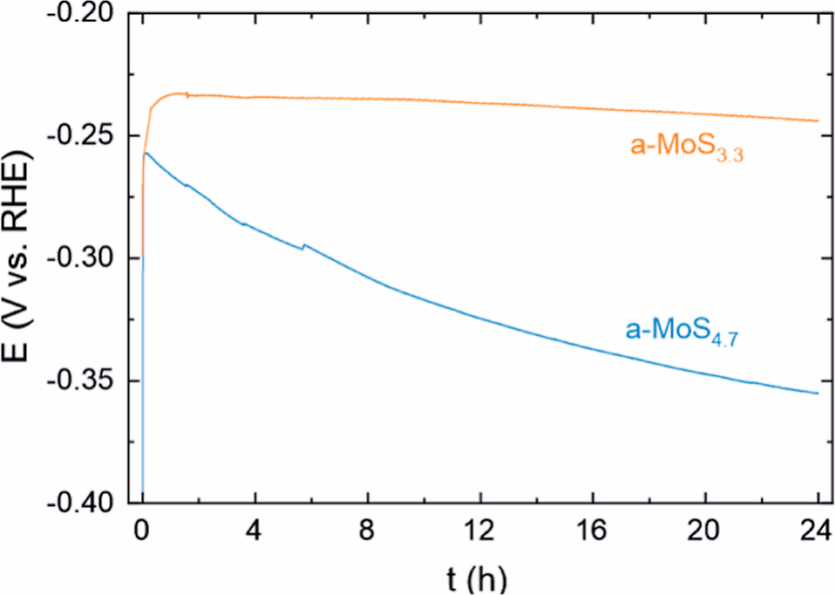
Stability of a-MoS_2+*x*_ catalysts of different initial stoichiometries for 24 h at a constant current density of 10 mA/cm^2^. Here, 15 nm films deposited on carbon fiber paper at 100 °C and H_2_ flow ratio of 0.20 (a-MoS_4.7_) and 150 °C and H_2_ flow ratio of 0.50 (a-MoS_3.3_) were used.

Although the high HER activity of a-MoS_*x*_ is well established in the literature, there are varying reports regarding its stability (Table S8 in the Supporting Information). We studied the effect of the initial stoichiometry on stability using catalysts with the highest (a-MoS_4.7_) and moderate (a-MoS_3.3_) S/Mo ratios. Activation of both catalysts occurred under galvanostatic conditions, showing that performing CVs is not necessary as long as a negative enough potential (approximately −0.3 V vs RHE) is reached. The a-MoS_3.3_ catalyst showed good stability with only a 10 mV increase in overpotential during a 24 h galvanostatic measurement at 10 mA/cm^2^ (Figure 7). Previously, a PEALD a-MoS_2.3_ film has also been found to possess good stability.^69^ In contrast, the a-MoS_4.7_ catalyst required a 100 mV increase in applied potential during the 24 h measurement. Therefore, the initial stoichiometry of the a-MoS_2+*x*_ catalyst affects both the stability and activity. A higher initial stoichiometry leads to lower stability, which may be due to the larger amount of S lost during activation and, consequently, larger changes in the catalyst structure.

## 3 Conclusions

We have shown how the control of plasma chemistry and deposition temperature enables PEALD to be used to prepare thin films of both amorphous MoS_*x*_ with an adjustable S/Mo ratio (2.8–4.7) and crystalline MoS_2_ with tailored characteristics including crystallinity, morphology, and electrical conductivity. To obtain new insights into the factors that affect the activity of MoS_*x*_ as HER electrocatalyst, we prepared 14 different MoS_*x*_ films with comparable loading yet different properties and characterized them thoroughly before and after HER. All amorphous MoS_*x*_ films were found to be highly active with low overpotentials of 210–250 mV at 10 mA/cm^2^ (in 0.5 M H_2_SO_4_). The highest activity was observed at a S/Mo ratio of 3.7. The amorphous films (S/Mo = 2.8 – 4.7) underwent a rapid electrochemical activation process at approximately −0.3 V vs RHE, where S was partially lost until amorphous MoS_2_ with incorporated oxygen was formed. The catalyst structure was investigated using quasi in situ XPS, which showed the formation of S^2–^ and Mo^5+^–S/O species under HER conditions. We propose that the formed S^2–^ species may act as active HER sites with neighboring Mo atoms modulating their activity. The initial a-MoS_*x*_ stoichiometry affects chemical environment (binding energy) of the activity-modulating Mo^4+^–S_*x*_ species and consequently the catalyst activity and stability under HER conditions. Thus, tailoring the stoichiometry of a-MoS_*x*_ is crucial for practical electrolyzer applications. In addition to stoichiometry control, PEALD enables deposition on high surface area supports to obtain large current densities. Our structural insights can be used as input for dedicated operando spectroscopy and DFT studies into the precise structure of the active site. For crystalline MoS_2_, we observed no structural or compositional changes during HER. The HER overpotentials of the c-MoS_2_ films ranged from 300 to 520 mV at 10 mA/cm^2^, which was linked to film structure, in particular crystallinity. The lowest overpotentials were found for the most defective c-MoS_2_ films. Both increased crystallinity and H doping are proposed to decrease HER activity, overshadowing the effects of electrical conductivity for our catalysts. Although less active than a-MoS_*x*_, the lack of structural changes of c-MoS_2_ during HER is notable and potentially beneficial in certain cases. PEALD also offers avenues to further enhance the activity of c-MoS_2_ by doping (or avoiding it) and controlling the generation of defects via plasma. Our process also enables creating more complex catalyst architectures, such as by combining a-MoS_*x*_ and c-MoS_2_ layers on nanostructured supports, or by incorporating a controlled amount of oxygen into the catalysts.

## 4 Experimental Section

### 4.1 Film Deposition

Thin films of MoS_*x*_ were deposited by PEALD using Mo(N^t^Bu)_2_(NMe_2_)_2_ and mixed H_2_S/H_2_/Ar plasma as precursors. The depositions were performed in an Oxford Instruments FlexAL PEALD reactor equipped with a remote inductively coupled plasma (ICP) source operated at 13.56 MHz. Mo(N^t^Bu)_2_(NMe_2_)_2_ (98%, Strem Chemicals) was heated to 50 °C in an external canister and supplied to the chamber by an Ar flow. Ar was also used as a purge gas. The flow rates of H_2_S (99.5%), H_2_ (99.999%), and Ar (99.999%) gases supplied by Linde gas were controlled by mass flow controllers.

The MoS_*x*_ PEALD process was initially developed by Sharma et al.^46^ using a fixed plasma gas composition. Mattinen et al.^49^ modified the process by altering the gas flows, in particular H_2_ flow ratio, to control deposition and film properties. In the present article, H_2_ flow ratios of 0.20, 0.50, and 0.80 were used (Table 1). Table temperature, i.e., the deposition temperature, was varied between 100 and 450 °C. The actual substrate temperature is lower especially at the highest temperatures due to the reactor wall temperature of ≤150 °C and limited thermal contact between the heated table and the substrate.^83^ The ALD cycle used is illustrated in Figure S1 in the Supporting Information. For more details of the ALD process and experimental details, see ref (49).

**Table 1:** Gas Flows through the ICP Tube for Different H_2_ Flow Ratios^a^

H_2_ flow ratio H_2_/(H_2_ + H_2_S)	H_2_S flow rate (sccm)	H_2_ flow rate (sccm)	Ar flow rate (sccm)
0.20	8	2	40
0.50	5	5	40
0.80	2	8	40

a In each case, the total gas flow was 50 sccm.

Films for electrochemical experiments were deposited on glassy carbon substrates measuring 2.2 × 2.2 × 0.3 cm (Sigradur G from HTW Hochtemperatur-Werkstoffe GmbH, Germany). Prior to deposition, the substrates were polished to a mirror finish using an aqueous slurry of 50 nm Al_2_O_3_ particles (BASi) applied to a velvet polishing pad (BASi), followed by rinsing with deionized H_2_O. Typical root-mean-square roughness of polished substrates was 1–2 nm as measured by AFM. The surface area was practically identical (100–101%) to its geometric area. For HER stability studies, films were also deposited on carbon fiber paper substrates measuring approximately 1 × 1 × 0.04 cm (Spectracarb 2050A from Giner ELX Inc., USA). Additionally, silicon (100) substrates with a 450 nm wet thermal SiO_2_ layer (Siegert Wafer) were used as reference substrates. Unless otherwise noted, the number of ALD cycles was adjusted to reach a thickness of approximately 7 nm based on in situ SE measurements on SiO_2_/Si substrates (see ref (49) for more details). In practice, this translated to 57–111 ALD cycles (Table S1 in the Supporting Information).

### 4.2 Electrochemical Measurements

HER activity measurements were performed in a glass cell (Figure S2a in the Supporting Information) using a three-electrode setup controlled by a potentiostat (Autolab PGSTAT302N, Metrohm). A commercial Ag/AgCl (sat. KCl) electrode was used as the reference electrode and brought close to the working electrode (∼0.5 cm) using a Luggin capillary. Measured potentials were converted to RHE scale (245 mV more positive compared to Ag/AgCl at pH = 0.3). A glassy carbon rod was used as the counter electrode and placed in a compartment separated by a glass frit. The MoS_*x*_ films deposited on GC plates measuring 2.2 × 2.2 × 0.3 cm were used as the working electrodes. The plates were mounted face down into a custom-made polyether ether ketone sample holder exposing a sample area of 3.1 cm^2^, which was then mounted onto a rotating disk electrode apparatus (Metrohm). The 0.5 M H_2_SO_4_ (pH = 0.3) electrolyte was prepared from concentrated H_2_SO_4_ (99.99% purity or ACS reagent grade, Sigma-Aldrich) and ultrapure water (resistivity 18 MΩ cm). The electrolyte was bubbled with Ar or N_2_ for at least 15 min prior to measurements. During measurements, the gas flow was switched to purge the headspace above the electrolyte.

For a typical HER measurement, five CV scans between −0.2 and −0.75 V (vs Ag/AgCl) were applied at a scan rate of 10 mV/s, while the electrode was rotated at 1600 rpm. The reported overpotentials and Tafel slopes were determined from the fifth CV cycle after 100% *iR* drop postcompensation. The resistance *R*_u_ was determined using electrochemical impedance spectroscopy (0.2 V vs Ag/AgCl, 10 Hz to 100 kHz, 10 mV root-mean-square AC amplitude, no rotation). The data point with the phase closest to 0° (typically at 50–100 kHz) was taken as the *R*_u_ (0.4–0.8 Ω).

To probe the repeatability of PEALD and electrochemical measurement, an identical sample (a-MoS_3.1_ deposited at 250 °C, H_2_ flow ratio 0.20, 7 nm thickness) was measured during each day of electrochemical measurements, yielding an average ± standard deviation of 239 ± 11 mV for η_10mA/cm2_ and 52 ± 1 mV/dec for the Tafel slope.

The stability of a-MoS_3.3_ (150 °C, H_2_ flow ratio 0.50) and a-MoS_4.7_ (100 °C, H_2_ flow ratio 0.20) samples was probed by 24 h galvanostatic measurements at 10 mA/cm^2^. For these measurements, the catalyst was deposited on carbon fiber paper, which was connected to an inert Ta clip and submerged into the electrolyte vertically (Figure S2b in the Supporting Information). This substrate greatly facilitated bubble removal compared to the GC substrates held face down for activity measurements. For the stability measurements, a saturated calomel reference electrode (Hg/Hg_2_Cl_2_, CH instruments) was externally referenced to ferrocenecarboxylic acid in 0.2 M phosphate buffer titrated with NaOH to pH 7 (284 mV vs SCE).^84^ High-purity concentrated H_2_SO_4_ (Fischer Chemical Optima) and ultrapure water were used to prepare the 0.5 M H_2_SO_4_ electrolyte used for the stability experiments. Otherwise, the stability measurements were done in a setup analogous to that described above.

### 4.3 Ex Situ Characterization

The film morphology was examined by SEM (Zeiss Sigma) using an acceleration voltage of 3 kV and an InLens detector and by AFM (Bruker Dimension Icon) in the PeakForce Tapping based ScanAsyst mode in air. Probes with a nominal spring constant of 0.4 N/m and tip radius of 2 nm were used (Scanasyst-air, Bruker). Roughness was calculated as root-mean-square (*R*_q_) value after flattening the 500 × 500 nm image (1st order) using Bruker Nanoscope 2.0 software. For AFM measurements of as-deposited catalysts, films deposited on SiO_2_/Si were mostly used due to the sensitivity of the GC substrate morphology to the polishing process.

Film structure and crystallinity were evaluated by Raman spectroscopy using a confocal Raman microscope (Renishaw inVia) equipped with a 514 nm laser, 50x objective (NA 0.75), and 1800 lines/mm grating. Six spectra of 10 s each were accumulated during each measurement. Laser power was estimated at 0.6 mW on the sample (100 mW laser with 1% neutral density filter, estimated optical losses). No baseline subtraction or other spectral processing were performed.

Ex situ XPS measurements were performed using a Thermo Scientific K-Alpha spectrometer equipped with a monochromatic Al Kα source (*h*ν = 1486.6 eV) focused to a 400 μm diameter spot on the sample. Charging effects were minimized using an electron flood gun during measurements. The measured spectra were referenced to a binding energy of 284.8 eV for C 1s peak of adventitious carbon (C–H component). A pass energy of 50 eV was used. Peak fitting was performed using Avantage software (Thermo Scientific). Gaussian–Lorentzian (30% Lorentzian) sum functions were used to describe the individual components with a Shirley-type background. See Tables S2 and S3 in the Supporting Information for fitting constraints.

The cross-sectional TEM sample was prepared using a FEI Nova Nanolab 600 dual-beam focused-ion beam/SEM instrument following a standard lift-out procedure. The TEM imaging was performed using a probe-corrected JEOL ARM 200F instrument operated at 200 kV.

### 4.4 Quasi In Situ XPS

Quasi-in situ XPS was performed using a SPECS Phoibos NAP-150 electron analyzer. Core level spectra were acquired using a monochromatic Al Kα source (*h*ν = 1486.6 eV, SPECS XR-50) operated at 50 W. The pass energy was set to 40 eV with a step size of 0.1 eV and a dwell time of 0.5 s. No charge neutralization was used. Energy referencing and peak fitting were performed as described for the ex situ measurements above. The electrochemical measurements were performed in a gastight three-electrode electrochemical cell (SPECS IS-EC-AP). The electrochemical experiments prior to XPS were performed in 0.5 M H_2_SO_4_ using a-MoS_2+*x*_ films deposited on 1.2 × 1.2 × 0.05 cm GC substrates (Sigradur G from HTW Hochtemperatur-Werkstoffe GmbH, Germany) as a working electrode, a Pt wire as a counter electrode, and a commercial RHE (Hydroflex, Gaskatel GmbH, Germany) as a reference electrode. The electrolyte was N_2_ saturated 0.5 M H_2_SO_4_. During the measurement, the electrochemical cell was purged with N_2_ gas. Ten CV cycles between 0.1 and −0.5 V vs RHE at a scan rate of 10 mV/s were performed to electrochemically activate the a-MoS_2+*x*_ samples, after which the applied potential was held at −0.5 V vs RHE for 10 min. Then, the electrolyte was removed with a pump, and the working electrode was rinsed with N_2_-saturated H_2_O, blown dry with N_2_, and transferred to a buffer chamber without any contact with air. The buffer chamber was then pumped to reach ultrahigh-vacuum conditions (<10^–8^ mbar, UHV), after which the working electrode was further transferred to the analysis chamber to record XPS spectra under UHV conditions.

## Supporting Information Available

PEALD process schematic and parameters, photos of electrochemical cells, studies on the effect of catalyst thickness, full electrochemical data of 7 nm catalysts (CVs and Tafel plots), characterization of as-deposited and post-HER catalysts (XPS, SEM, Raman, AFM, and TEM), quasi in situ XPS data, literature on activity and active species, and structure of a-MoS_2+*x*_ catalysts (DOCX)
