# Supplementary material for: Structural Aspects of MoSx Prepared by Atomic Layer Deposition for Hydrogen Evolution Reaction
Source: ACS Catal. 2024 Jun 20;14(13):10089–101. doi: 10.1021/acscatal.4c01445 (PMC11232007; doi:10.1021/acscatal.4c01445)
Supplement: Supplementary file 1 — cs4c01445_si_001.docx [file cs4c01445_si_001.docx]

Supporting Information for:

**Structural Aspects of MoS_x_ Prepared by Atomic Layer Deposition for Hydrogen Evolution Reaction**

Miika Mattinen,^1†^ Wei Chen,^2^ Rebecca A. Dawley,^3^ Marcel A. Verheijen,^1,4^ Emiel J. M. Hensen,^2^ W. M. M. Kessels,^1^ Ageeth A. Bol^1,3*^

1 Department of Applied Physics and Science Education, Eindhoven University of Technology, PO Box 513, 5600 MB Eindhoven, The Netherlands

2 Department of Chemical Engineering and Chemistry, Eindhoven University of Technology, PO Box 513, 5600 MB Eindhoven, The Netherlands

3 Department of Chemistry, University of Michigan, 930 N. University Ave, Ann Arbor, MI, 48109-1055, United States of America

4 Eurofins Materials Science Netherlands, High Tech Campus 11, 5656 AE Eindhoven, The Netherlands

† Present address: Department of Chemistry, University of Helsinki, PO Box 55, 00014, Finland

*corresponding author: aabol@umich.edu, [a.a.bol@tue.nl](mailto:a.a.bol@tue.nl)

**Table of contents**

S1. Deposition conditions and schematic S3

**Figure S1**. Schematic of the ALD process used to deposit MoS_x_ S3

**Table S1**. Process conditions and number of ALD cycles applied to reach a target thickness of 7 nm S3

S2. Electrochemical cells S4

**Figure S2**. Photographs of the electrochemical cells used in this work S4

S3. MoS_x_ catalysts of different thicknesses S5

**Figure S3**. Electrochemical characterization of a-MoS_3.1_ films of different thicknesses S6

**Figure S4**. Electrochemical characterization of c-MoS_2_ films of different thicknesses S7

S4. Electrochemical data of all 7 nm MoS_x_ catalysts S8

**Figure S5**. Current density at an overpotential of 250 mV S8

**Figure S6**. Five CVs of 7 nm MoS_x_ catalysts prepared under different conditions. S9

**Figure S7**. Tafel plots of 7 nm MoS_x_ catalysts prepared under different conditions S10

S5. Characterization of MoS_x_ catalysts as-deposited and after HER S11

**Figure S8**. Raman spectra of MoS_x_ catalysts prepared under different conditions S11

**Figure S9**. AFM and SEM images of selected MoS_x_ catalysts as-deposited and after HER S12

**Figure S10**. Cross-sectional TEM images of an “a-MoS_4.7_” catalyst after HER S13

**Figure S11**. AFM images of MoS_x_ films deposited on SiO_2_/Si S14

**Figure S12**. X-ray photoelectron spectra of MoS_x_ films deposited at 100 and 150 °C before and after HER experiments S15

**Figure S13**. X-ray photoelectron spectra of MoS_x_ films deposited at 200 and 250 °C before and after HER experiments S16

**Figure S14**. X-ray photoelectron spectra of MoS_x_ films deposited at 350 and 450 °C before and after HER experiments S17

**Table S2**. XPS fitting constraints for the Mo 3d / S 2s region S18

**Table S3.** XPS fitting constraints for the S 2p region S18

**Table S4**. XPS peak fitting of MoS_x_ deposited at different conditions before and after HER by *ex situ* XPS S19

S6. Summary of physicochemical and electrochemical characterization S22

**Table S5**. Summary of physicochemical and electrochemical characterization of different films S22

**Figure S15**. Overpotential using specific surface area from AFM S22

S7. Quasi *in situ* XPS S23

**Table S6**. XPS peak fitting of MoS_x_ samples measured before (*ex situ* XPS) and after HER (quasi *in situ* XPS) S23

**Figure S16**. Comparison of a-MoS_x_ films measured with quasi *in situ* as well as *ex situ* XPS S24

**Figure S17**. Relative concentrations of Mo^4+^, Mo^5+^, and Mo^6+^ species in a-MoS_2+x_ as a function of initial S/Mo ratio S25

**Figure S18**. Mo 3d_5/2_ BE of Mo^4+^ and S 2p_3/2_ Bes of a-MoS_2+x_ catalysts as a function of initial S/Mo ratio S25

S8. Active species of a-MoS_2+x_ HER catalysts S26

**Figure S19**. Structures proposed for a-MoS_x_ and related bonding motifs and clusters S28

**Figure S20**. Illustration of bonding motifs in as-deposited a-MoS_2+x_ catalysts of different stoichiometries S29

**Table S7**. Summary of selected investigations looking into structure and active sites of a-MoS_x_ catalysts in acid S32

S9. Literature on HER activity of a-MoS_2+x_ catalysts S33

**Table S8**. Summary of literature on a-MoS_x_ HER catalysts and their activity and stability S33

S10. References S35

# S1. Deposition conditions and schematic


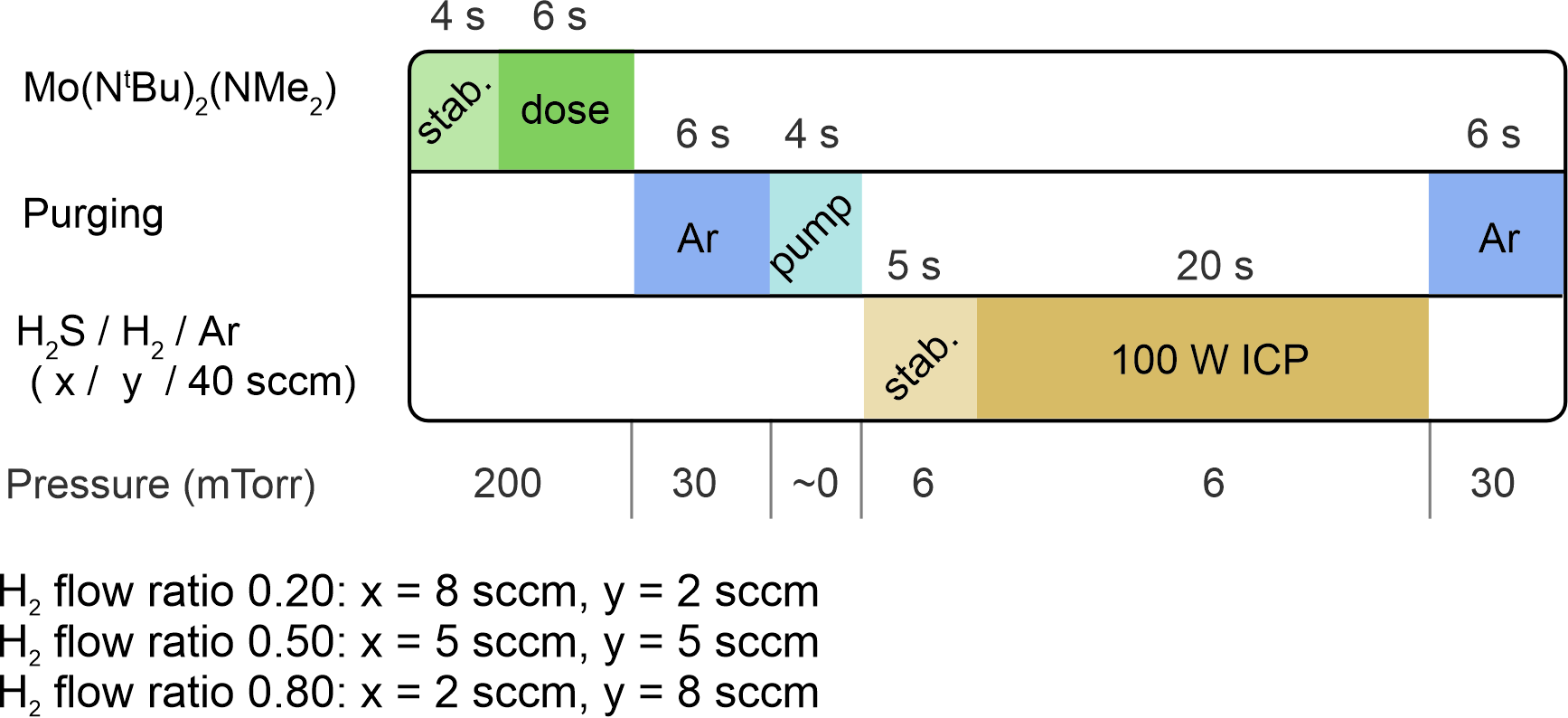


**Figure S1**. Schematic of the ALD process (one ALD cycle) used to deposit MoS_x_. The lighter shading refers to gas flow/pressure stabilization (stab.) steps. Purging is done both with Ar flown into the chamber (denoted Ar) and without gas flow (denoted pump).

**Table S1**. Process conditions and number of ALD cycles applied in each case to reach a target thickness of 7 nm.

| T_deposition_ (°C) | H_2_ flow ratio | Number of ALD cycles |
| --- | --- | --- |
| 100 | 0.20 | 65 |
|  | 0.50 | 57 |
|  | 0.80 | 57 |
| 150 | 0.20 | 100 |
|  | 0.50 | 85 |
|  | 0.80 | 63 |
| 200 | 0.20 | 110 |
|  | 0.50 | 82 |
| 250 | 0.20 | 111 |
|  | 0.50 | 84 |
|  | 0.80 | 65 |
| 350 | 0.20 | 88 |
|  | 0.50 | 80 |
|  | 0.80 | 65 |
| 450 | 0.20 | 80 |
|  | 0.50 | 70 |
|  | 0.80 | 60 |

# S2. Electrochemical cells


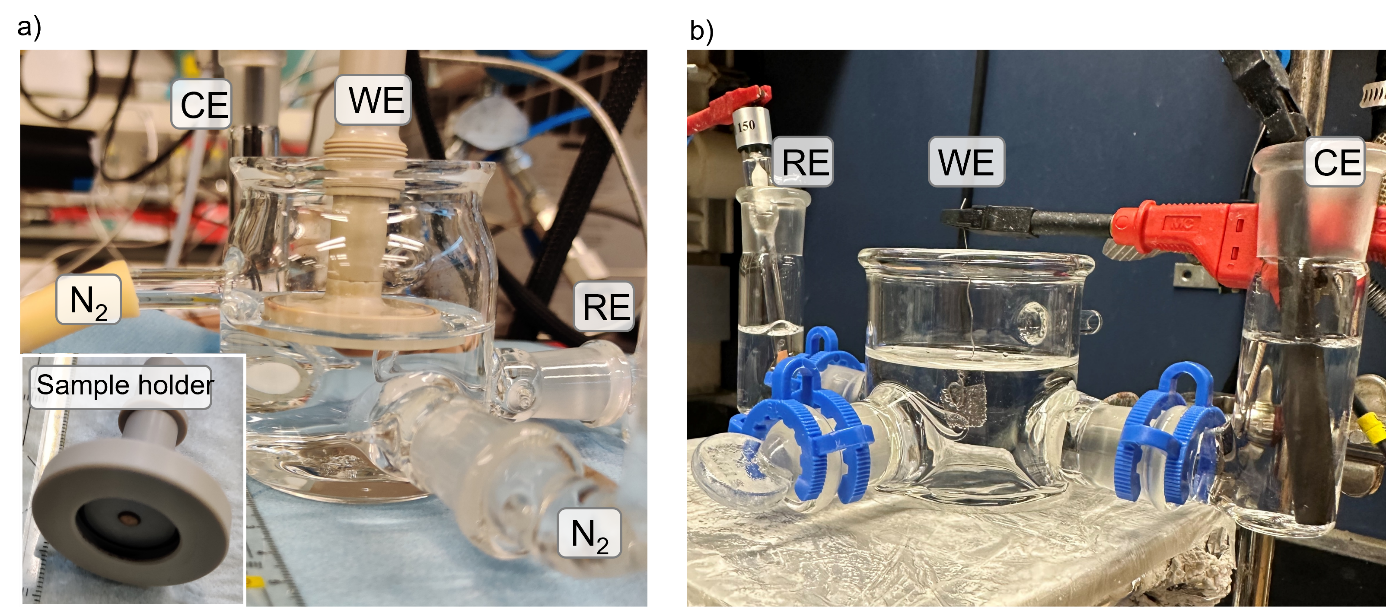


**Figure S2**. Photographs of the electrochemical cells used in this work. a) Cell used with MoS_x_/GC samples with the face-down catalyst connected to a rotating disk electrode apparatus. Inset shows the empty sample holder with a backside electrical contact, o-ring (not shown), and circular aperture that limits the active sample area. b) Cell used for stability studies with a vertical MoS_x_/carbon fiber paper sample held by a Ta clip. The N_2_ purge ports and CE and RE compartments equipped with a frit and a frit and a Luggin capilary, respectively, are indicated.

# S3. MoS_x_ catalysts of different thicknesses

Experiments were performed on the effect of thickness for both amorphous and crystalline MoS_x_ catalysts. For a-MoS_3.1_, we found that the electrochemical performance was independent of thickness for thicknesses of at least 4 nm (Figure S3). Thinner films resulted in both lower currents and poorer stability during repeated CV scans. For c-MoS_1.9_ films, a thickness of at least 5 nm yielded stable CVs with negligible change in HER current at larger thicknesses (Figure S4). We attribute the poor stability of the films in <1 to 2 nm range to likely discontinuity of these films, which may cause poor adhesion to the glassy carbon substrate. Incomplete surface coverage can also contribute to the lower activity of these samples. It is also possible that a catalyst thickness above a monolayer may be needed for optimal performance. This is due to the potential nanoscale porosity of the activated a-MoS_x_ catalysts as well as changes in morphology of c-MoS_2_ as a function of film thickness, in particular the formation of out-of-plane oriented crystallites exposing highly active edge sites. We chose to deposit approximately 7 nm thick films to ensure operation in range where thickness has little effect on electrochemical performance. Although not aimed at in this study, for cases where minimizing catalyst loading is imperative, we believe that catalysts thinner than 7 nm with good performance and stability can be prepared by PEALD.

The measurement parameters used in this section were slightly different to those presented in main text for the 7 nm samples. Three CV cycles at a scan rate of 5 mV/s were collected in potential range -0.2 to ‑0.9 V vs. Ag/AgCl. Thus, the total measurement time was slightly longer compared to five CVs in -0.2 to -0.75 V vs. Ag/AgCl range at 10 mV/s as done for the 7 nm samples discussed in the manuscript.


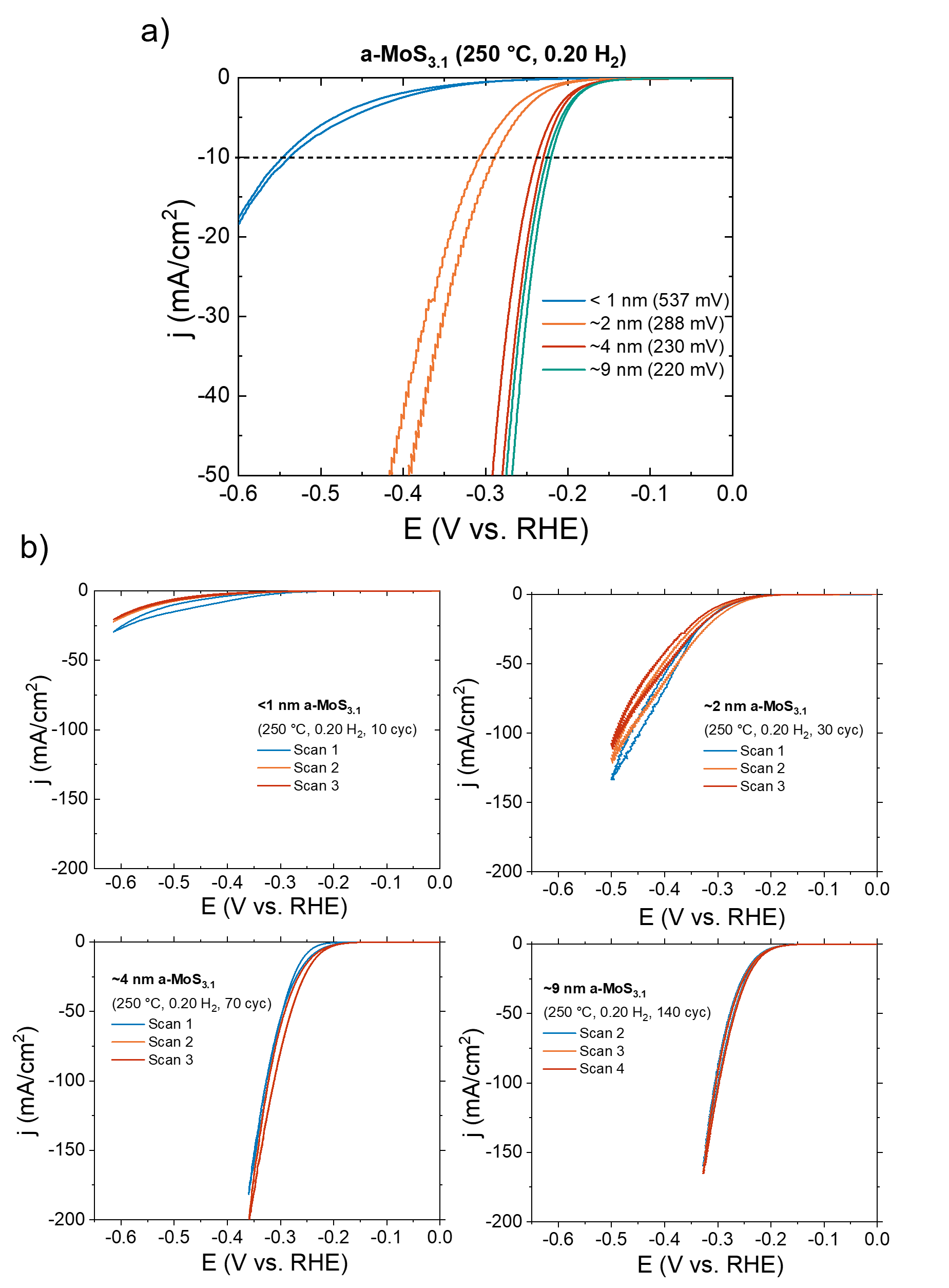


**Figure S3**. Electrochemical characterization of a-MoS_2+x_ films (a-MoS_3.1_ deposited at 250 °C, 0.20 H_2_) of different thicknesses (10, 30, 70, and 140 ALD cycles). a) Third CV for each sample with extracted η_10 mA/cm2_ values in parenthesis. b) Three CVs for each sample showing instability and poorer performance of the two thinnest films. For the thickest sample, the first CV showing electrochemical activation was not recorded.


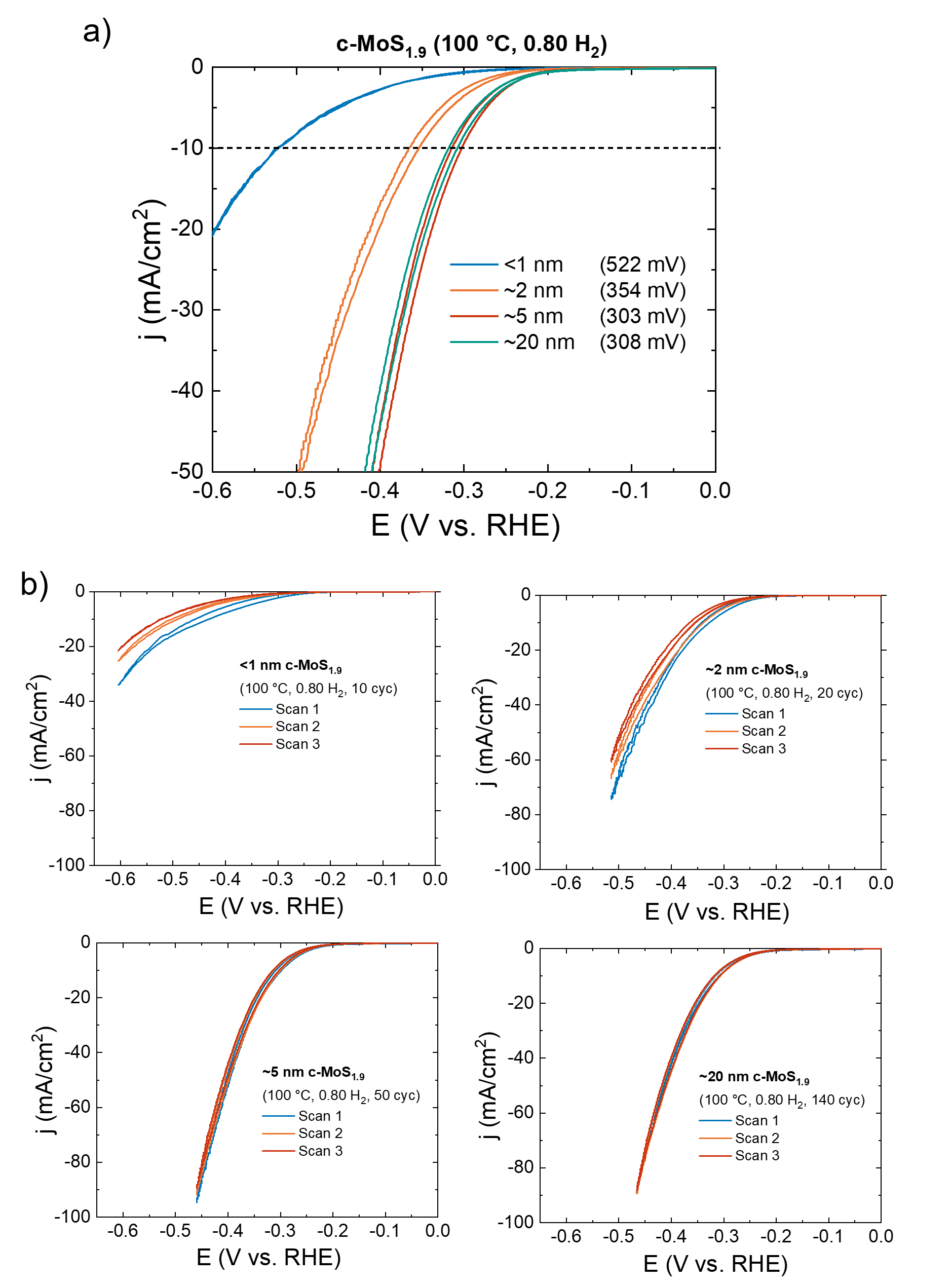


**Figure S4**. Electrochemical characterization of c-MoS_2_ films (c-MoS_1.9_ deposited at 100 °C, 0.80 H_2_ flow ratio) of different thicknesses (10, 30, 50, and 140 ALD cycles). a) Third CV for each sample with extracted η_10 mA/cm2_ values in parenthesis. b) Three CVs for each sample showing instability and poorer performance of the two thinnest films.

# S4. Electrochemical data of all 7 nm MoS_x_ catalysts


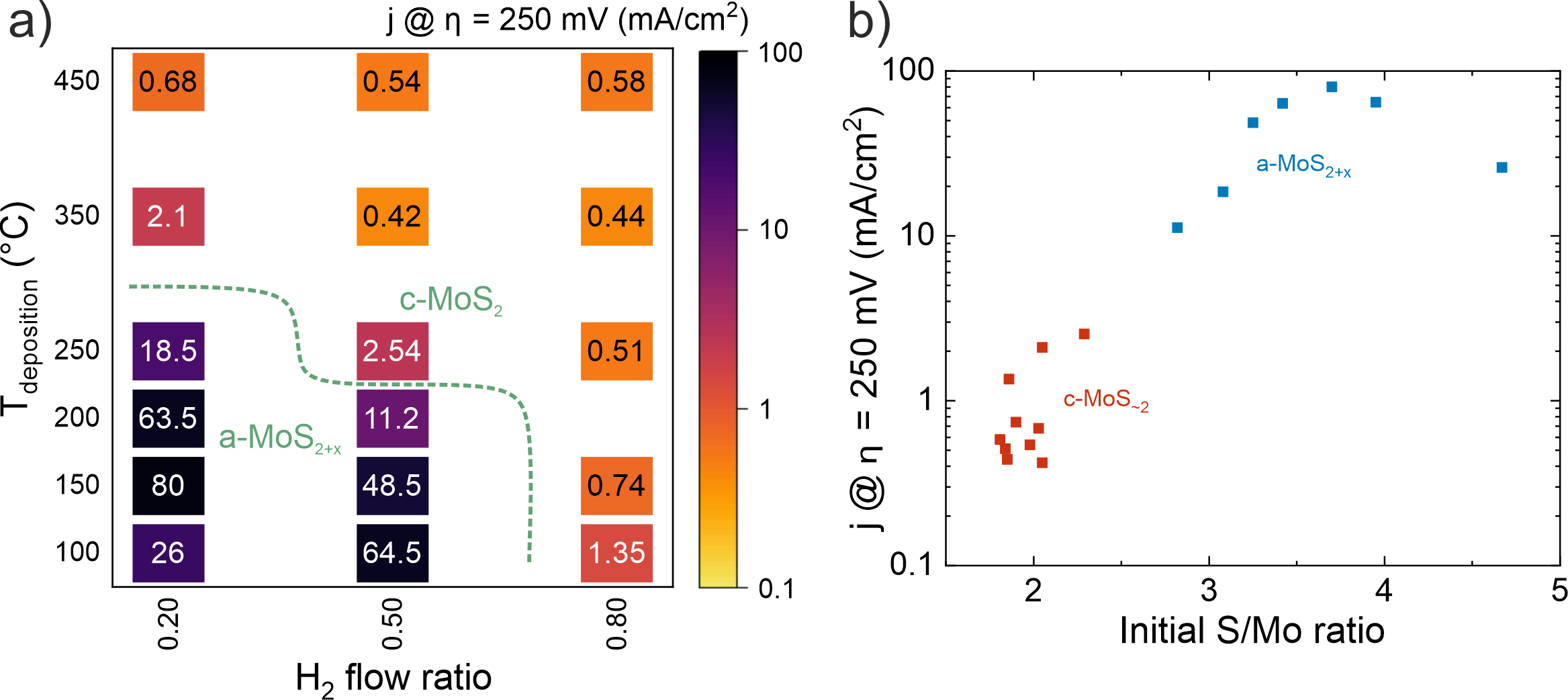


**Figure S5**. a) Heatmap of current density at overpotential of 250 mV versus deposition conditions shown in logarithmic color scale. b) Current density at overpotential of 250 mV (logarithmic scale) plotted against S/Mo ratio. The dashed green line indicates the border between amorphous and crystalline films.


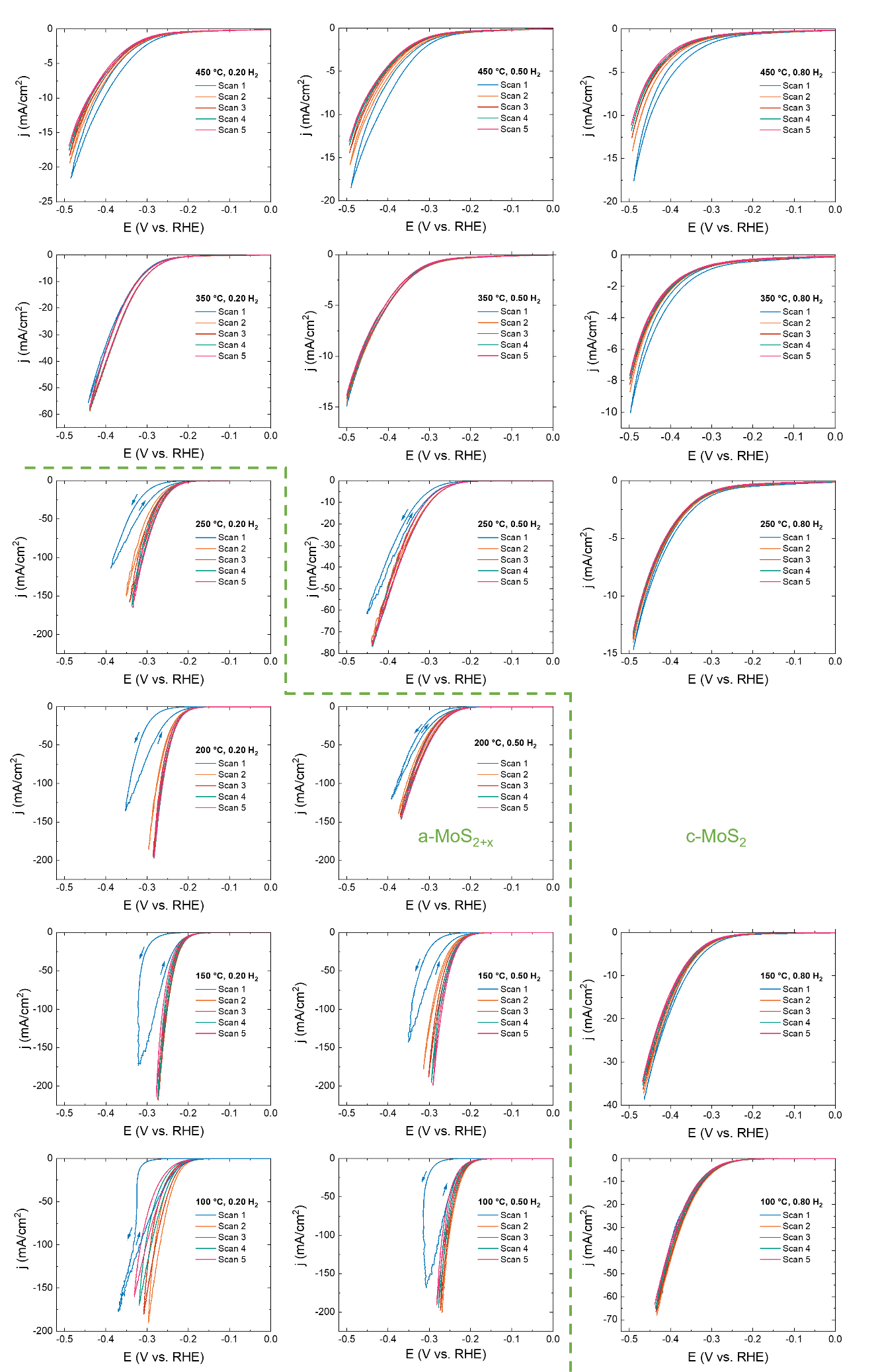


**Figure S6**. Five CVs (scan rate 10 mV/s) for 7 nm MoS_x_ catalysts prepared under different conditions (100% iR compensation). The a-MoS_2+x_ catalysts are shown in the same scale for comparison, while the c-MoS_2_ catalysts are plotted in different scales. The dashed green line indicates the border between amorphous and crystalline films.


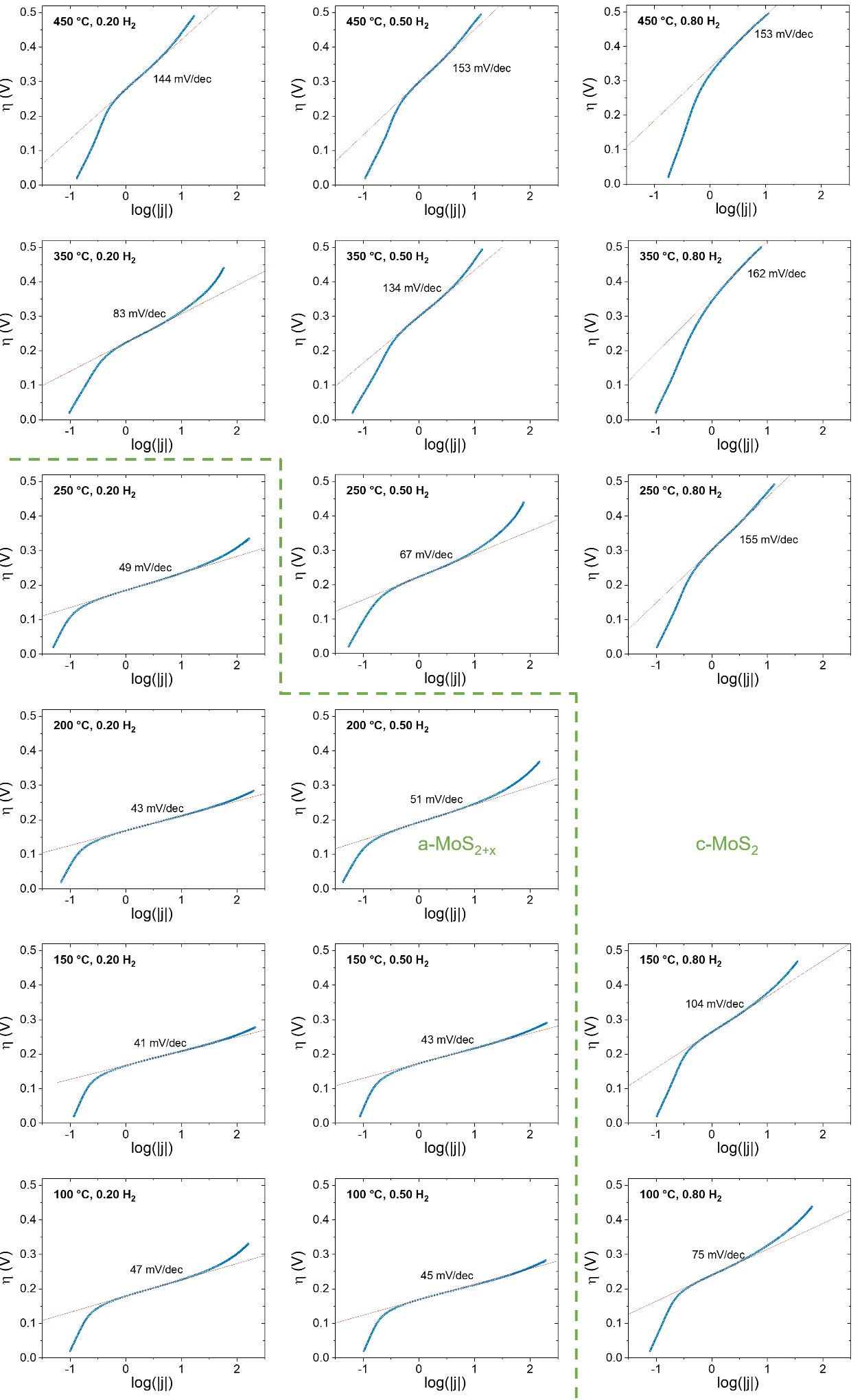


**Figure S7**. Tafel plots of 7 nm MoS_x_ catalysts prepared under different conditions (all in same scale, from forward trace of the 5th CV scan with 100% iR drop compensation). The slopes of lines fit to the linear portion (Tafel slope) are shown. The dashed green line indicates the border between amorphous and crystalline films.

# S5. Characterization of MoS_x_ catalysts as-deposited and after HER


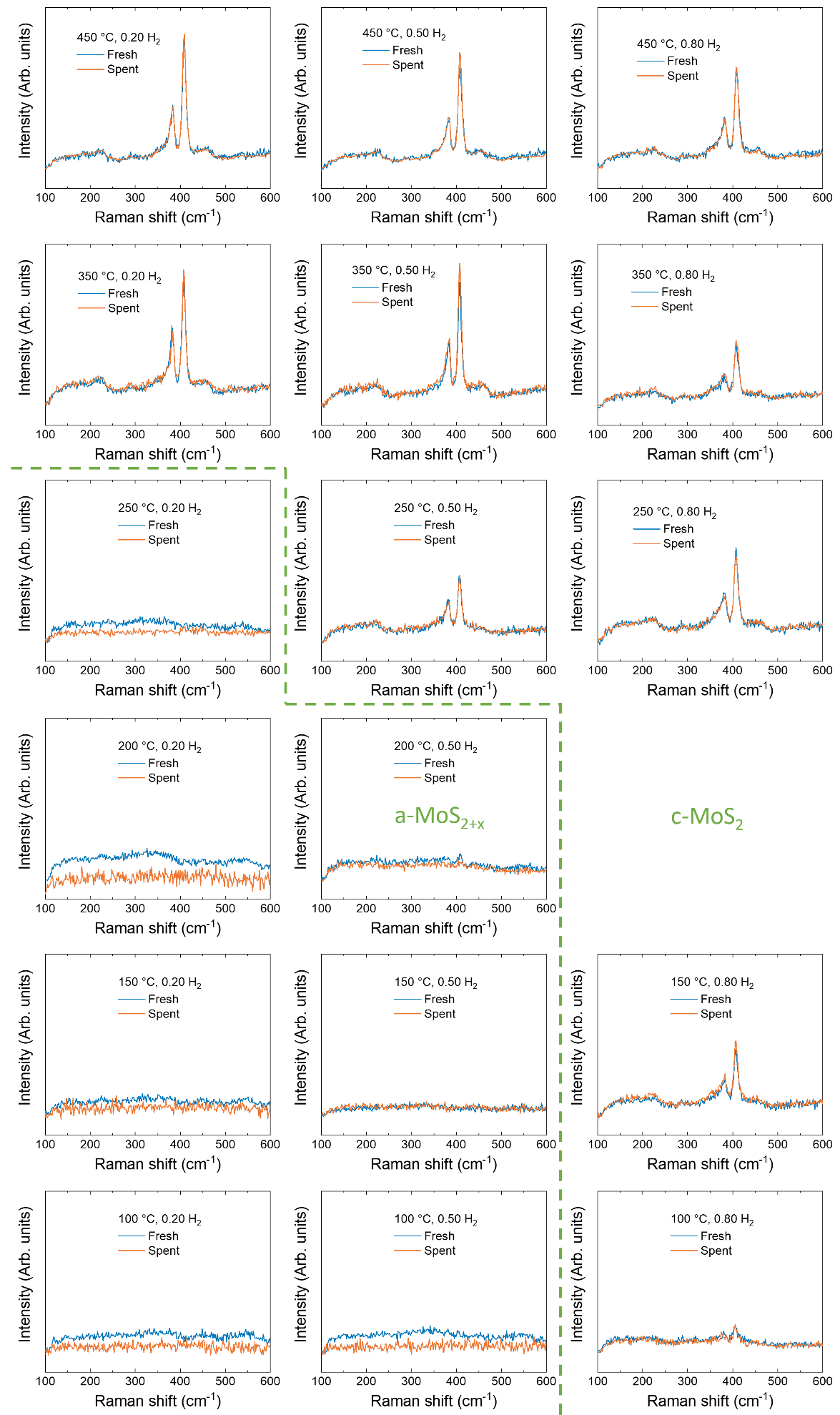


**Figure S8**. Raman spectra of 7 nm MoS_x_ catalysts prepared under different conditions on GC before (fresh) and after (spent) HER. No baseline subtraction or other spectral processing were performed. The dashed green line indicates the border between amorphous and crystalline films.


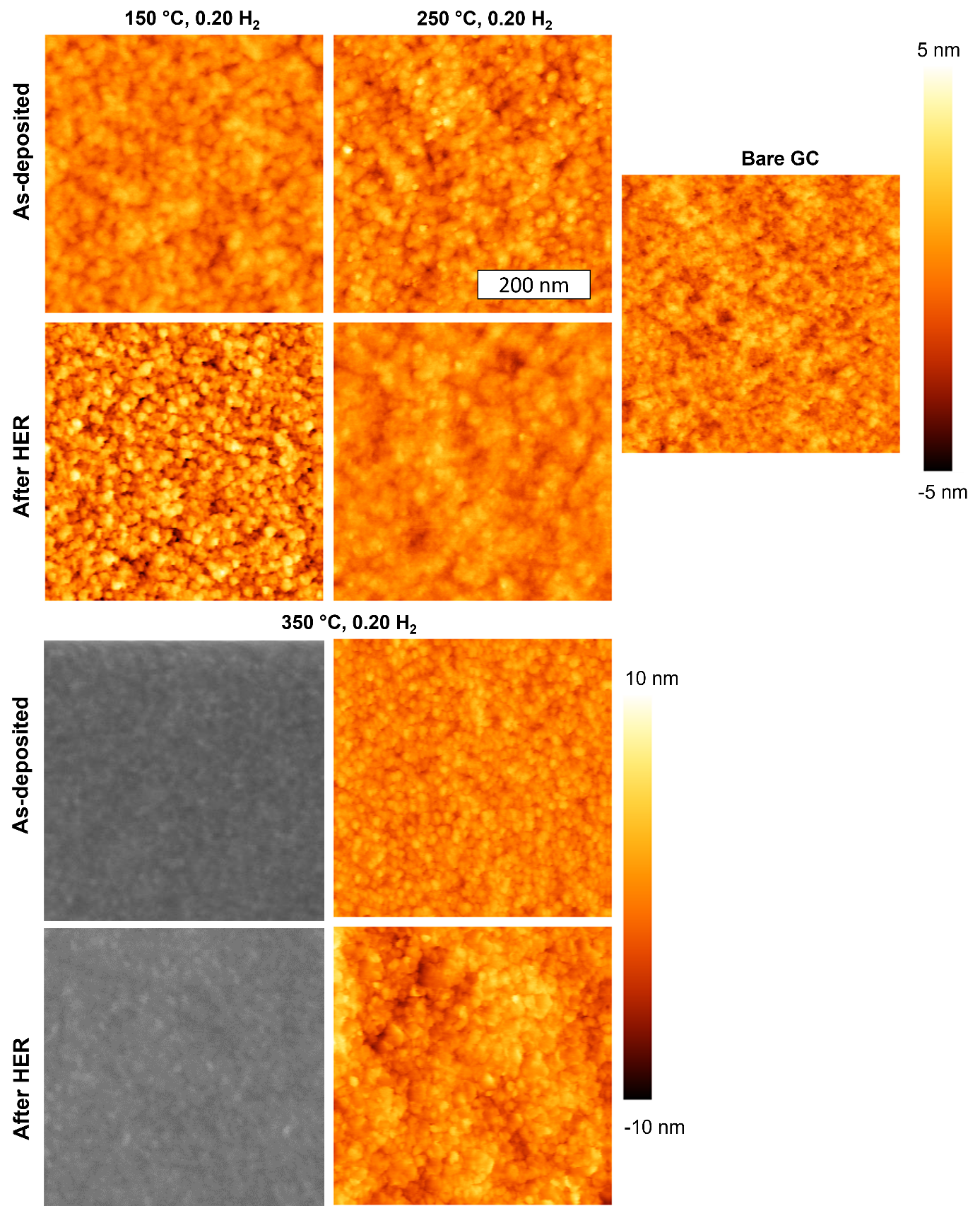


**Figure S9**. AFM (colored) and SEM (grayscale) images of selected MoS_x_ catalysts as-deposited and after HER on GC and a bare GC substrate. No significant morphology changes during HER were observed for any of the analyzed samples – the apparent differences are largely due to variation in GC surface morphology resulting from to the manual polishing procedure. Due to this substrate-to-substrate variation and the smooth nature of the majority of our MoS_x_ catalysts, we turned to samples deposited on SiO_2_/Si for a more comprehensive and reliable view on morphologies of the as-deposited samples (Figure S11). Regardless, in terms of surface area, all of the measured GC substrates were smooth enough to not appreciable increase surface area (<102% of geometric area).


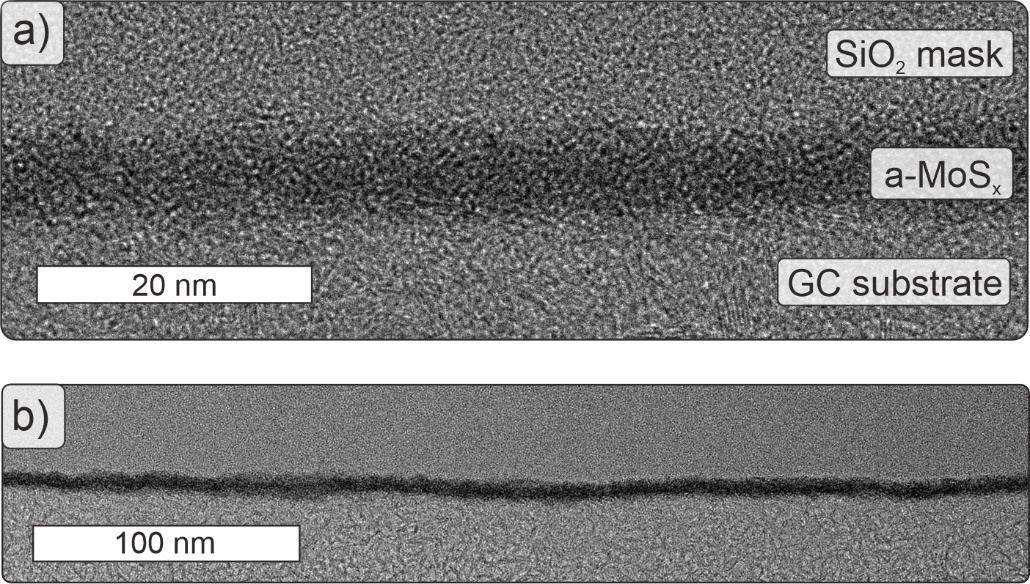


**Figure S10**. Cross-sectional TEM images of an “a-MoS_4.7_” catalyst after HER (deposited at 100 °C, 0.20 H_2_ flow ratio; final stoichiometry a-MoS_2.0_) on a GC substrate at a) higher and b) lower magnification. No crystallization or clear porosity of the a-MoS_x_ film were observed, while disordered graphite planes of GC are clearly visible.


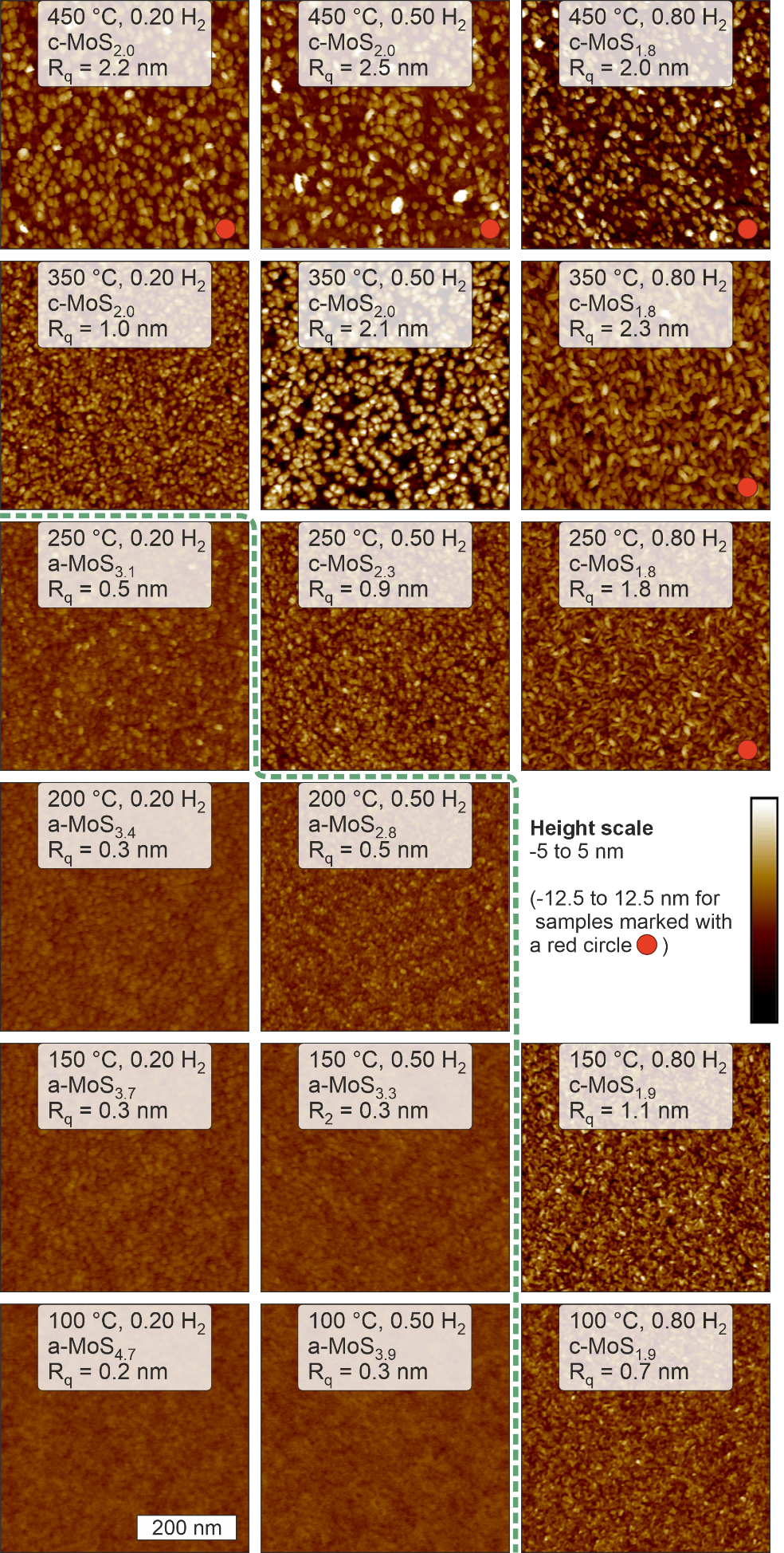


**Figure S11**. AFM images, roughness (R_q_) values, and initial XPS stoichiometries of 7 nm MoS_x_ films deposited on SiO_2_/Si. Due to the smoother and more repeatable SiO_2_/Si surface morphology compared to GC, films on SiO_2_/Si were used to illustrate effects of deposition conditions on film morphology. The dashed green line represents the border between amorphous and crystalline films.


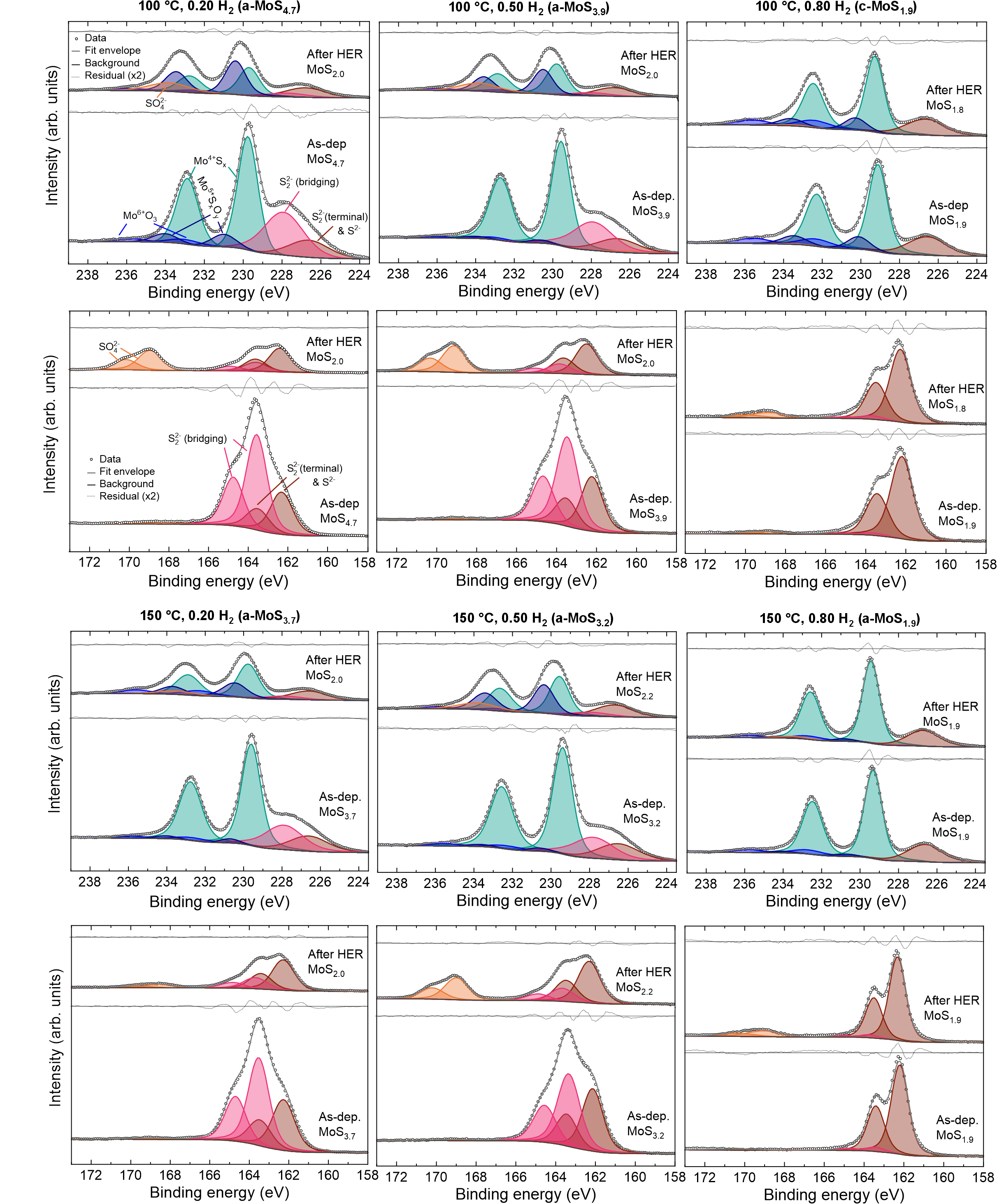


**Figure S12**. X-ray photoelectron spectra of MoS_x_ films deposited at 100 and 150 °C before and after HER experiments. Top and bottom row for each condition show the Mo 3d / S 2s and S 2p regions. The legend is shown for the 100 °C, 0.20 H_2_ flow ratio sample. Fit residuals are shown above each spectrum (multiplied by factor 2). Data for the other deposition conditions are shown on the following pages.

**
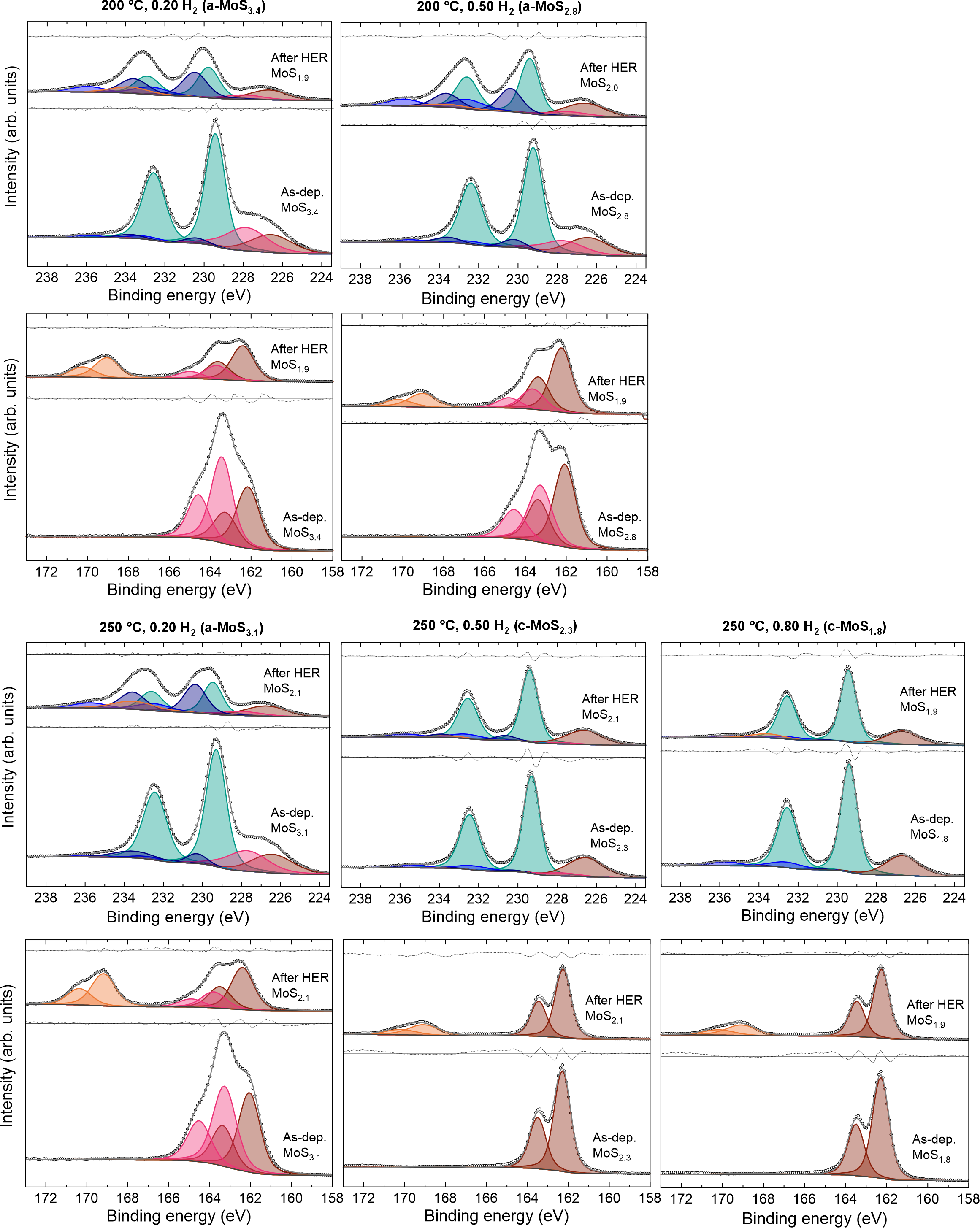
**

**Figure S13**. X-ray photoelectron spectra of MoS_x_ films deposited at 200 and 250 °C before and after HER experiments. See Figure S12 for more information.


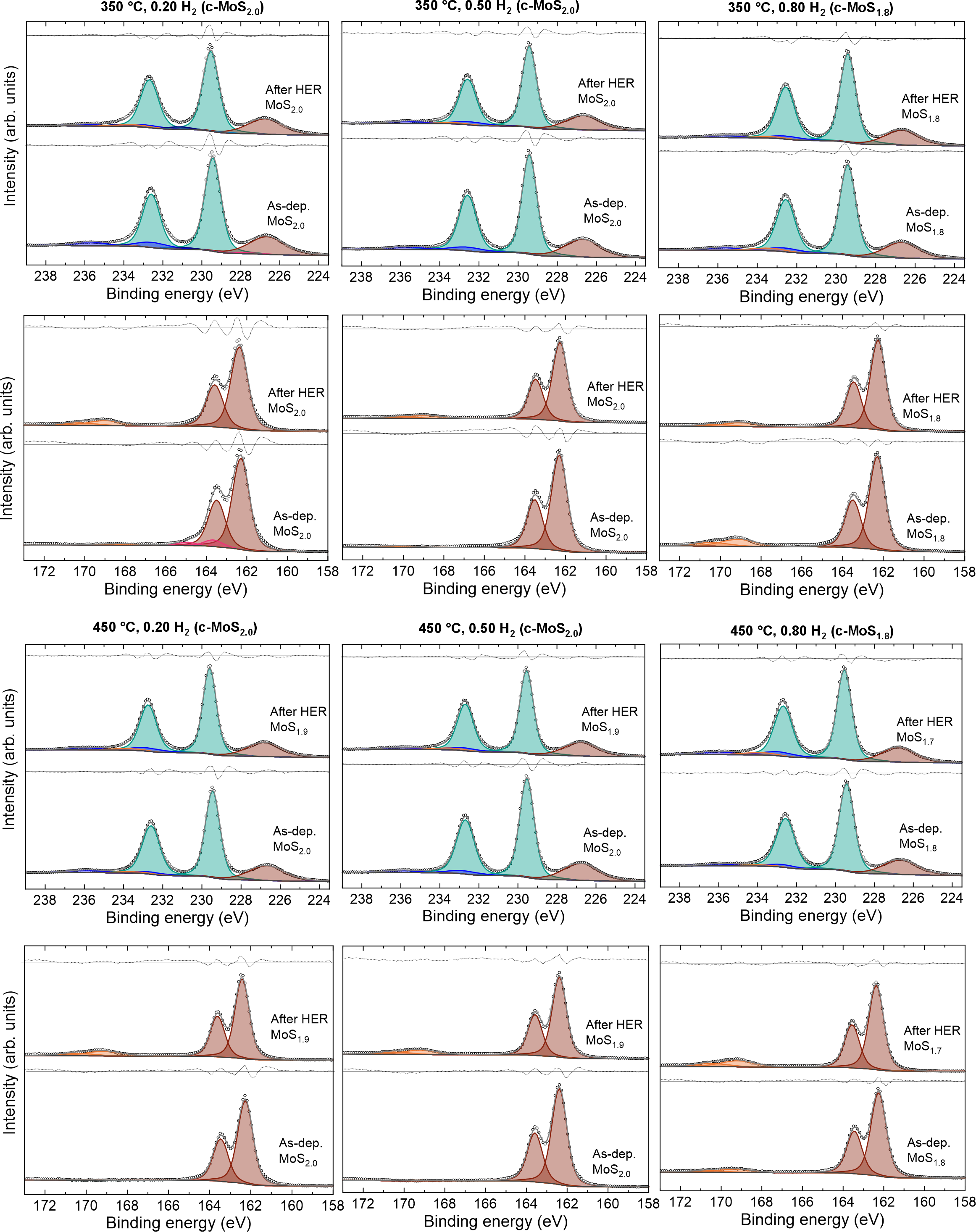


**Figure S14**. X-ray photoelectron spectra of MoS_x_ films deposited at 350 and 450 °C before and after HER experiments. See Figure S12 for more information.

**Table S2**. XPS fitting constraints for the Mo 3d / S 2s region. In addition, peak heights of the Mo 3d_3/2_ components were constrained to the Mo 3d_5/2_ to keep the areal ratio close to the values calculated by Scofield (area of Mo 3d_3/2_ to Mo 3d_5/2_ = 0.69±0.03).^1^ All the peaks were fit with a Voigt function (30%/70% Lorentzian/Gaussian). In addition, the absolute areas of the different S 2s components were fixed based on the S 2p fits (using RSF(S 2s) ≈ RSF (S 2p_3/2_) as supplied with the Avantage software). In addition, FWHMs of components in as-deposited and after-HER samples were fixed to be within 0.1 eV to each other.

| Peak | Position | FWHM |
| --- | --- | --- |
| Mo^4+^S_x_ (Mo 3d_5/2_) | 229.0–230.2 eV | 0.5–1.5 eV |
| Mo^4+^S_x_ (Mo 3d_3/2_) | above + (3.05–3.35 eV) | above ± 0.2 eV |
| Mo^5+^S_x_O_y_ (Mo 3d_5/2_) | Mo^4+^S_x_ (Mo 3d_5/2_) + (0.8–1.4 eV) | Mo^4+^S_x_ (Mo 3d_5/2_) ± 0.2 eV |
| Mo^5+^S_x_O_y_ (Mo 3d_3/2_) | above + (3.05–3.35 eV) | above ± 0.2 eV |
| Mo^6+^O_3_ (Mo 3d_5/2_) | 232.2–234.0 eV | 1.0–2.0 eV |
| Mo^6+^O_x_ (Mo 3d_3/2_) | above + (3.05–3.35 eV) | above |
| S_2_^2-^ (terminal) & S^2-^ (S 2s) | 225.5–227.0 eV | 1.5–2.5 eV |
| S_2_^2-^ (bridging) (S 2s) | above + (0.7–1.3 eV) | above |
| SO_4_^2-^ (S 2s) | 233.5±0.3 eV | above |

**Table S3.** XPS fitting constraints for the S 2p region. In addition, peak heights of the S 2p_1/2_ components were constrained to the S 2p_3/2_ to keep the areal ratio close to values calculated by Scofield (S 2p_1/2_ to S 2p_3/2_ = 0.51±0.01).^1^ All the peaks were fit with a Voigt function (30%/70% Lorentzian/Gaussian).

| Peak | Position | FWHM |
| --- | --- | --- |
| S_2_^2-^ (terminal) & S^2-^ (S 2p_3/2_) | 161.5–162.5 eV | 0.5–1.5 eV |
| S_2_^2-^ (terminal) & S^2-^ (S 2p_1/2_) | Above + (1.1–1.3 eV) | Above |
| S_2_^2-^ (bridging) (S 2p_3/2_) | S_2_^2-^ (terminal) & S^2-^ (S 2p_3/2_) + (1.0–1.4 eV) | S_2_^2-^ (terminal) & S^2-^ (S 2p_3/2_) ± 0.1 eV |
| S_2_^2-^ (bridging) (S 2p_1/2_) | Above + (1.1–1.3 eV) | Above |
| SO_4_^2-^ (S 2p_3/2_) | 168.0–170.0 eV | 1.0–2.0 eV |
| SO_4_^2-^ (S 2p_1/2_) | Above + (1.1–1.3 eV) | Above |

**Table S4**. XPS peak fitting of MoS_x_/GC catalysts deposited at different conditions before and after HER by *ex situ* XPS. The three rows from top to bottom represent binding energies, relative peak areas, and full-widths at half-maximum (FWHMs) of different Mo 3d and S 2p components. The SO_4_^2-^ component in *italic* is mostly attributed to residual electrolyte and thus excluded in calculating S/Mo ratios and relative intensities of the other S components. This interpretation is based on observation that significant SO_4_^2-^ was only detected in in samples after OER and that including sulfate often led to S/Mo ratios exceeding those of the as-deposited films. See Tables S2 and S3 for the fitting constraints used.

| Sample | S/Mo ratio | Mo 3d_5/2_ | | | S 2p_3/2_ | | |
| --- | --- | --- | --- | --- | --- | --- | --- |
|  |  | Mo^4+^S_x_ | Mo^5+^S_x_O_y_ | Mo^6+^O_3_ | S_2_^2-^ (terminal) & S^2-^ | S_2_^2-^ (bridging) | *SO_4_ ^2-^* |

| **100 °C, 0.20 H_2_**  As-dep. | 4.67 | 229.8 eV  (90%)  (1.2 eV) | 230.9 eV  (5%)  (1.2 eV) | 233.1 eV  (5%)  (2.0 eV) | 162.3 eV  (32%)  (1.3 eV) | 163.6 eV  (68%)  (1.2 eV) | *<1%* |
| --- | --- | --- | --- | --- | --- | --- | --- |
| After HER | 2.05 | 229.7 eV  (42%)  (1.2 eV) | 230.4 eV  (51%)  (1.3 eV) | 232.4 eV  (7%)  (2.0 eV) | 162.4 eV  (71%)  (1.3 eV) | 163.6 eV  (29%)  (1.4 eV) | *168.9 eV*  *(1.4 eV)* |
| **100 °C, 0.50 H_2_**  As-dep. | 3.95 | 229.6 eV  (94%)  (1.2 eV) | 230.7 eV  (3%)  (1.4 eV) | 233.3 eV  (3%)  (2.0 eV) | 162.3 eV  (35%)  (1.2 eV) | 163.5 eV  (65%)  (1.3 eV) | <1% |
| After HER | 2.03 | 229.8 eV  (53%)  (1.2 eV) | 230.5 eV  (40%)  (1.2 eV) | 232.6 eV  (7%)  (2.0 eV) | 162.5 eV  (75%)  (1.3 eV) | 163.8 eV  (25%)  (1.4 eV) | *169.1 eV*  *(1.3 eV)* |
| **100 °C, 0.80 H_2_**  As-dep. | 1.86 | 229.1 eV (74%)  (1.2 eV) | 230.1 eV (15%)  (1.4 eV) | 232.3 eV (11%)  (2.0 eV) | 162.2 eV (97%)  (1.3 eV) | 163.6 eV (3%)  (1.4 eV) | <1% |
| After HER | 1.82 | 229.3 eV (81%)  (1.2 eV) | 230.5 eV (10%)  (1.4 eV) | 232.7 eV (9%)  (2.0 eV) | 162.3 eV (95%)  (1.2 eV) | 163.7 eV (5%)  (1.5 eV) | *168.8 eV*  *(2.0 eV)* |
| **150 °C, 0.20 H_2_**  As-dep. | 3.70 | 229.6 eV (91%)  (1.2 eV) | 230.7 eV (4%)  (1.2 eV) | 232.7 eV (5%)  (2.0 eV) | 162.3 eV (38%)  (1.3 eV) | 163.5 eV (62%)  (1.3 eV) | <1% |
| After HER | 2.03 | 229.8 eV (59%)  (1.5 eV) | 230.5 eV (28%)  (1.5 eV) | 232.3 eV (13%)  (1.9 eV) | 162.3 eV (71%)  (1.3 eV) | 163.7 eV (29%)  (1.4 eV) | *168.5 eV*  *(1.7 eV)* |
| **150 °C, 0.50 H_2_**  As-dep. | 3.25 | 229.6 eV (93%)  (1.3 eV) | 230.7 eV (3%)  (1.2 eV) | 232.7 eV (4%)  (2.0 eV) | 162.1 eV (44%)  (1.2 eV) | 163.3 eV (56%)  (1.3 eV) | <1% |
| After HER | 2.19 | 229.6 eV (54%)  (1.2 eV) | 230.4 eV (41%)  (1.3 eV) | 232.6 eV (5%)  (2.0 eV) | 162.3 eV (76%)  (1.3 eV) | 163.7 eV (24%)  (1.4 eV) | *169.0 eV*  *(1.4 eV)* |
| **150 °C, 0.80 H_2_**  As-dep. | 1.90 | 229.3 eV (89%)  (1.1 eV) | 230.7 eV (3%)  (1.3 eV) | 232.6 eV (8%)  (2.0 eV) | 162.2 eV (96%)  (1.0 eV) | 163.4 eV  (4%)  (1.1 eV) | <1% |
| After HER | 1.89 | 229.4 eV (90%)  (1.1 eV) | 230.8 eV (3%)  (1.0 eV) | 232.7 eV (7%)  (2.0 eV) | 162.3 eV (96%)  (1.0 eV) | 163.7 eV  (4%)  (1.1 eV) | *169.0 eV*  *(2.0 eV)* |
| Sample | S/Mo ratio | Mo^4+^S_x_ | Mo^5+^S_x_O_y_ | Mo^6+^O_3_ | S_2_^2-^ (terminal) & S^2-^ | S_2_^2-^ (bridging) | *SO_4_ ^2-^* |
| **200 °C, 0.20 H_2_**  As-dep. | 3.42 | 229.4 eV (89%)  (1.2 eV) | 230.4 eV (6%)  (1.4 eV) | 233.0 eV (5%)  (2.0 eV) | 162.2 eV (44%)  (1.3 eV) | 163.4 eV (56%)  (1.2 eV) | <1% |
| After HER | 1.95 | 229.8 eV (46%)  (1.3 eV) | 230.5 eV (37%)  (1.5 eV) | 232.7 eV (17%)  (2.0 eV) | 162.4 eV (70%)  (1.3 eV) | 163.7 eV (30%)  (1.4 eV) | *169.0 eV*  *(1.4 eV)* |
| **200 °C, 0.50 H_2_**  As-dep. | 2.82 | 229.2 eV (86%)  (1.2 eV) | 230.2 eV (8%)  (1.4 eV) | 232.3 eV (6%)  (1.6 eV) | 162.1 eV (57%)  (1.3 eV) | 163.3 eV (43%)  (1.3 eV) | <1% |
| After HER | 2.00 | 229.4 eV (54%)  (1.2 eV) | 230.4 eV (29%)  (1.4 eV) | 232.6 eV (17%)  (2.0 eV) | 162.2 eV (75%)  (1.3 eV) | 163.6 eV (25%)  (1.3 eV) | *169.0 eV*  *(1.6 eV)* |
| **250 °C, 0.20 H_2_**  As-dep. | 3.08 | 229.3 eV (88%)  (1.2 eV) | 230.3 eV (8%)  (1.2 eV) | 232.8 eV (4%)  (2.0 eV) | 162.1 eV (48%)  (1.3 eV) | 163.3 eV (52%)  (1.4 eV) | <1% |
| After HER | 2.07 | 229.5 eV (42%)  (1.2 eV) | 230.4 eV (42%)  (1.4 eV) | 232.5 eV (16%)  (2.0 eV) | 162.4 eV (71%)  (1.3 eV) | 163.8 eV (29%)  (1.4 eV) | *169.2 eV*  *(1.3 eV)* |
| **250 °C, 0.50 H_2_**  As-dep. | 2.29 | 229.3 eV (90%)  (1.0 eV) | 230.3 eV (2%)  (0.8 eV) | 232.3 eV (8%)  (2.0 eV) | 162.2 eV (86%)  (1.1 eV) | 163.6 eV (14%)  (1.2 eV) | *<1%* |
| After HER | 2.08 | 229.4 eV (83%)  (1.0 eV) | 230.8 eV (7%)  (1.2 eV) | 232.7 eV (10%)  (2.0 eV) | 162.2 eV (92%)  (1.1 eV) | 163.6 eV (8%)  (1.2 eV) | *168.9 eV*  *(2.0 eV)* |
| **250 °C, 0.80 H_2_**  As-dep. | 1.84 | 229.4 eV (90%)  (1.0 eV) | 230.8 eV (1%)  (0.9 eV) | 232.6 eV (9%)  (2.0 eV) | 162.3 eV (100%)  (1.0 eV) | <1% | <1% |
| After HER | 1.89 | 229.4 eV (91%)  (1.0 eV) | 230.8 eV (2%)  (1.2 eV) | 232.6 eV (7%)  (1.6 eV) | 162.3 eV (100%)  (0.9 eV) | <1% | *169.1 eV*  *(1.8 eV)* |
| **350 °C, 0.20 H_2_**  As-dep. | 2.05 | 229.4 eV (88%)  (1.0 eV) | 230.8 eV (2%)  (1.2 eV) | 232.5 eV (10%)  (2.0 eV) | 162.3 eV (93%)  (1.0 eV) | 163.6 eV (7%)  (1.1 eV) | *<1%* |
| After HER | 2.04 | 229.5 eV (91%)  (1.0 eV) | 231.1 eV (4%)  (1.4 eV) | 232.8 eV (5%)  (2.0 eV) | 162.4 eV (100 %)  (1.0 eV) | <1% | *169.0 eV*  *(2.0 eV)* |
| **350 °C, 0.50 H_2_**  As-dep. | 2.05 | 229.4 eV (93%)  (0.9 eV) | <1% | 232.5 eV (7%)  (2.0 eV) | 162.3 eV (100%)  (0.9 eV) | <1% | *<1%* |
| After HER | 1.99 | 229.4 eV (91%)  (0.9 eV) | 230.8 eV (1%)  (1.1 eV) | 232.5 eV (8%)  (2.0 eV) | 162.3 eV (100%)  (0.9 eV) | <1% | *168.9 eV*  *(2.0 eV)* |
| Sample | S/Mo ratio | Mo^4+^S_x_ | Mo^5+^S_x_O_y_ | Mo^6+^O_3_ | S_2_^2-^ (terminal) & S^2-^ | S_2_^2-^ (bridging) | *SO_4_ ^2-^* |
| **350 °C, 0.80 H_2_**  As-dep. | 1.85 | 229.4 eV (91%)  (1.0 eV) | 230.8 eV (1%)  (1.2 eV) | 232.6 eV (8%)  (2.0 eV) | 162.3 eV (100%)  (0.9 eV) | <1% | *169.1 eV*  *(2.0 eV)* |
| After HER | 1.79 | 229.4 eV (93%)  (0.9 eV) | <1% | 232.5 eV (7%)  (2.0 eV) | 162.3 eV (100%)  (0.9 eV) | <1% | *169.0 eV*  *(2.0 eV)* |
| **450 °C, 0.20 H_2_**  As-dep. (SiO_2_/Si) | 2.03 | 229.5 eV  (92%)  (0.9 eV) | <1% | 232.7 eV  (8%)  (2.0 eV) | 162.3 eV  (100%)  (0.7 eV) | <1% | <1% |
| After HER | 1.92 | 229.6 eV (91%)  (0.8 eV) | <1% | 232.9 eV (9%)  (2.0 eV) | 162.4 eV (100%)  (0.8 eV) | <1% | *169.1 eV*  *(2.0 eV)* |
| **450 °C, 0.50 H_2_**  As-dep. (SiO_2_/Si) | 1.98 | 229.5 eV  (93%)  (0.9 eV) | <1% | 232.8 eV  (7%)  (2.0 eV) | 162.4 eV  (100%)  (0.8 eV) | <1% | *<1%* |
| After HER | 1.91 | 229.6 eV (92%)  (0.8 eV) | <1% | 232.9 eV (8%)  (2.0 eV) | 162.4 eV (100%)  (0.8 eV) | <1% | *169.2 eV*  *(2.0 eV)* |
| **450 °C, 0.80 H_2_**  As-dep. (SiO_2_/Si) | 1.81 | 229.4 eV  (97%)  (0.9 eV) | 230.4 eV  (1%)  (1.1 eV) | 232.9 eV  (2%)  (1.9 eV) | 162.3 eV  (91%)  (0.9 eV) | <1% | *169.3 eV*  *(9%)*  *(2.0 eV)* |
| After HER | 1.73 | 229.5 eV (91%)  (0.9 eV) | <1% | 233.0 eV (9%)  (2.0 eV) | 162.4 eV (100%)  (0.9 eV) | <1% | *169.2 eV*  *(2.0 eV)* |

# S6. Summary of physicochemical and electrochemical characterization

**Table S5**. Summary of physicochemical and electrochemical characterization of different films. Measurements were done on 7 nm films on GC except for the following properties: R_q_ and A_relative_ (projected surface area relative to geometric area) were analyzed for 7 nm films on SiO_2_/Si and resistivity (from Ref.^2^) for ~8–20 nm films on SiO_2_/Si. Stoichiometry was determined by XPS, crystallinity by Raman spectroscopy, R_q_ and A_relative_ by AFM, and ρ by four-point probe (thickness by spectroscopic ellipsometry).

| Deposition | | Film characteristics | | | | Electro-  chemistry | |
| --- | --- | --- | --- | --- | --- | --- | --- |
| T_dep_ (°C) | H_2_ flow ratio | Stoichiometry (Before🡪After HER) | Cryst. (c) / amorph. (a) | R_q_ (nm) and A_relative_ (%)  on SiO_2_/Si | ρ (Ωcm) on SiO_2_/Si from Ref.^2^ | η_10 mA/cm2_ (mV) | Tafel slope (mV/dec) |
| 100 | 0.20 | 4.7 🡪 2.0 | a | 0.2, 100 | >dl | 227 | 47 |
|  | 0.50 | 3.9 🡪 2.0 | a | 0.3, 101 | >dl | 211 | 42 |
|  | 0.80 | 1.9 🡪 1.8 | c | 0.7, 105 | 0.17 | 325 | 75 |
| 150 | 0.20 | 3.7 🡪 2.0 | a | 0.3, 101 | >dl | 208 | 41 |
|  | 0.50 | 3.3 🡪 2.2 | a | 0.3, 101 | 1000 | 217 | 43 |
|  | 0.80 | 1.9 🡪 1.9 | c | 1.1, 110 | 0.35 | 377 | 104 |
| 200 | 0.20 | 3.4 🡪 1.9 | a | 0.3, 102 | >dl | 211 | 43 |
|  | 0.50 | 2.8 🡪 2.0 | a | 0.5, 102 | 6.2 | 246 | 51 |
| 250 | 0.20 | 3.1 🡪 2.0 | a | 0.5, 102 | 65 | 234 | 49 |
|  | 0.50 | 2.3 🡪 2.1 | c | 0.9, 106 | 1.5 | 298 | 67 |
|  | 0.80 | 1.8 🡪 1.9 | c | 1.8, 121 | 1.1 | 468 | 155 |
| 350 | 0.20 | 2.0 🡪 2.0 | c | 0.5, 107 | 24 | 314 | 83 |
|  | 0.50 | 2.0 🡪 2.0 | c | 0.9, 116 | 100 | 462 | 134 |
|  | 0.80 | 1.8 🡪1.8 | c | 1.8, 124 | 5.2 | 520 | 162 |
| 450 | 0.20 | 2.0 🡪 1.9 | c | 1.0, 117 | 220 | 440 | 144 |
|  | 0.50 | 2.0 🡪 1.9 | c | 2.1, 119 |  | 471 | 153 |
|  | 0.80 | 1.8 🡪 1.7 | c | 2.3, 118 | 0.59 | 489 | 153 |


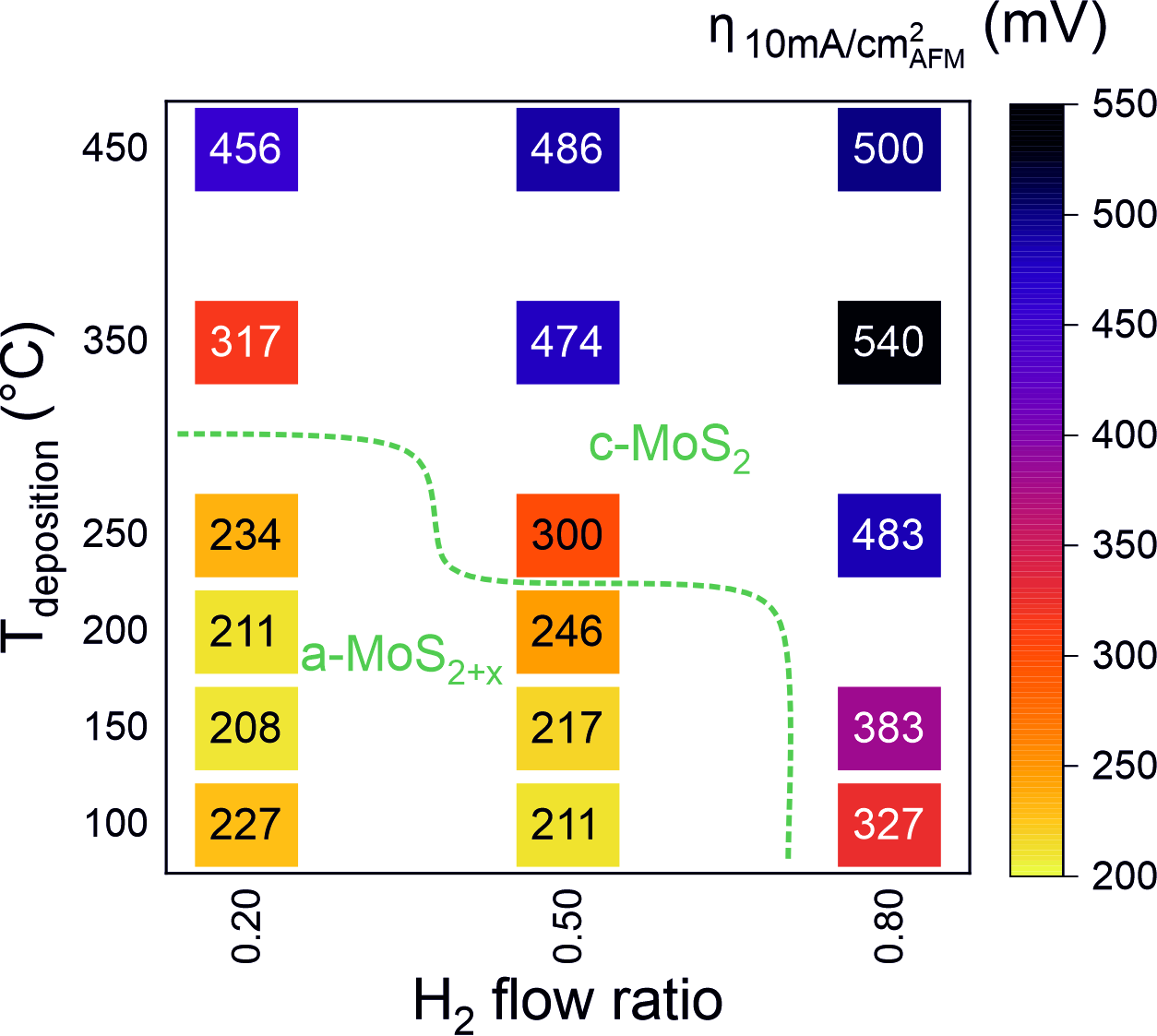


**Figure S15**. Overpotential using specific surface area from AFM, i.e. at 10 mA/cm^2^_AFM_. Comparison to overpotentials using geometric surface area (Table S5) shows the activity trends are unchanged. The dashed green line indicates the border between amorphous and crystalline films.

# S7. Quasi *in situ* XPS

**Table S6**. XPS peak fitting of MoS_x_ samples measured before (*ex situ* XPS) and after HER (quasi *in situ* XPS). The three rows from top to bottom represent binding energies, relative intensities, and FWHMs of different Mo 3d and S 2p components. The SO_4_^2-^ species in *italic* was excluded in calculated S/Mo ratios and relative intensities of the other S components, as it is attributed to residual electrolyte.

| Sample | S/Mo ratio | Mo 3d_5/2_ | | | S 2p_3/2_ | | |
| --- | --- | --- | --- | --- | --- | --- | --- |
|  |  | Mo^4+^S_x_ | Mo^5+^S_x_O_y_ | Mo^6+^O_3_ | S_2_^2-^ (terminal) & S^2-^ | S_2_^2-^ (bridging) | *SO_4_ ^2-^* |
| **100 °C, 0.20 H_2_** |  |  |  |  |  |  |  |
| As-dep. | 4.7 | 229.8 eV  (90%)  (1.2 eV) | 230.9 eV  (5%)  (1.2 eV) | 233.1 eV  (5%)  (2.0 eV) | 162.3 eV  (32%)  (1.3 eV) | 163.6 eV  (68%)  (1.2 eV) | *<1%* |
| After HER | 2.0 | 229.8 eV  (64%)  (1.2eV) | 230.6 eV  (29%)  (1.3 eV) | 232.3 eV  (7%)  (2.0 eV) | 162.5 eV  (69%)  (1.2 eV) | 163.8 eV  (31%)  (1.3 eV) | *169.3 eV*  *(1.4 eV)* |
| **250 °C, 0.20 H_2_** |  |  |  |  |  |  |  |
| As-dep. | 3.1 | 229.3 eV (88%)  (1.2 eV) | 230.3 eV (8%)  (1.2 eV) | 232.8 eV (4%)  (2.0 eV) | 162.1 eV (48%)  (1.3 eV) | 163.3 eV (52%)  (1.4 eV) | <1% |
| After HER | 2.0 | 229.3 eV  (72%)  (1.2 eV) | 230.2 eV  (22%)  (1.4 eV) | 232.6 eV  (6%)  (2.0 eV) | 162.2 eV  (81%)  (1.2 eV) | 163.4 eV  (19%)  (1.3 eV) | *169.2 eV*  *(1.8 eV)* |


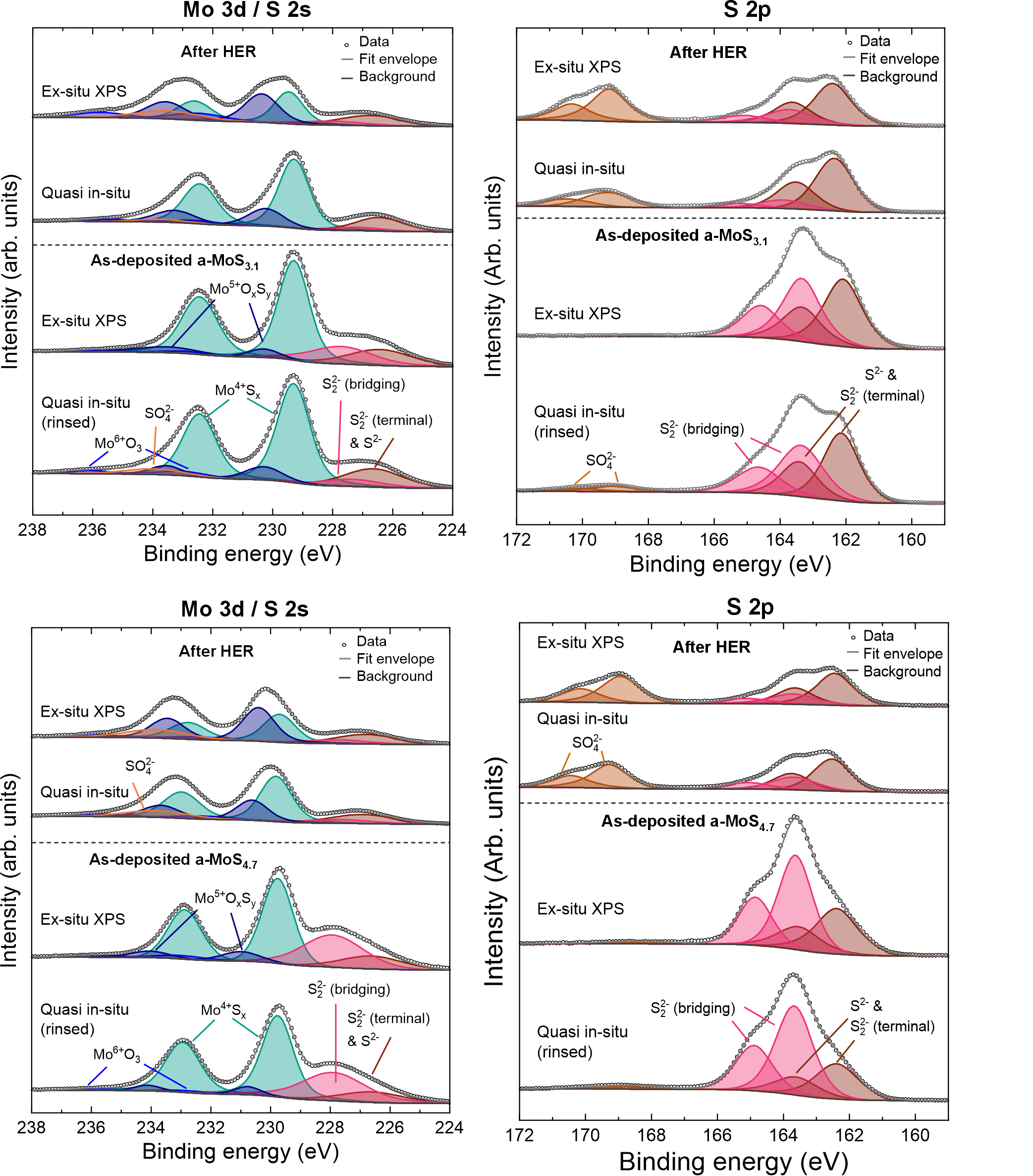


**Figure S16**. Comparison of X-ray photoelectron spectra of two a-MoS_x_ films measured with quasi *in situ* as well as *ex situ* XPS (Mo 3d / S 2s and S 2p regions). The quasi *in situ* spectra of the as-deposited sample represents an area that was dry during the HER experiment but was exposed to some electrolyte and H_2_O during rinsing and thus displays some changes compares to the as-deposited sample measured by *ex situ* XPS. For the after HER samples, the *ex situ* XPS measurements shows much more oxidation of Mo due to air exposure, while the S 2p spectrum is comparable in both quasi *in situ* and *ex situ* measurements.


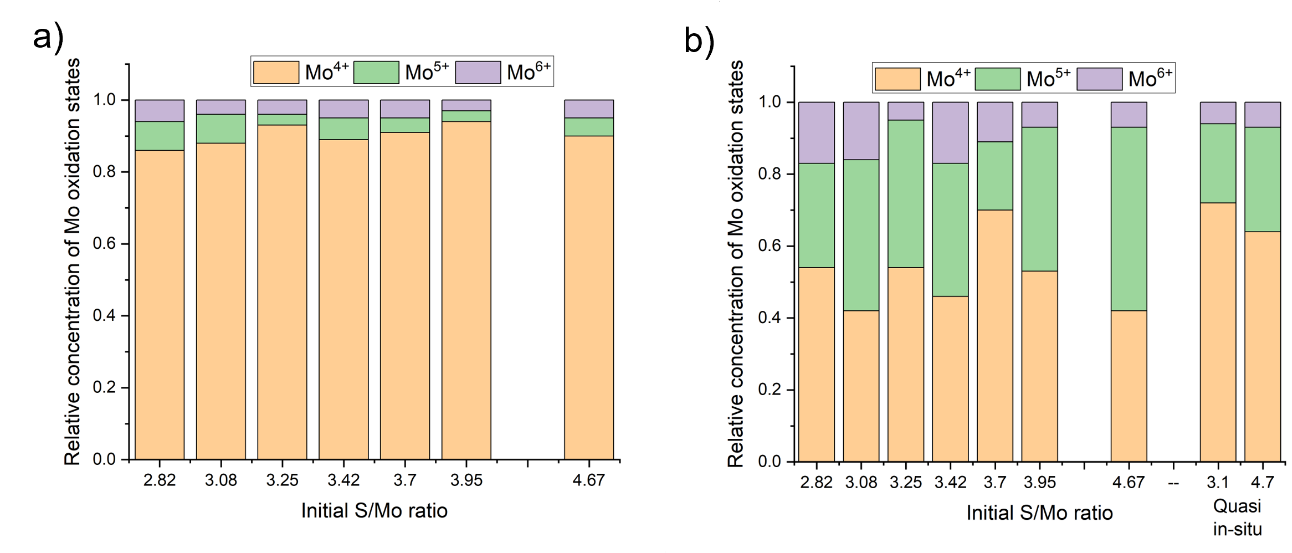


**Figure S17**. Relative concentrations of Mo^4+^, Mo^5+^, and Mo^6+^ species (of total Mo) in a-MoS_2+x_ as a function of initial S/Mo ratio for a) as-deposited and b) post-HER samples. The data is extracted from XPS fits shown in Tables S4 and S6.


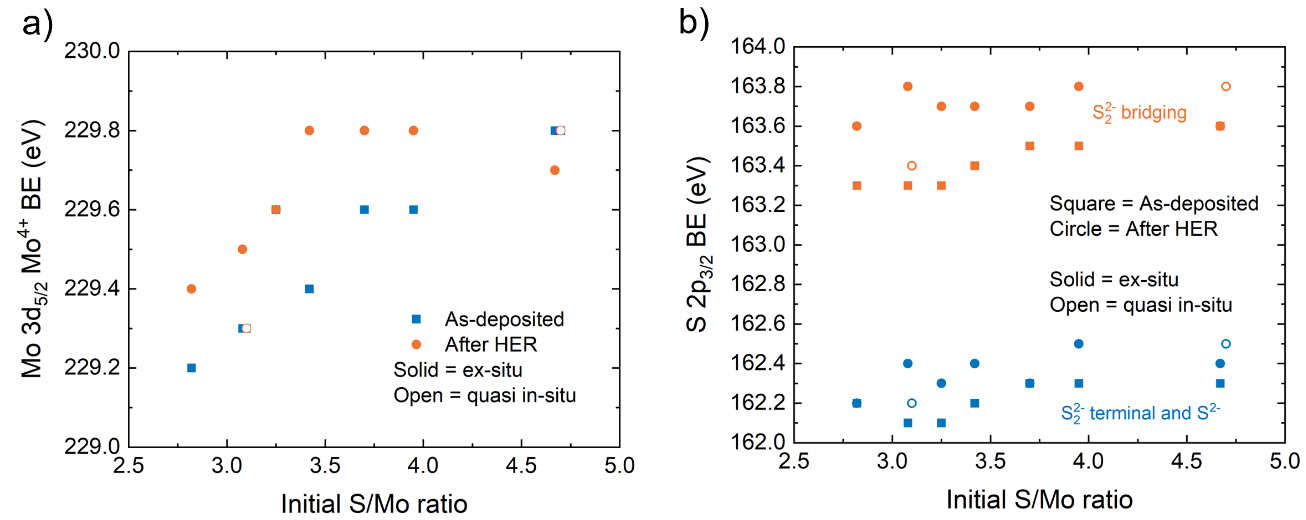


**Figure S18**. a) Mo 3d_5/2_ BE of Mo^4+^ species and b) S 2p_3/2_ (both fitted S components) of a-MoS_2+x_ catalysts as a function of initial S/Mo ratio for as-deposited and post-HER samples

# S8. Structure and active species of a-MoS_2+x_ HER catalysts

This section supplements Section 2.4 with additional discussion of literature on the structure of as-synthesized a-MoS_x_, the activation process prior to or under HER conditions, and the structure and catalytic species of the activated catalyst. Particular attention is paid to *operando* and *in situ* studies looking at these aspects under HER or HER-like conditions. In Table S7, we summarize studies proposing a broad range of active sites for a-MoS_x_ catalysts in acid.

**Structure of a-MoS_x_:** Two main types of structural models for as-deposited a-MoS_x_ have been proposed, but neither one of these has been clearly proven to be (in)correct. Preparation, stoichiometries, and structures of a-MoS_x_ have been reviewed by Afanasiev,^25^ Grayfer *et al.*,^26^ and Chang *et al.*^27^ The importance of understanding a-MoS_x_ structure in the context of HER lies in correctly assigning spectroscopic evidence and thus potential catalytically active sites of a-MoS_x_ as highlighted by Sahu *et al.*^28^ Figure S19 shows a selection of proposed structures that are discussed below.

Based on XPS, infrared spectroscopy, and chemical reactivity, in 1995 Weber *et al.*^29^ proposed a structure consisting of four different triangular Mo_3_S_9_ clusters connected by S bridges to describe a-MoS_3.1-3.2_ synthesized from (NH_4_)_2_[MoS_4_] (Figure S19a,b). A triangular cluster structure was earlier suggested by Müller and coworkers^30–32^ based on extrusion experiments, although such experiments cannot directly prove the presence of clusters in a-MoS_3_. Regardless, triangular Mo_3_S_x_ clusters (in particular [Mo_3_S_4_]^4+^ and [Mo_3_S_7_]^4+^) are well-known coordination compounds.^33,34^

Besides the work of Weber *et al.*, related cluster models based on different Mo_3_S_x_ moieties have been developed for S/Mo ratios varying from up to 4.5 (Figure 18c).^15,22,35–37^ Cluster models accounting for presence of O as a result of the deposition method used or oxidation in the atmosphere have also been devised (Figure S19d).^23,35,38^ Excluding oxidation, which forms some Mo^5+^, these cluster models are usually claimed to contain Mo exclusively as Mo^4+^, while S is present as both (bridging and sometimes also triply-coordinated apical) S^2-^ and (bridging and terminal) S_2_^2-^ species. Regardless, examination of the drawn structures shows that many of them actually show variation in formal oxidation state of Mo due to the proposed apical S^2-^ species^15,22,29,35^ and in some cases the average Mo oxidation state also differs from 4.^22,23,37^

The most questioned of the sulfur species is the apical S^2-^, which leads to difficulties in drawing extended structures with desired stoichiometry and retaining Mo oxidation state of +4. Furthermore, when present in certain individual Mo_3_S_x_ clusters, it has been found to be eliminated at rather low temperatures.^36,37^ Also, the proposed chain models do not contain any apical S^2-^ species. For higher S/Mo ratios near and above 4 it is also difficult to account for apical (or any other) S^2-^ considering charge balance. Therefore, we consider apical S^2-^ unlikely to be present in our films in significant quantities and do not account for it in the XPS fitting. The vast majority but not all^39^ of a-MoS_x_ HER studies use cluster models. To this end, we note that various Mo_x_S_y_ clusters have been claimed as HER catalysts by themselves, though little is known to how their structure changes prior or during HER, i.e. whether they polymerize and/or otherwise reconstruct, such as lose (a fraction of) S.^39–42^

Before the popularity of the cluster models, in the 1980s, Liang and coworkers^43–45^ used XAS and XRD to propose a chainlike model for a-MoS_3_ with a formula Mo^5+^(S^2-^)_2_(S_2_^2-^)_1/2_ depicted in Figure S19f. Hibble and coworkers used XAS,^46^ neutron diffraction,^47,48^ and reverse Monte Carlo simulations,^48^ finally proposing another chain model for a-MoS_3_ (Figure S19g), which has identical average oxidation states to the cluster model, i.e. Mo^4+^(S_2_^2-^)(S^2^). DFT calculations of Sahu *et al.*^28^ found the Mo^4+^ chain to be slightly more favorable compared to Mo^5+^ containing chain.

For more sulfur-rich a-MoS_x_, Hibble *et al.*^49,50^ suggested a chain model for a-MoS_4.7,_ describing it as Mo_4.7_(S_2_^2-^)_2.35_, but later found a cluster model to be more appropriate for a-MoS_4_ and a-MoS_4.5_ that were prepared in a different manner.^36^ Artemkina *et al.*^51^ proposed a chain structure for a-MoS_5_ containing S_2_^2-^ present in terminal as well as two types of bridging arrangements (Figure S19h). The chain models, especially of more S-rich materials, bear resemblance to the well-known dimeric [Mo_2_S_12_]^2-^ clusters (Figure S19i). In addition, group 5 chalcogenides such as NbS_3_ and NbS_4_ are known to crystallize into chainlike structures containing S_2_^2-^ dimers in terminal and bridging arrangements, respectively (for the structures of these and other polychalcogenides see Ref.^26^).

The majority of the MoS_x_ HER studies assume a cluster model to describe the (as-prepared) catalyst. It is likely that the different stoichiometries and even deposition methods of studied a-MoS_x_ films affect the structure. Afanasiev and Bezverkhy^52^ found a-MoS_3_, a-MoS_5_, and a-MoS_6_ samples to yield similar XAS data, suggesting that they all consisted of similar Mo_x_S_y_ units interconnected by a different amount and perhaps different types of S bridges. In contrary, Poltarak *et al.*^53^ modeled decomposition of a-MoS_5_ to a-MoS_3_ and suggested this to be accompanied by a change from a chain to a cluster structure. Hibble *et al.*^36^ – proponents of chain model for a-MoS_3_ – suggested a-MoS_4_ and a-MoS_4.5_ obtained by thermal decomposition from ammonium thiomolybdate salts to consist of Mo_3_S_12_ and Mo_3_S_14_ clusters. Remarkably, based on XAS, the latter was suggested to contain a tetrasulfide anion (S_4_^2-^), a rather common occurrence in Mo coordination chemistry.^25,33^ It is possible that both chain- and cluster-like a-MoS_x_, perhaps even mixtures of the both, may exist, as is suggested by XAS studies following chemical extrusion by Hibble *et al.*^54^ In terms of stoichiometry, it is interesting that the structure of a-MoS_~2_, such as that made by cathodic electrodeposition, does not appear to have been studied in detail.

The flexible coordination of S can explain varying S/Mo ratios in multiple ways. Clusters of different S/Mo ratios may form (containing S^2-^, apical S^2-^, bridging S_2_^2-^, and/or terminal S_2_^2-^) and may be linked in different ways, such as via (in the order of increasing S/Mo ratio) bridging S^2-^, bridging S_2_^2-^, and polysulfides S_n_^2-^ such as tetrasulfide (S_4_^2‑^). In chain structures, these different S species as both bridging (part of chain) and terminal (“on the side of chain”) can flexibly change stoichiometry. Different S ligands S^2-^, S_2_^2-^ and S_4_^2-^ can theoretically give rise to maximum S/Mo ratios of 2, 4, and 8, respectively, with Mo as 4+. In Figure S20, we present possible structures for selected a-MoS_2+x_ stoichiometries produced in this work based on the cluster models and our XPS results.

Besides the local coordination, the longer-range structure which is characteristically disordered in amorphous materials like a-MoS_x_, has been explored. Tran *et al.*^35^ showed transmission electron microscopy images supporting a branched chain composed of Mo_3_S_y_ clusters, albeit for an extremely low material loading not corresponding to typical a-MoS_x_ catalysts. Artemkina *et al.*^51^ approached this problem via molecular dynamics simulations, favoring a chain model for a-MoS_5_ where rotationally labile, linear bridging S_2_^2-^ groups connect Mo_2_S_10_ units into a flexible chain that can then pack intoglobule-like polymers. Hibble *et al.*^48^ proposed a 3D packing of their chain structure based on MD simulations, while Sahu *et al.*^28^ were able to reproduce similar structure by DFT.


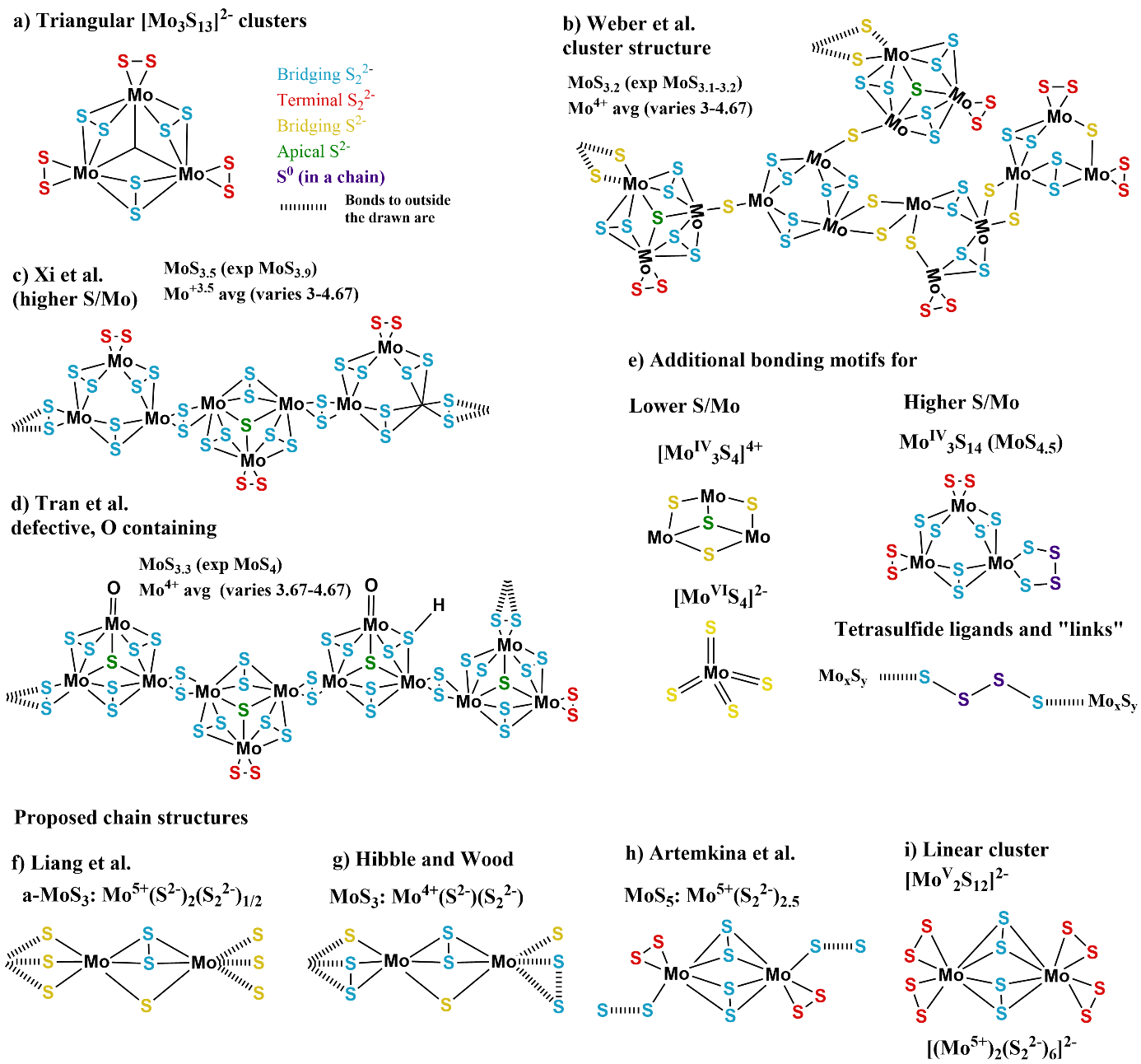


**Figure S19**. **Various structures proposed for a-MoS_x_ and related bonding motifs and clusters**. a) [Mo_3_S_13_]^2-^ cluster (also referred to as [Mo_3_S_7_]^4+^ with additional terminal disulfide ligands) used to motivate Mo_3_S_y_ based cluster models proposed by b) Weber *et al.*^29^ and modified for c) higher stoichiometries (Ref.^22^) and d) O-containing defective material (Ref.^35^). e) Additional bonding motifs and structures with which cluster-type structures of lower S/Mo ratios heavy in S^2-^ species [Mo_3_S_4_]^4+^, Ref.^58^ and [MoS_4_]^2-^) and higher S/Mo ratios heavy in S_2_^2-^ and S_n_^2-^ such as tetrasulfide (S_4_^2-^) species (Refs.^33,36^) can be constructed. Chain structures for a-MoS_3_ of f) Liang *et al.*^43^ and g) Hibble and Wood^48^ and h) Artemkina et al.^51^ for a-MoS_5_ and i) related dinuclear [Mo_2_S_12_]^2-^ cluster^12,59^. Note that Mo-Mo bonding present in the majority of the structures are omitted for clarity.


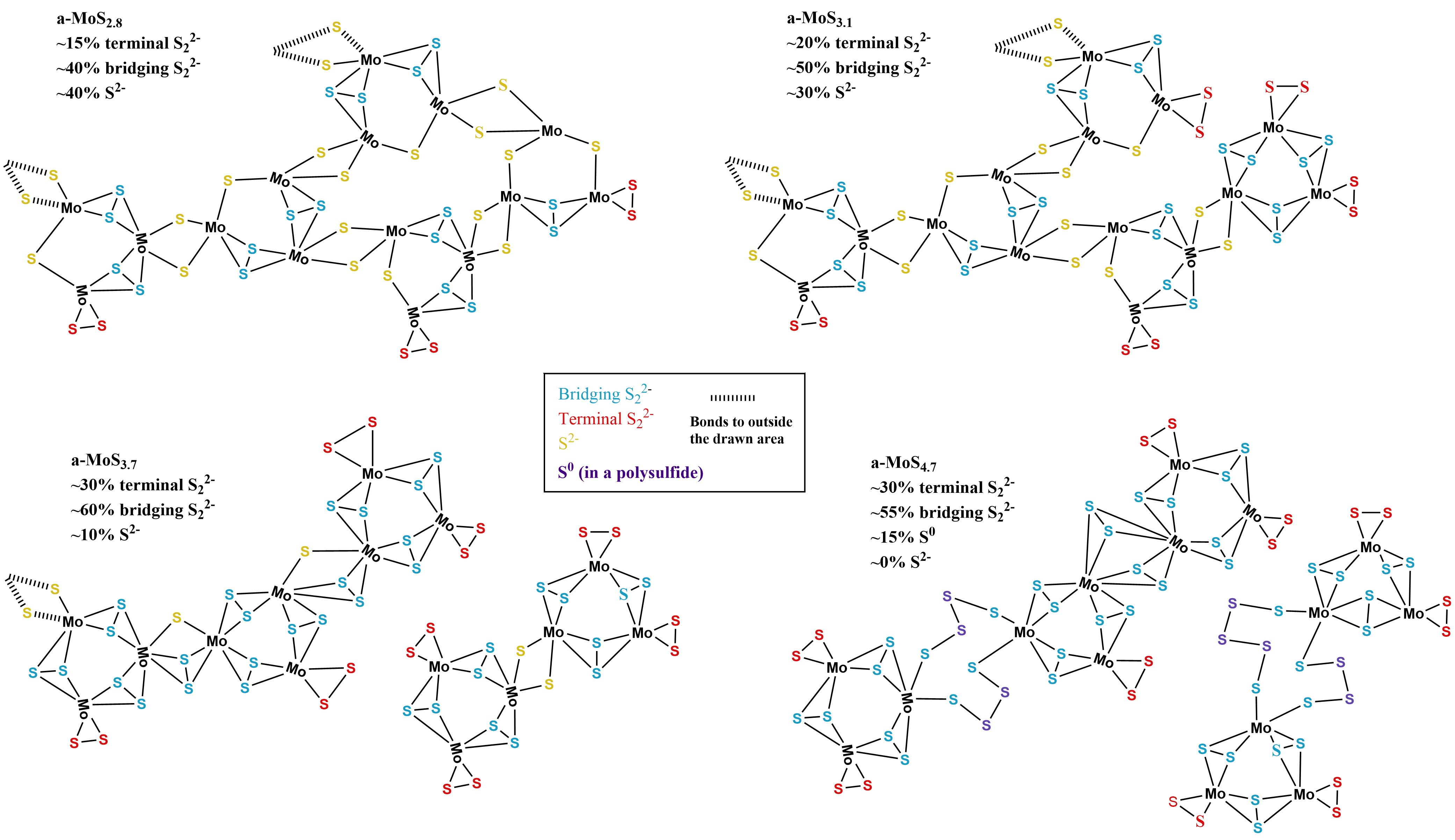


**Figure S20**. Illustration of bonding motifs in as-deposited a-MoS_2+x_ catalysts of different stoichiometries based on cluster model and our XPS data. All Mo is present as Mo^4+^ and thus changes occur in bonding and oxidation state of sulfur. Note that S^0^ species were not fit by XPS but are considered necessary for the highest S/Mo ratios (>4) to retain charge neutrality.

**Activation and composition of activated catalyst:** For a-MoS_2+x_, electrochemical activation combined with the loss of sulfur has been commonly observed. Several studies report S/Mo ratios ranging from 1.6 to 2.0 for the activated catalyst.^5,10,21,22^ However, for MoS_6_ catalysts made by electrodeposition as well as wet chemistry only partial loss of sulfur until MoS_4.2-5.0_ stoichiometry was observed.^12,13^ Electrodeposited^10,15^ and ALD^3^ catalysts with low initial S/Mo ratios of 1.7–2.0 have been reported not to undergo notable structural or compositional changes. We observed that the activation results in a-MoS_2.0_ films irrespective of the starting stoichiometry (MoS_2.7–4.5_). Thus, a-MoS_2.0_ appears to be the most common composition of the activated a-MoS_x_ catalyst, but the deposition method and initial stoichiometry may affect the structure of the as-deposited and, even more importantly, of the activated catalyst.

Few studies have examined the structure of the activated catalyst in detail. Albeit not under HER conditions, Afanasiev *et al.*^55^ exposed a-MoS_3_ and a-MoS_6_ to H_2_ gas at room temperature. The similarity of XAS data of both compounds was taken as evidence of their structural similarity, and was found to better correspond to chain rather than cluster model. Interestingly, no major change in XAS data was observed despite the loss of ~30% (~15%) of S from MoS_6_ (MoS_3_). However, the final S/Mo ratio was still ~4 and ~2.5 after reducing a-MoS_6_ and a-MoS_3_. During HER, *in situ* / *operando* Raman and X-ray absorption spectroscopy studies described below have highlighted changes in bonding during the activation and sulfur loss, but no detailed structural models have been presented.

Some authors have observed formation of (nano)crystalline MoS_2_ under HER conditions,^22^ leading to suggested presence of highly HER active S-terminated edges of MoS_2_ crystallites.^56,57^ In other cases, including the present study, no signs of crystallization have been observed. The potential where the electrochemical activation occurs also seems to depend on the deposition method, which may reflect differences in the initial catalyst structure. Our measurements show the activation to occur at approximately –0.30 to –0.35 V vs. RHE. Some other studies have reported activation to begin at more positive potentials, such as 0 to –0.1 V vs. RHE for electrodeposited a-MoS_3_.^14,15^

During the activation process, part of the sulfur is lost. This likely occurs via release of gaseous H_2_S as observed by Xi et al.^22^. One possible pathway begins with reduction of S_2_^2-^ and adsorption of H^+^, as represented here for a terminal disulfide site:

Mo-S_2_^2-^ + H^+^ + 2e^-^ 🡪 Mo-S^2-^ + Mo-SH (where Mo refers to the same Mo atom), followed by

Mo-SH + H^+^ 🡪 Mo + H_2_S, after which H_2_S gas is released

The process may also yield undersaturated Mo sites, which can react with, for example H_2_O to form Mo=O or Mo-OH species as suggested by our XPS measurements.

***Operando* / *in situ* studies on a-MoS_x_ catalysts:** *Operando* and *in situ* studies performed during HER and the preceding activation, although challenging to perform, can yield more direct insights into catalytic activity than typical before-after (*ex situ*) characterization. Besides the challenges in reaching relevant HER conditions in special cells compatible with different measurement techniques, the bulk sensitive nature of techniques such as Raman spectroscopy and XAS and short lifetime of intermediates makes detection of active sites a great challenge. In addition, due to the unknown structure of a-MoS_x_, uncertainties related to assignment of spectral features remain, which is rarely considered. For example, Sahu *et al.*^28^ have shown that the typical assignment of Raman/IR features based on (NH_4_^+^)_2_[Mo_3_S_13_]^2-^ may not be reliable even for neutral compounds of cluster type, let alone of possible chain structures.

Perhaps the most direct evidence of active sites comes from *operando* Raman studies of electrodeposited a-MoS_3.1_ and a-MoS_1.9_ by Deng *et al.*^15^, who observed formation of S-H species, while Mo-H was not observed. DFT calculations of S-H vibrational frequencies ruled out apical S^2-^ species, leaving S^2-^ (denoted unsaturated S^2-^ by the authors) as well as bridging and terminal S_2_^2-^ as possible active species. Selecting the active site from these possibilities is based on more indirect evidence. Deng *et al.* observed both terminal and bridging S_2_^2-^ modes to attenuate during HER. The bridging S_2_^2-^ atoms in Mo_3_S_y_ clusters, were speculated to be transformed to unsaturated S^2-^. In another study from the group, Ting *et al.*^14^ observed increased activity with increased content of bridging S_2_^2-^ and apical S^2-^ species. DFT calculations suggested H binding energy to be the most favorable on bridging S_2_^2-^ sites, which were then proposed as active sites. However, this control of S species was achieved by partial electrochemical oxidation of the films, which may also have other effects on the structure. This study, as well as the study by Lee *et al.*^37^ on selective removal of S species by thermal annealing, therefore provide somewhat indirect evidence on the role of different S species in the context of typical a-MoS_x_ catalysts deposited at low temperatures.

Electrochemical mass spectrometry combined with *operando* Raman studies on sputtered a-MoS_3.8_ films by Xi *et al.*^22^ instead found that terminal S_2_^2-^ species were converted to S^2-^. Notably, the authors observed release of H_2_S during the activation process. Rather than the newly formed S^2-^, however, Xi *et al.* suggested simultaneously generated unsaturated Mo to be the active site.

*Operando* XAS investigation of electrodeposited a-MoS_3_ by Lassalle-Kaiser *et al.*^60^ suggested reduction of Mo from 4+ to 3+ on the catalyst surface together with oxidation of sulfur, the latter assigned to increase of terminal S_2_^2-^. Although the changes in Mo and S edge positions were small, the authors to proposed a catalytic cycle, where Mo and S oxidation states change between +3 and +4 and -1 and -2, respectively. Another XAS study by Wu *et al.*^5^ observed no significant change in Mo oxidation state.

Besides investigations on a-MoS_x_, studies on Mo_x_S_y_ cluster compounds, which are commonly assumed to be the building blocks of a-MoS_x_, enable use of complementary characterization methods for insights into their activity. Baloglou *et al.*^61^ synthesized [HMo_3_S_13_]^-^ gas phase clusters by protonating the precursor cluster. The authors then observed S-H moieties and no Mo-H. With support of DFT calculations, they suggested that terminal S_2_^2-^ groups are the most favored to be protonated. In line with the above, recent advanced DFT studies of Abidi *et al.*^62^ suggested (hypothetical) Mo_3_S_9_ clusters to lose two sulfur atoms from terminal S_2_^2-^ groups, which can then yield active -SH or -OH sites.

The role of oxygen and accompanied Mo^5+^ sites, which are easily formed in air, have been suggested important yet appear to be based on rather indirect evidence. Kendall *et al.*^38^ suggested Mo^5+^ to increase HER activity, based on observation that decreasing Mo^5+^ content by annealing decreased HER activity. However, this may also be attributed to annealing-induced changes, as a-MoS_x_ are known to be unstable upon annealing.^18,29^ Interestingly, using *ex situ* XPS, Kendall *et al*.^38^ observed a state between Mo^4+^ and Mo^5+^ to increase during HER. Giuffredi *et al.*^21^, also suggesting important role of oxidized species, observed a similar rise of a new Mo component with a BE of 0.5 eV above Mo^4+^S. This is similar to our observations using quasi *in situ* XPS, suggesting these states might contribute to HER activity. Casalongue *et al.*^63^ performed atmospheric pressure, *operando* XPS measurements on a-MoS_3_ nanoparticles synthesized by wet chemistry. In line with *ex situ* XPS measurements performed by others, the authors observed a decrease of the higher BE S doublet. However, the Mo spectrum showed intense oxidation with most of Mo present as 6+ under HER conditions, which the authors attributed to a leak in the cell.

**Table S7**. Summary of selected investigations looking into structure and active sites of a-MoS_x_ catalysts in acid. DEMS = differential electrochemical mass spectrometry, DFT = density functional theory, EXAFS = Extended X-ray absorption fine structure. * indicates *operando* or *in situ* measurements

| Deposition method, initial (after) stoichiometry | Suggested active species | Evidence | Ref. |
| --- | --- | --- | --- |
| Electrodeposition, a-MoS_2.7_ and a-MoS_1.7_ | Bridging S_2_^2^ | XPS: bridging S_2_^2-^/apical S^2-^ ratio correlates with activity. DFT: H adsorption on apical S^2-^ is not favored. Raman*: bridging S_2_^2-^ suggested to be the most active | Ting *et al.* ^14^ |
| Electrodeposition, a-MoS_3.1_ and a-MoS_1.9_ | Unsaturated (bridging) S^2-^ and bridging S_2_^2-^ | Raman*: ν(S-H) detected, Mo-H not. Confirmed with isotope labeling. Terminal S_2_^2-^ disappeared. ν(S-H) position excludes apical S^2-^ | Deng *et al.* ^15^ |
| Wet chemical, a-MoS_4.3_ | Bridging S_2_^2-^ | Selective oxidation of S species: loss of bridging S_2_^2-^ (XPS) leads to decreased activity | Lee *et al.* ^37^ |
| Wet chemical, a-MoS_3.0_ | Unsaturated S | Based on MoS_2_ analogy | Vrubel *et al.* ^11^ |
| Electrodeposition, a-MoS_2.9_ | Terminal S_2_^2-^ bound to Mo^3+^ | EXAFS*: disappearance of Mo-Mo and bridging S_2_^2-^. Formation of Mo^3+^ observed | Lassalle-Kaiser *et al.* ^60^ |
| Electrodeposition and wet chemical, a-MoS_6_ (a-MoS_4.5_) and a-MoS_4_ | S_2_^2-^ | Higher S/Mo ratio leads to higher activity and contains more S_2_^2-^  🡪 suggested as active species | Mabayoje *et al.* ^12,13^ |
| Laser ablation, a-MoS_1.5_ to a-MoS_2.7_ | Mo^5+^ and bridging S_2_^2-^ | Raman: terminal S_2_^2-^ lost, bridging remains | Li *et al.* ^23^ |
| Electrodeposition, a-MoS_2.4_O_x_ | Unsaturated (bridging) S^2-^ and Mo^5+^ | Raman: terminal and bridging S_2_^2-^ lost, latter generates unsaturated S^2-^ | Escalera-López et al. ^16^ |
| Sputtering, a-MoS_3.8_ (a-MoS_1.9_) | Unsaturated Mo on MoS_2_ edges | Raman, TEM: MoS_2_ formed  Raman*, XPS: S_2_^2-^ removed leaving unsaturated Mo atoms  DEMS: H_2_S formed during activation | Xi *et al.* ^22^ |

#

# S9. Literature on HER activity of a-MoS_2+x_ catalysts

**Table S8**. Summary of literature on a-MoS_x_ HER catalysts and their activity and stability. Unless otherwise noted, HER measurements were performed in 0.5 M H_2_SO_4_. a) Estimated from reported thickness, density, and C_dl_. b) 0.1 M H_2_SO_4_. c) 1 M H_2_SO_4_. d) 1 M HClO_4_. e) 0.1 M HClO_4_, 10 mA/cm^2^ not reached. f) HER onset potential of Pt is approximately +50 mV vs RHE instead of its definition 0 V vs RHE, suggesting potential scale may be incorrect.

| Preparation, stoichiometry, substrate | Loading (µg/cm^2^) | η_10mA/cm2_ (mV) | Tafel slope (mV/dec) | Stability | Ref. |
| --- | --- | --- | --- | --- | --- |
| PEALD a-MoS_2.8_ to a-MoS_4.7_ thin films on GC and CFP | 2–3 | 208–246 | 41–51 | 24 h @ 10 mA/cm^2^  a-MoS_3.1_: 10 mV increase  a-MoS_4.5_: 100 mV increase | This |
| ALD a-MoS_1.9_ on CFP | ~100 ^a)^ | 250 | 56 | 1000 CVs: no change | ^3^ |
| ALD a-MoS_2.2_ on Au | 2.7 | 280 | 47 | 1000 CVs: 20 mV increase at 5 mA/cm^2^ | ^4^ |
| ALD a-MoS_2.3_ on GC |  | ~230 | 42 | 24 h @ 3 mA/cm^2^: 10 mV increase | ^5^ |
| ALD a-MoS_2.0_ on TiO_2_ nanotubes | 1200 | 189 |  | 36 h @ –0.45 V: ~10% increase in η^b)^ | ^6^ |
| ALD a-MoS_2.0_ on SnO_2_/Al_2_O_3_ nanotubes | 160 | 220 |  | 1 h @ ~50 mA/cm^2^ | ^7^ |
| ALD a-MoS on graphite fibers |  | 247 | 71 | 3000 CVs: 30 mV increase at 10 mA/cm^2^ | ^8^ |
| Electrodeposited on GC or FTO:  a-MoS_2.9_  a-MoS_3.2_  a-MoS_2.0_ | 200 | ~250 ^c)^  ~250 ^c)^  ~250 ^c)^ | 40 | Stable for 5 CVs  5 CVs: ~20 mV increase | ^9^ |
| Electrodeposited on GC:  a-MoS_2.9_  a-MoS_3.6_  a-MoS_2.1_ | 15  15  15 | 200 ^c)^  195 ^c)^  210 ^c)^ |  |  | ^10^ |
| Wet chemical a-MoS_3.0_:  on FTO  on GC  on MWCNT  Spray cast on FTO | 53  32  21  200 | ~330 ^c)^  ~260 ^c)^  ~220 ^c)^  ~250 ^c)^ | 54  42  40–45 |  | ^11^ |
| Electrodeposited on GC  a-MoS_6:_  a-MoS_4_  a-MoS_2_ | 90 | 161  182  212 |  | 3 h @ 10 mA/cm^2^: 20 mV increase | ^12^ |
| Wet chemical on GC:  a-MoS_6_  a-MoS_4_ | 1000 | 130  178 |  | 2 h @ –0.11 V: ~25% decrease in j | ^13^ |
| Electrodeposited on GC:  a-MoS_2.7_  a-MoS_1.7_ |  | ~240 ^d)^  ~260 ^d)^ | 38–40 | 0.5 h @ –0.2 V: ~40% decrease in j  0.5 h @ –0.2 V: ~50% decrease in j | ^14^ |
| Electrodeposited on GC:  a-MoS_3.1_  a-MoS_1.9_ |  | ~<250 ^d)^  ~<250 ^d)^ | 40  40 |  | ^15^ |
| Electrodeposited a-MoS_2.4_O_x_ on Au/Ti/SiO_2_/Si | 33.5 | 335 | 65 | 12 h @ 10 mA/cm^2^: 50 mV increase in η | ^16^ |
| Preparation, stoichiometry, substrate | Loading (µg/cm^2^) | η_10mA/cm2_ (mV) | Tafel slope (mV/dec) | Stability | Ref. |
| Wet chemical a-MoS_3_ on GC | >~1000 | 200 | 60 | 10 000 CVs: 57 mV increase in η @ 10 mA/cm^2^ | ^17^ |
| Wet chemical a-MoS_3_ on GC | 50 | 210 | ? |  | ^18^ |
| Solvothermal on graphite sheets:  a-MoS_2.6_  a-MoS_3.4_ |  | 235  245 | 100  90 | 7 h @ 3 mA/cm^2^: 20 mV increase  7 h @ 3 mA/cm^2^: 15 mV increase | ^19^ |
| Wet chemical MoS_x_:  On GC  on CNTs | 102 | 210  110 ^f)^ | 60  40 | 1000 CVs: 10 mV increase @ 10 mA/cm^2^ | ^20^ |
| PLD a-MoS_3.8_ on GC | 300 | 126 | 35 | 120 h @ 100 mA/cm^2^: no change | ^21^ |
| Sputtered a-MoS_3.8_ on FTO | ~100 | 180 | ~40 | 10 h CVs: 30 mV increase @ 10 mA/cm^2^ | ^22^ |
| Wet chemical on GC:  a-MoS_2.7_  a-MoS_1.5_ | 150  150 | 145  220 | 40  56 | 2 h @ –0.15 V: 15% decrease in j  2 h @ –0.15 V: 20% decrease in j | ^23^ |
| Thermolysis on carbon cloth:  a-MoS_3.1_  a-MoS_1.7_ (H_2_ plasma treated) | 1000  1000 | 206  143 | 84  40 | 24 h at ~1 A/cm^2^: <10% decrease in j | ^24^ |

# S10. References

(1) Scofield, J. H. Hartree-Slater Subshell Photoionization Cross-Sections at 1254 and 1487 eV. *J. Electron Spectros. Relat. Phenomena* **1976**, *8*, 129–137.

(2) Mattinen, M.; Gity, F.; Coleman, E.; Vonk, J. F. A.; Verheijen, M. A.; Duffy, R.; Kessels, W. M. M.; Bol, A. A. Atomic Layer Deposition of Large-Area Polycrystalline Transition Metal Dichalcogenides from 100 °C through Control of Plasma Chemistry. *Chem. Mater.* **2022**, *34*, 7280–7292.

(3) Kwon, D. H.; Jin, Z.; Shin, S.; Lee, W.-S.; Min, Y.-S. Comprehensive Study on Atomic Layer Deposition of Molybdenum Sulfide for Electrochemical Hydrogen Evolution. *Nanoscale* **2016**, *8*, 7180–7188.

(4) Shin, S.; Jin, Z.; Kwon, D. H.; Bose, R.; Min, Y.-S. High Turnover Frequency of Hydrogen Evolution Reaction on Amorphous MoS_2_ Thin Film Directly Grown by Atomic Layer Deposition. *Langmuir* **2015**, *31*, 1196–1202.

(5) Wu, L.; Longo, A.; Dzade, N. Y.; Sharma, A.; Hendrix, M. M. R. M.; Bol, A. A.; de Leeuw, N. H.; Hensen, E. J. M.; Hofmann, J. P. The Origin of High Activity of Amorphous MoS_2_ in the Hydrogen Evolution Reaction. *ChemSusChem* **2019**, *12*, 4383–4389.

(6) Cao, Y.; Wu, Y.; Badie, C.; Cadot, S.; Camp, C.; Quadrelli, E. A.; Bachmann, J. Electrocatalytic Performance of Titania Nanotube Arrays Coated with MoS_2_ by ALD toward the Hydrogen Evolution Reaction. *ACS Omega* **2019**, *4*, 8816–8823.

(7) Englhard, J.; Cao, Y.; Bochmann, S.; Barr, M. K. S.; Cadot, S.; Quadrelli, E. A.; Bachmann, J. Stabilizing an Ultrathin MoS_2_ Layer during Electrocatalytic Hydrogen Evolution with a Crystalline SnO_2_ Underlayer. *RSC Adv.* **2021**, *11*, 17985–17992.

(8) Teich, J.; Dvir, R.; Henning, A.; Hamo, E. R.; Moody, M. J.; Jurca, T.; Cohen, H.; Marks, T. J.; Rosen, B. A.; Jauhon, L. J.; Ismach, A. Light and Complex 3D MoS_2_/Graphene Heterostructures as Efficient Catalysts for the Hydrogen Evolution Reaction. *Nanoscale* **2020**, *12*, 2715–2725.

(9) Merki, D.; Fierro, S.; Vrubel, H.; Hu, X. Amorphous Molybdenum Sulfide Films as Catalysts for Electrochemical Hydrogen Production in Water. *Chem. Sci.* **2011**, *2*, 1262–1267.

(10) Vrubel, H.; Hu, X. Growth and Activation of an Amorphous Molybdenum Sulfide Hydrogen Evolving Catalyst. *ACS Catal.* **2013**, *3*, 2002–2011.

(11) Vrubel, H.; Merki, D.; Hu, X. Hydrogen Evolution Catalyzed by MoS_3_ and MoS_2_ Particles. *Energy Environ. Sci.* **2012**, *5*, 6136–6144.

(12) Mabayoje, O.; Liu, Y.; Wang, M.; Shoola, A.; Ebrahim, A. M.; Frenkel, A. I.; Mullins, C. B. Electrodeposition of MoS_x_ Hydrogen Evolution Catalysts from Sulfur-Rich Precursors. *ACS Appl. Mater. Interfaces* **2019**, *11*, 32879–32886.

(13) Mabayoje, O.; Wygant, B. R.; Wang, M.; Liu, Y.; Mullins, C. B. Sulfur-Rich MoS_6_ as an Electrocatalyst for the Hydrogen Evolution Reaction. *ACS Appl. Energy Mater.* **2018**, *1*, 4453–4458.

(14) Ting, L. R. L.; Deng, Y.; Ma, L.; Zhang, Y. J.; Peterson, A. A.; Yeo, B. S. Catalytic Activities of Sulfur Atoms in Amorphous Molybdenum Sulfide for the Electrochemical Hydrogen Evolution Reaction. *ACS Catal.* **2016**, *6*, 861–867.

(15) Deng, Y.; Ting, L. R. L.; Neo, P. H. L.; Zhang, Y. J.; Peterson, A. A.; Yeo, B. S. Operando Raman Spectroscopy of Amorphous Molybdenum Sulfide (MoS_x_) during the Electrochemical Hydrogen Evolution Reaction: Identification of Sulfur Atoms as Catalytically Active Sites for H^+^ Reduction. *ACS Catal.* **2016**, *6*, 7790–7798.

(16) Escalera-López, D.; Lou, Z.; Rees, N. V. Benchmarking the Activity, Stability, and Inherent Electrochemistry of Amorphous Molybdenum Sulfide for Hydrogen Production. *Adv. Energy Mater.* **2019**, *9*, 1802614.

(17) Benck, J. D.; Chen, Z.; Kuritzky, L. Y.; Forman, A. J.; Jaramillo, T. F. Amorphous Molybdenum Sulfide Catalysts for Electrochemical Hydrogen Production: Insights into the Origin of Their Catalytic Activity. *ACS Catal.* **2012**, *2*, 1916–1923.

(18) Lee, S. C.; Benck, J. D.; Tsai, C.; Park, J.; Koh, A. L.; Abild-Pedersen, F.; Jaramillo, T. F.; Sinclair, R. Chemical and Phase Evolution of Amorphous Molybdenum Sulfide Catalysts for Electrochemical Hydrogen Production. *ACS Nano* **2016**, *10*, 624–632.

(19) Ronge, E.; Hildebrandt, S.; Grutza, M.; Klein, H.; Kurz, P.; Jooss, C. Structure of Nanocrystalline, Partially Disordered MoS_2+δ_ Derived from HRTEM - An Abundant Material for Efficient HER Catalysis. *Catalysts* **2020**, *10*, 856.

(20) Li, D. J.; Maiti, U. N.; Lim, J.; Choi, D. S.; Lee, W. J.; Oh, Y.; Lee, G. Y.; Kim, S. O. Molybdenum Sulfide/N-Doped CNT Forest Hybrid Catalysts for High-Performance Hydrogen Evolution Reaction. *Nano Lett.* **2014**, *14*, 1228–1233.

(21) Giuffredi, G.; Mezzetti, A.; Perego, A.; Mazzolini, P.; Prato, M.; Fumagalli, F.; Lin, Y. C.; Liu, C.; Ivanov, I. N.; Belianinov, A.; Colombo, M.; Divitini, G.; Ducati, C.; Duscher, G.; Puretzky, A. A.; Geohegan, D. B.; di Fonzo, F. Non-Equilibrium Synthesis of Highly Active Nanostructured, Oxygen-Incorporated Amorphous Molybdenum Sulfide HER Electrocatalyst. *Small* **2020**, *16*, 2004047.

(22) Xi, F.; Bogdanoff, P.; Harbauer, K.; Plate, P.; Höhn, C.; Rappich, J.; Wang, B.; Han, X.; van de Krol, R.; Fiechter, S. Structural Transformation Identification of Sputtered Amorphous MoS_x_ as an Efficient Hydrogen-Evolving Catalyst during Electrochemical Activation. *ACS Catal.* **2019**, *9*, 2368–2380.

(23) Li, B.; Jiang, L.; Li, X.; Cheng, Z.; Ran, P.; Zuo, P.; Qu, L.; Zhang, J.; Lu, Y. Controllable Synthesis of Nanosized Amorphous MoS_x_ Using Temporally Shaped Femtosecond Laser for Highly Efficient Electrochemical Hydrogen Production. *Adv. Funct. Mater.* **2019**, *29*, 1806229.

(24) Lu, A. Y.; Yang, X.; Tseng, C. C.; Min, S.; Lin, S. H.; Hsu, C. L.; Li, H.; Idriss, H.; Kuo, J. L.; Huang, K. W.; Li, L. J. High-Sulfur-Vacancy Amorphous Molybdenum Sulfide as a High Current Electrocatalyst in Hydrogen Evolution. *Small* **2016**, *12*, 5530–5537.

(25) Afanasiev, P. Synthetic Approaches to the Molybdenum Sulfide Materials. *Comptes Rendus Chim.* **2008**, *11*, 159–182.

(26) Grayfer, E. D.; Artemkina, S. B.; Ivanova, M. N.; Brylev, K. A.; Fedorov, V. E. Low-Dimensional Group IV–VII Transition Metal Polychalcogenides and Chemical Aspects of Their Applications. *Russ. Chem. Rev.* **2023**, *92*, RCR5072.

(27) Chang, C.; Wang, L.; Xie, L.; Zhao, W.; Liu, S.; Zhuang, Z.; Liu, S.; Li, J.; Liu, X.; Zhao, W. Amorphous Molybdenum Sulfide and Its Mo-S Motifs: Structural Characteristics, Synthetic Strategies, and Comprehensive Applications. *Nano Res.* **2022**, *15*, 8613–8635.

(28) Sahu, A.; Steinmann, S. N.; Raybaud, P. Size-Dependent Structural, Energetic, and Spectroscopic Properties of MoS_3_ Polymorphs. *Cryst. Growth Des.* **2020**, *20*, 7750–7760.

(29) Weber, T.; Muijsers, J. C.; Niemantsverdriet, J. W. Structure of Amorphous MoS_3_. *J. Phys. Chem.* **1995**, *99*, 9194–9200.

(30) Müller, A.; Josters, R.; Eltzner, W.; Chonjg-Shi, N.; Diemann, E.; Bögge, H.; Zimmermann, M.; Dartmann, M.; Reinsch-Vogell, U.; Che, S.; Cyvin, S. J.; Cyvin, B. N. Synthetic, Spectroscopic, x-Ray Structural, and Quantum-Chemical Studies of Cyanothiomolybdates with Mo_2_S_2_, Mo_3_S_4_ and Mo_4_S_4_ Cores: A Remarkable Class of Species Existing with Different Electron Populations and Having the Same Central Units as the Ferredoxins. *Inorg. Chem.* **1985**, *19*, 2872–2884.

(31) Müller, A.; Diemann, E.; Krickemeyer, E.; Walberg, H. J.; Bögge, H.; Armatage, A. [MO_3_(IV)S(S_2_)_6_]^2-^ From Amorphous MoS_3_ by the Reaction with OH^-^ and Structure of (NH_4_)_2_[MO_3_(IV)S(S_2_)_6_]·H_2_O. *Eur. J. Solid State Inorg. Chem.* **1993**, *30*, 565–572.

(32) Müller, A.; Fedin, V.; Hegetschweiler, K.; Amrein, W. Characterization of Amorphous Substances by Studying Isotopically Labelled Compounds with FAB-MS: Evidence for Extrusion of Triangular Mo_3_IV Clusters from a Mixture of ^92^MoS_3_ and ^100^MoS_3_ by Reaction with OH^-^. *J. Chem. Soc. Chem. Commun.* **1992**, *7*, 1795–1796.

(33) Müller, A.; Diemann, E. Polysulfide Complexes of Metals. *Adv. Inorg. Chem.* **1987**, *31*, 89–122.

(34) Fedorov, V. E.; Mironov, Y. V; Naumov, N. G.; Sokolov, M. N.; Fedin, V. P. Chalcogenide Clusters of Group 5–7 Metals. *Russ. Chem. Rev.* **2007**, *76*, 529–552.

(35) Tran, P. D.; Tran, T. V.; Orio, M.; Torelli, S.; Truong, Q. D.; Nayuki, K.; Sasaki, Y.; Chiam, S. Y.; Yi, R.; Honma, I.; Barber, J.; Artero, V. Coordination Polymer Structure and Revisited Hydrogen Evolution Catalytic Mechanism for Amorphous Molybdenum Sulfide. *Nat. Mater.* **2016**, *15*, 640–646.

(36) Hibble, S. J.; Feaviour, M. R. An in Situ Structural Study of the Thermal Decomposition Reactions of the Ammonium Thiomolybdates, (NH_4_)_2_Mo_2_S_12_·2H_2_O and (NH_4_)_2_Mo_3_S_13_·2H_2_O. *J. Mater. Chem.* **2001**, *11*, 2607–2614.

(37) Lee, C. H.; Lee, S.; Lee, Y. K.; Jung, Y. C.; Ko, Y. Il; Lee, D. C.; Joh, H. I. Understanding the Origin of Formation and Active Sites for Thiomolybdate [Mo_3_S_13_]^2-^ Clusters as Hydrogen Evolution Catalyst through the Selective Control of Sulfur Atoms. *ACS Catal.* **2018**, *8*, 5221–5227.

(38) Kendall, L.; Chamaani, A.; Piontkowski, Z.; Beechem, T. E.; Ridley, M.; Opila, E. J.; Zangari, G.; McDonnell, S. J. Influence of Oxygen Dopants on the HER Catalytic Activity of Electrodeposited MoO_x_S_y_ Electrocatalysts. *ACS Appl. Energy Mater.* **2021**, *4*, 13676–13683.

(39) Seo, B.; Jung, G. Y.; Lee, S. J.; Baek, D. S.; Sa, Y. J.; Ban, H. W.; Son, J. S.; Park, K.; Kwak, S. K.; Joo, S. H. Monomeric MoS_4_^2-^-Derived Polymeric Chains with Active Molecular Units for Efficient Hydrogen Evolution Reaction. *ACS Catal.* **2020**, *10*, 652–662.

(40) Ji, Z.; Trickett, C.; Pei, X.; Yaghi, O. M. Linking Molybdenum-Sulfur Clusters for Electrocatalytic Hydrogen Evolution. *J. Am. Chem. Soc.* **2018**, *140*, 13618–13622.

(41) Kibsgaard, J.; Jaramillo, T. F.; Besenbacher, F. Building an Appropriate Active-Site Motif into a Hydrogen-Evolution Catalyst with Thiomolybdate [Mo_3_S_13_]^2-^ Clusters. *Nat. Chem.* **2014**, *6*, 248–253.

(42) Hellstern, T. R.; Kibsgaard, J.; Tsai, C.; Palm, D. W.; King, L. A.; Abild-Pedersen, F.; Jaramillo, T. F. Investigating Catalyst-Support Interactions to Improve the Hydrogen Evolution Reaction Activity of Thiomolybdate [Mo_3_S_13_]^2-^ Nanoclusters. *ACS Catal.* **2017**, *7*, 7126–7130.

(43) Liang, K. S.; Cramer, S. P.; Johnston, D. C.; Chang, C. H.; Jacobson, A. J.; Deneufville, J. P.; Chianelli, R. R. Amorphous MoS_3_ AND WS_3_. *J. Non. Cryst. Solids* **1980**, *42*, 345–356.

(44) Cramer, S. P.; Liang, K. S.; Jacobson, A. J.; Chang, C. H.; Chianelli, R. R. EXAFS Studies of Amorphous Molybdenum and Tungsten Trisulfides and Triselenides. *Inorg. Chem.* **1984**, *23*, 1215–1221.

(45) Chien, F. Z.; Moss, S. C.; Liang, K. S.; Chianelli, R. R. Local and Intermediate-Range Structure of Amorphous MoS_3_: Model Calculation Study. *Phys. Rev. B* **1984**, *29*, 4606–4615.

(46) Walton, R. I.; Dent, A. J.; Hibble, S. J. In Situ Investigation of the Thermal Decomposition of Ammonium Tetrathiomolybdate Using Combined Time-Resolved X-Ray Absorption Spectroscopy and X-Ray Diffraction. *Chem. Mater.* **1998**, *19*, 3737–3745.

(47) Hibble, S. J.; Walton, R. I.; Pickup, D. M.; Hannon, A. C. Amorphous MoS_3_: Clusters or Chains? The Structural Evidence. *J. Non. Cryst. Solids* **1998**, *232*–*234*, 434–439.

(48) Hibble, S. J.; Wood, G. B. Modeling the Structure of Amorphous MoS_3_: A Neutron Diffraction and Reverse Monte Carlo Study. *J. Am. Chem. Soc.* **2004**, *126*, 959–965.

(49) Hibble, S. J.; Pickup, D. M.; Hannon, A. C. The Structures of the New Amorphous Transition Metal Chalcogenides MoS_4.65_, WS_5_ and CrSe_3_. *Phys. Scr.* **1995**, *T57*, 94–97.

(50) Hibble, S. J.; Rice, D. A.; Pickup, D. M.; Beer, M. P. Mo K-Edge EXAFS and S K-Edge Absorption Studies of the Amorphous Molybdenum Sulfides MoS_4.7_, MoS_3_, and MoS_3_·nH_2_O (n ~ 2). *Inorg. Chem.* **1995**, *34*, 5109–5113.

(51) Artemkina, S. B.; Enyashin, A. N.; Poltarak, A. A.; Fedorenko, A. D.; Makarova, A. A.; Poltarak, P. A.; Shin, E. J.; Hwang, S. J.; Kim, S. J.; Grayfer, E. D.; Fedorov, V. E. Revealing the Flexible 1D Primary and Globular Secondary Structures of Sulfur-Rich Amorphous Transition Metal Polysulfides. *ChemNanoMat* **2019**, *5*, 1488–1497.

(52) Afanasiev, P.; Bezverkhy, I. Synthesis of MoS_x_ (5 > x > 6) Amorphous Sulfides and Their Use for Preparation of MoS_2_ Monodispersed Microspheres. *Chem. Mater.* **2002**, *14*, 2826–2830.

(53) Poltarak, A. A.; Logvinenko, V. A.; Enyashin, A. N.; Artemkina, S. B.; Poltarak, P. A.; Ivanova, M. N.; Grayfer, E. D.; Fedorov, V. E. Thermal and Kinetic Studies of Sulfur-Rich Molybdenum and Tungsten Polysulfides. *J. Alloys Compd.* **2021**, *851*, 156705.

(54) Hibble, S. J.; Feaviour, M. R.; Almond, M. J. Chemical Excision from Amorphous MoS_3_; a Quantitative EXAFS Study. *J. Chem. Soc. Dalt. Trans.* **2001**, 935–940.

(55) Afanasiev, P.; Jobic, H.; Lorentz, C.; Leverd, P.; Mastubayashi, N.; Piccolo, L.; Vrinat, M. Low-Temperature Hydrogen Interaction with Amorphous Molybdenum Sulfides MoS_x_. *J. Phys. Chem. C* **2009**, *113*, 4139–4146.

(56) Jaramillo, T. F.; Jørgensen, K. P.; Bonde, J.; Nielsen, J. H.; Horch, S.; Chorkendorff, I. Identification of Active Edge Sites for Electrochemical H_2_ Evolution from MoS_2_ Nanocatalysts. *Science* **2007**, *317*, 100–102.

(57) Hinnemann, B.; Moses, P. G.; Bonde, J.; Jørgensen, K. P.; Nielsen, J. H.; Horch, S.; Chorkendorff, I.; Nørskov, J. K. Biomimetic Hydrogen Evolution: MoS_2_ Nanoparticles as Catalyst for Hydrogen Evolution. *J. Am. Chem. Soc.* **2005**, *127*, 5308–5309.

(58) Cotton, F. A.; Dori, Z.; Llusar, R.; Schwotzer, W. The Mo_3_S_4_^+^ Aquo Ion. *J. Am. Chem. Soc.* **1985**, *107*, 6734–6735.

(59) Huang, Z.; Luo, W.; Ma, L.; Yu, M.; Ren, X.; He, M.; Polen, S.; Click, K.; Garrett, B.; Lu, J.; Amine, K.; Hadad, C.; Chen, W.; Asthagiri, A.; Wu, Y. Dimeric [Mo_2_S_12_]^2-^ Cluster: A Molecular Analogue of MoS_2_ Edges for Superior Hydrogen-Evolution Electrocatalysis. *Angew. Chemie - Int. Ed.* **2015**, *54*, 15181–15185.

(60) Lassalle-Kaiser, B.; Merki, D.; Vrubel, H.; Gul, S.; Yachandra, V. K.; Hu, X.; Yano, J. Evidence from in Situ X-Ray Absorption Spectroscopy for the Involvement of Terminal Disulfide in the Reduction of Protons by an Amorphous Molybdenum Sulfide Electrocatalyst. *J. Am. Chem. Soc.* **2015**, *137*, 314–321.

(61) Baloglou, A.; Plattner, M.; Ončák, M.; Grutza, M. L.; Kurz, P.; Beyer, M. K. [Mo_3_S_13_]^2−^ as a Model System for Hydrogen Evolution Catalysis by MoS_x_: Probing Protonation Sites in the Gas Phase by Infrared Multiple Photon Dissociation Spectroscopy. *Angew. Chemie - Int. Ed.* **2021**, *60*, 5074–5077.

(62) Abidi, N.; Sahu, A.; Raybaud, P.; Steinmann, S. N. Electrochemical Potential-Dependent Stability and Activity of MoS_3_ during the Hydrogen Evolution Reaction. *ACS Catal.* **2023**, *13*, 15290–15300.

(63) Casalongue, H. G. S.; Benck, J. D.; Tsai, C.; Karlsson, R. K. B.; Kaya, S.; Ng, M. L.; Pettersson, L. G. M.; Abild-Pedersen, F.; Nørskov, J. K.; Ogasawara, H.; Jaramillo, T. F.; Nilsson, A. Operando Characterization of an Amorphous Molybdenum Sulfide Nanoparticle Catalyst during the Hydrogen Evolution Reaction. *J. Phys. Chem. C* **2014**, *118*, 29252–29259.
